# Automatic cardiothoracic ratio calculation based on lung fields abstracted from chest X-ray images without heart segmentation

**DOI:** 10.3389/fphys.2024.1416912

**Published:** 2024-08-08

**Authors:** Yingjian Yang, Jie Zheng, Peng Guo, Tianqi Wu, Qi Gao, Yingwei Guo, Ziran Chen, Chengcheng Liu, Zhanglei Ouyang, Huai Chen, Yan Kang

**Affiliations:** ^1^ Department of Radiological Research and Development, Shenzhen Lanmage Medical Technology Co., Ltd., Shenzhen, Guangdong, China; ^2^ Neusoft Medical System Co., Ltd., Shenyang, Liaoning, China; ^3^ School of Electrical and Information Engineering, Northeast Petroleum University, Daqing, China; ^4^ College of Medicine and Biological Information Engineering, Northeastern University, Shenyang, China; ^5^ School of Life and Health Management, Shenyang City University, Shenyang, China; ^6^ Department of Radiology, The Second Affiliated Hospital of Guangzhou Medical University, Guangzhou China; ^7^ College of Health Science and Environmental Engineering, Shenzhen Technology University, Shenzhen China; ^8^ School of Applied Technology, Shenzhen University, Shenzhen, China; ^9^ Engineering Research Centre of Medical Imaging and Intelligent Analysis, Ministry of Education, Shenyang, China

**Keywords:** cardiothoracic ratio, chest X-ray images, lung field segmentation, edge detection, convolutional neural network, graphics

## Abstract

**Introduction:**

The cardiothoracic ratio (CTR) based on postero-anterior chest X-rays (P-A CXR) images is one of the most commonly used cardiac measurement methods and an indicator for initially evaluating cardiac diseases. However, the hearts are not readily observable on P-A CXR images compared to the lung fields. Therefore, radiologists often manually determine the CTR’s right and left heart border points of the adjacent left and right lung fields to the heart based on P-A CXR images. Meanwhile, manual CTR measurement based on the P-A CXR image requires experienced radiologists and is time-consuming and laborious.

**Methods:**

Based on the above, this article proposes a novel, fully automatic CTR calculation method based on lung fields abstracted from the P-A CXR images using convolutional neural networks (CNNs), overcoming the limitations to heart segmentation and avoiding errors in heart segmentation. First, the lung field mask images are abstracted from the P-A CXR images based on the pre-trained CNNs. Second, a novel localization method of the heart’s right and left border points is proposed based on the two-dimensional projection morphology of the lung field mask images using graphics.

**Results:**

The results show that the mean distance errors at the *x*-axis direction of the CTR’s four key points in the test sets T1 (21 × 512 × 512 static P-A CXR images) and T2 (13 × 512 × 512 dynamic P-A CXR images) based on various pre-trained CNNs are 4.1161 and 3.2116 pixels, respectively. In addition, the mean CTR errors on the test sets T1 and T2 based on four proposed models are 0.0208 and 0.0180, respectively.

**Discussion:**

Our proposed model achieves the equivalent performance of CTR calculation as the previous CardioNet model, overcomes heart segmentation, and takes less time. Therefore, our proposed method is practical and feasible and may become an effective tool for initially evaluating cardiac diseases.

## 1 Introduction

X-ray is the most widely used primary imaging technique for routine chest and bone radiography as it is widely available, low cost, has fast imaging speed, and easy to acquire (Liu et al., 2022). Because of its fast imaging (seconds after exposure), X-ray has become the preferred imaging device to improve the work efficiency and facilitate the diagnosis of critically ill and/or emergency patients in clinical practice (Howell, 2016; Seah et al., 2021).

The relationship analysis between the heart and lungs has been a hot topic in clinical or scientific research (Yang et al., 2022). The cardiothoracic ratio (CTR) is one of the most commonly used cardiac measurement methods and a commonly used indicator for evaluating cardiac enlargement (Saul, 1919; Hada, 1995; Chang et al., 2022). Specifically, various etiologies, such as pathological changes in the heart itself and increased adaptability secondary to hemodynamic changes leading to left and right heart enlargement, will increase the CTR. Chest X-rays (CXRs), as an economical and convenient routine examination, can better display the situation of the chest, lung tissue, pulmonary blood vessels, heart, chest blood vessels, etc., providing a reliable basis for clinical diagnosis (Howell, 2016; Seah et al., 2021; Liu et al., 2022). Although cardiac enlargement should be diagnosed through echocardiography, follow-up and treatment can be based on the postero-anterior (P-A) CXR images. P-A CXR must be performed for the initial cardiac examination (Hada, 1995). In addition, the CTR is also a predictor of heart failure progression in asymptomatic patients with cardiac diseases (Nochioka et al., 2023). Therefore, the accurate CTR measurement of these vulnerable populations is crucial for precision healthcare.

Manual CTR measurement based on the P-A CXR image requires experienced radiologists and is time-consuming and laborious. Therefore, with the rapid development of artificial intelligence, such as convolutional neural networks (CNNs), automatic CTR calculation methods or models based on P-A CXR images have been successively proposed in recent years (Li et al., 2019; Saiviroonporn et al., 2021; Jafar et al., 2022). Li et al. proposed a computer-aided technique that is more reliable and time- and labor-saving than the manual method in CTR calculation. In addition, Pairash et al. verified that the AI (artificial intelligence)-only method could replace the manual CTR measurement. Meanwhile, Abbas et al. proposed a CardioNet model that achieved acceptable accuracy and competitive results across all datasets. However, the above methods or models in references 9–11 are still limited by heart segmentation. Although heart segmentation techniques based on P-A CXR images have made significant progress (Lyu and Tian, 2023), whether the CTR calculation requires the specific morphology and structure of the heart remains to be studied. Anatomically, the heart is located within the chest cavity between the left and right lungs. Specifically, about one-third of the heart is on the right side of the midline, about two-thirds is on the left side, and the apex is on the lower left front (Weinhaus and Roberts, 2005). The transverse diameter of the heart in the cardiothoracic ratio refers to the sum of the maximum distances from the left and right cardiac margins to the midline. However, the heart in the P-A CXR images is not prominent. Therefore, if the heart segmentation needs to be more precise, the error of this heart segmentation may fail to calculate the CTR automatically based on the P-A CXR images.

Based on the above, a novel, fully automatic CTR calculation method based on the lung field should be proposed to overcome the limitations to heart segmentation. Specifically, we train a robust and standard segmentation model of pathological lungs based on multi-center training datasets of the P-A CXR images and image enhancement techniques for extracting lung fields in P-A CXR images. Then, the CTR is automatically calculated based on the lung field based on graphics. Our contributions in this paper are briefly described as follows:

(1) We propose a fully automatic CTR calculation method based on lung fields abstracted from the P-A CXR images using CNNs, overcoming the limitations to heart segmentation, avoiding errors in heart segmentation, and taking less time.
(2) We propose a novel localization method of the heart’s right and left border points based on the two-dimensional projection morphology of the lung field mask images using graphics.
(3) The proposed automatic CTR calculation method based on lung fields abstracted from the P-A CXR images may become an effective tool for initially evaluating cardiac diseases.

## 2 Materials and methods

### 2.1 Materials

Here, 789 (635 + 54 + 72 + 15 + 13) sets of the P-A CXR images from public CXR datasets, the Google website, and a case of P-A CXR video are collected in the study for training the CNNs of lung field segmentation and automatic CTR calculation. Specifically, 21 P-A CXR images are used as a test set for evaluating lung field segmentation models. In addition, these 21 P-A CXR images and 13 dynamic P-A CXR images are used to calculate the CTR.

**FIGURE 1:**
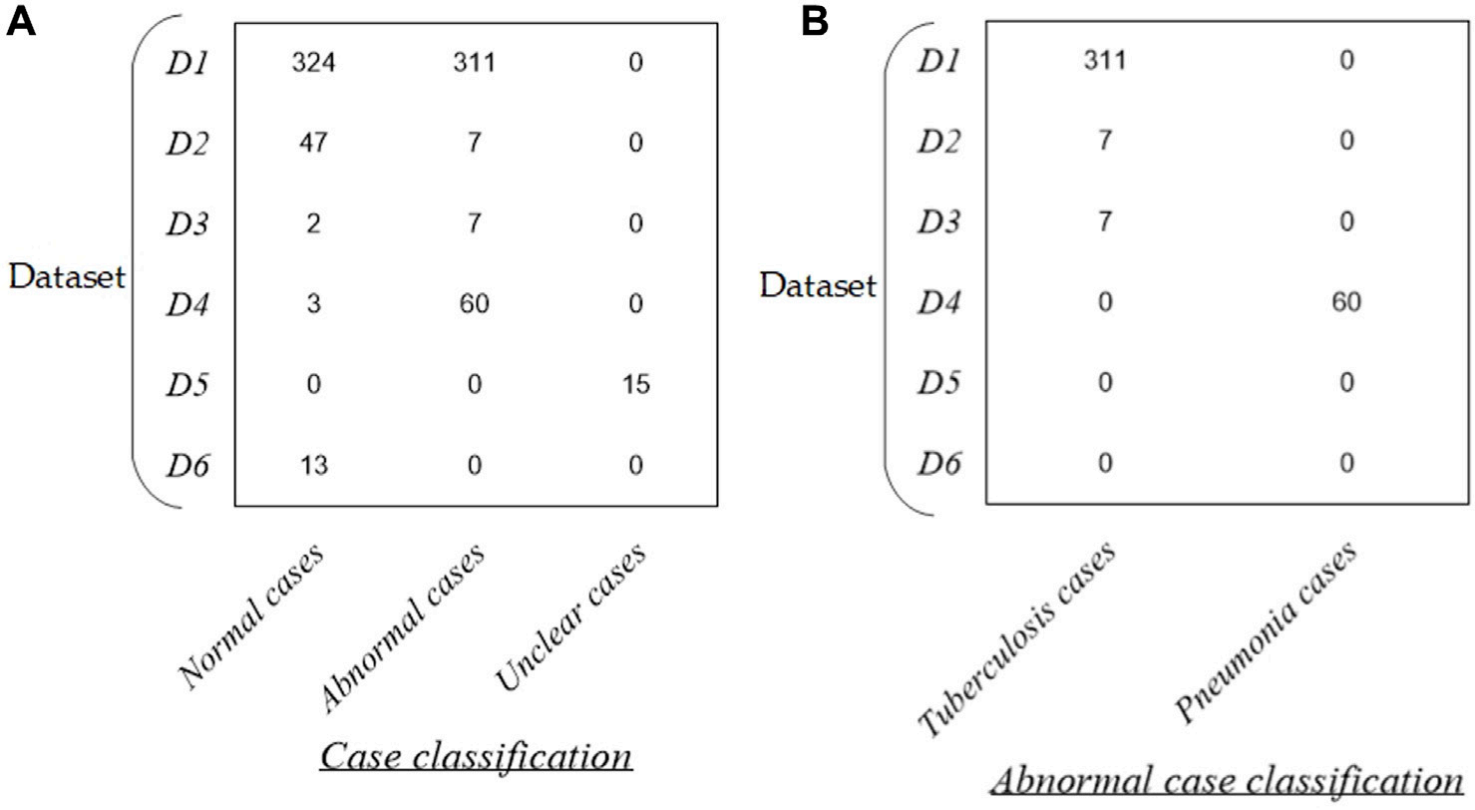
Distribution of the dataset in this study. **(A)** Distribution of case classification in each dataset and **(B)** distribution of abnormal cases in each dataset.

Figure 1 intuitively shows the detailed distribution of these 789 P-A CXR images in each dataset. Specifically, the dataset used in this study includes six sub-datasets (D1–D6). The public dataset D1 (The Shenzhen set — Chest X-ray database) includes 635 static P-A CXR images (324 normal cases and 311 cases with manifestations of tuberculosis). The public dataset D2 (The Shenzhen set — Chest X-ray database) includes 54 static P-A CXR images (47 normal cases and 7 cases with manifestation of tuberculosis). The public dataset D3 [NIAID TB portal program dataset (Online)] and D4 [kaggle. RSNA Pneumonia Detection Challenge (Online)] include 72 static P-A CXR images (7 cases with the manifestation of tuberculosis, 60 cases with the manifestation of pneumonia, and 5 normal cases). The public dataset D5 includes 15 static P-A CXR images collected from the Google website.

In addition, dataset D6 includes 13 dynamic P-A CXR images from the case of CXR video that were collected during free breathing. Specifically, the CXR video was collected from a female participant aged 53 using a digital X-ray imaging system (manufacturer: Lanmage, collection mode: sequence point slice, exposure parameters: 78 KV, 200 mA, 50 ms, and flat panel detector: IRAY) for chest photography. The ethics committee of the National Clinical Research Center for Respiratory Diseases in China approved this study. The subject has been provided written informed consent to the second affiliated hospital of Guangzhou Medical University before chest photography.

More specifically, the public CXR datasets D1 and D2 are collected from the website https://www.kaggle.com/datasets/kmader/pulmonary-chest-xray-abnormalities?select=ChinaSet_AllFiles. Meanwhile, the public CXR datasets D3 and D4 are collected from the websites https://data.tbportals.niaid.nih.gov/ and https://www.kaggle.com/c/rsna-pneumonia-detection-challenge/data, respectively.

### 2.2 Methods

**FIGURE 2:**
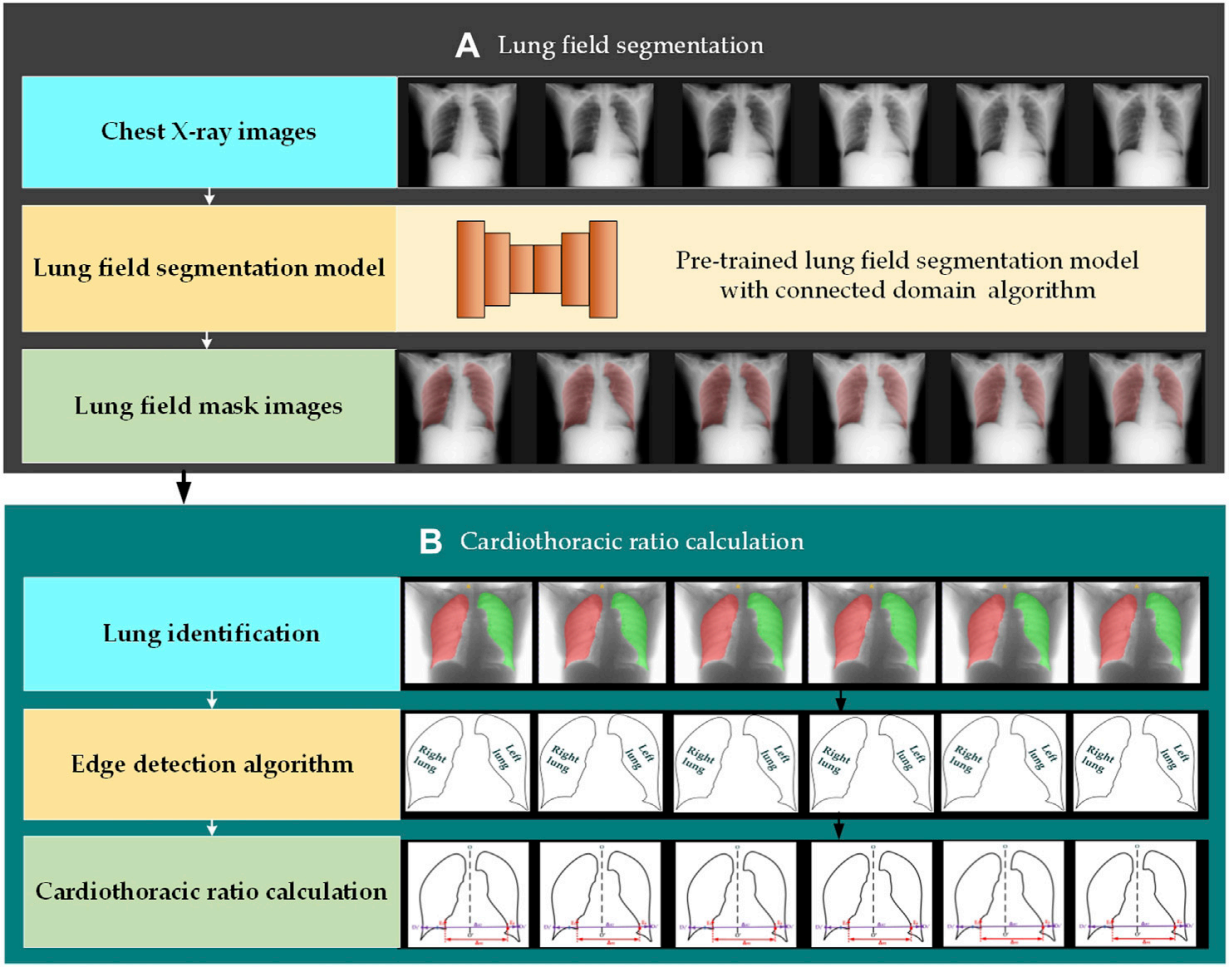
Automatic cardiothoracic ratio algorithm schematic diagram. **(A)** Lung field segmentation and **(B)** cardiothoracic ratio calculation.

Figure 2 intuitively shows the schematic diagram of the automatic cardiothoracic ratio algorithm. Specifically, the proposed fully automatic CTR calculation method based on lung field abstracted from the P-A CXR images includes lung field segmentation and cardiothoracic ratio calculation. Figure 2A shows that the lung field mask images are abstracted from the P-A CXR images based on the trained CNNs with the connected domain (CD) algorithm. Meanwhile, Figure 2B shows the automatic CTR calculation method based on the lung field mask images.

#### 2.2.1 Lung field segmentation

The organ segmentation model of medical images based on CNNs has become an indispensable technology for quantitative analysis (Conze et al., 2023; Jiang et al., 2023; Ma et al., 2024). CNNs have even been applied to the lung segmentation of rats for measuring lung parenchyma parameters (Yang et al., 2021). In addition, automatic lung field segmentation in routine imaging is a data diversity problem not a methodology problem (Hofmanninger et al., 2020).

The SegNet, U-Net, and its improved networks have been widely applied in organ segmentation of medical images. Based on the above, we train four traditional and basic CNNs to test whether different CNN lung field segmentation models have differences in CTR calculation, including SegNet (Badrinarayanan et al., 2017), U-Net (Ronneberger et al., 2015), and its two improved networks, ResU-Net++ (Jha et al., 2019) and AttU-Net (Wang et al., 2021).

The training process of four lung field segmentation models based on CNNs is detailed below. First, the 755 P-A CXR images’ lung field label images (ground truth) are labeled and examined by three experienced radiologists using the software Labelme (v5.1.0) and ITK-SNAP (v4.0.2). Second, each CNN is trained by 755 P-A CXR images (755 × 512 × 512 × 1) with their lung field label images (ground truth). Specifically, 755 CXR cases include 371 normal cases, 380 abnormal cases with the manifestation of tuberculosis (N = 320) and pneumonia (N = 60), and 15 unclear cases. In addition, data augmentation techniques were adopted to avoid overfitting, further improving the robustness and generalization ability of the lung field segmentation models in the training process (Chlap et al., 2021). The standard cross entropy loss function is selected to calculate the model’s loss and dynamically adjust each CNN’s parameters. Finally, the CD algorithm (Zhao et al., 2010) is applied to the lung field mask images generated by each CNN to eliminate non-lung field masks not connected to the lung field masks.

#### 2.2.2 Automatic CTR calculation

**FIGURE 3:**
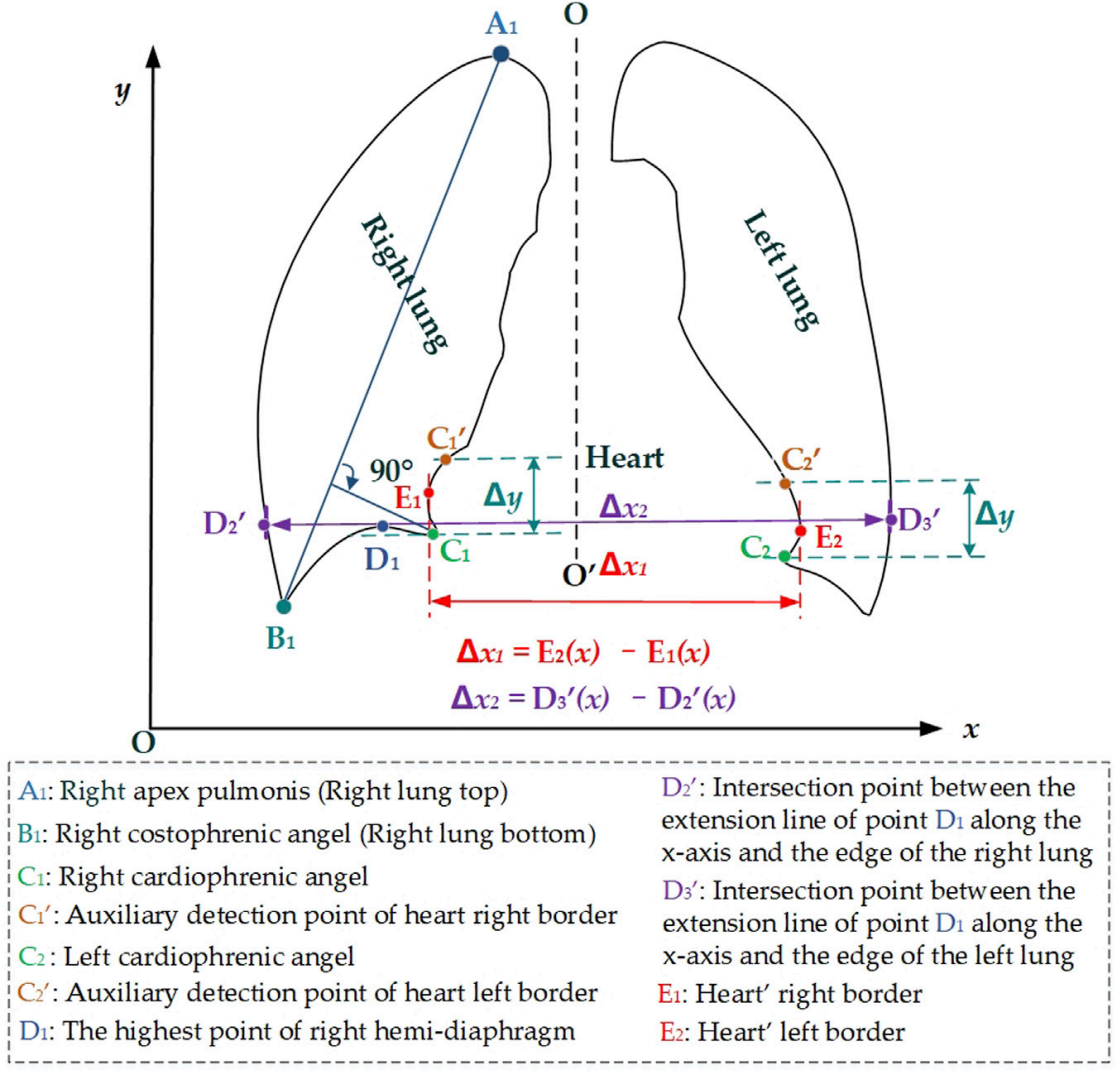
Schematic diagram of the automatic cardiothoracic ratio calculation is based on the images of the lung edge.

Figure 3 intuitively shows the schematic diagram of the automatic cardiothoracic ratio algorithm. First, the right and left lungs are identified based on lung field mask images. Specifically, the largest and the second largest lung field areas in each lung field mask image are identified as the right and left lung mask images, respectively. Second, edge detection is performed separately on left and right lung images, obtaining the right and left lung mask edge images. Finally, the right and left thoracic inner edge points ( $D_{2}^{\prime }$ and $D_{3}^{\prime }$ ) and right and left heart border points (*E*_*1*_ and *E*_*2*_) are located based on the images of the right and left lung mask edge for the CTR calculation. The source code is available on the website: https://github.com/YingjianYang/Automatic-Cardiothoracic-Ratio-Calculation.

Then, the right and left cardiophrenic angles *C*_*1*_ and *C*_*2*_ are configured as detection starting points for the right and left cardiac margins. Two straight lines are drawn perpendicular to the *y*-axis based on the *y*2 and *y*3 coordinates of C1 (x2, y2) and C2 (x3, y3). The intersection points $C_{1}^{\prime }$ and $C_{2}^{\prime }$ of these two straight lines with the right and left lung mask edge are configured as the termination points for the right and left cardiac margins. The heart’s right border *E*_*1*_ is located by calculating the maximum distance from all pixels on the right side along the right lung mask edge from the right cardiophrenic angles *C*_*1,*_ and the intersection points $C_{1}^{\prime }$ to the preset line O - $O^{\prime }$ . Meanwhile, the heart’s left border *E*_*2*_ is located by calculating the maximum distance from all pixels on the left side along the left lung mask edge from the left cardiophrenic angles *C*_*2*_ and the intersection points $C_{2}^{\prime }$ to the preset line O - $O^{\prime }$ . Furthermore, the above algorithm’s Equations 1–6 are provided.

$$O-O^{\prime }:a_{0}x+b_{0}y+c_{0}=0,$$ (1)

$$\begin{array}{l} E_{1}\left(x,y\right)\leftarrow \max\vec{D_{C_{1}-C_{1}^{\prime }}}=\max\left(d_{r1}\left(p_{r1}\right),d_{r2}\left(p_{r2}\right),d_{r3}\left(p_{r3}\right),...,d_{rn}\left(p_{rn}\right)\right)\\ =\max\left(d_{r1}\left(x_{r1},y_{r1}\right),d_{r2}\left(x_{r2},y_{r2}\right),d_{r3}\left(x_{r3},y_{r3}\right),...,d_{rn}\left(x_{rn},y_{rn}\right)\right)\\ =\max\left(\frac{\left|a_{0}x_{r1}+b_{0}y_{r1}+c_{0}\right|}{\sqrt{a_{0}^{2}+b_{0}^{2}}},\frac{\left|a_{0}x_{r2}+b_{0}y_{r2}+c_{0}\right|}{\sqrt{a_{0}^{2}+b_{0}^{2}}},\frac{\left|a_{0}x_{r3}+b_{0}y_{r3}+c_{0}\right|}{\sqrt{a_{0}^{2}+b_{0}^{2}}},...,\frac{\left|a_{0}x_{rn}+b_{0}y_{rn}+c_{0}\right|}{\sqrt{a_{0}^{2}+b_{0}^{2}}}\right) \end{array},$$ (2)

where the vector $\vec{D_{C_{1}-C_{1}^{\prime }}}=\left(d_{r1}\left(p_{r1}\right),d_{r2}\left(p_{r2}\right),d_{r3}\left(p_{r3}\right),...,d_{rn}\left(p_{rn}\right)\right)$ presents the Euclidean distances $d_{ri}$ of these coordinates $\left(p_{r1},p_{r2},p_{r3},...,p_{rn}\right)=\left(\left(x_{r1},y_{r1}\right),\left(x_{r2},y_{r2}\right),\left(x_{r3},y_{r3}\right),...,\left(x_{rn},y_{rn}\right)\right)$ to the preset line O *-* $O^{\prime }$ , and *i* = 1, 2, …, n. These coordinates $\left(x_{r1},y_{r1}\right),\left(x_{r2},y_{r2}\right),\left(x_{r3},y_{r3}\right),...,\left(x_{rn},y_{rn}\right)$ are extracted from all pixels in the left lung edge from the right cardiophrenic angles C_1_ and the intersection point $C_{1}^{\prime }$ on the left side of the preset line O *-* $O^{\prime }$ . The parameters $a_{0},b_{0},c_{0}$ are the coefficients of the preset line O *-* $O^{\prime }$ .

$$\begin{array}{l} E_{2}\left(x,y\right)\leftarrow \max\vec{D_{C_{2}-C_{2}^{\prime }}}=\max\left(d_{l1}\left(p_{l1}\right),d_{l2}\left(p_{l2}\right),d_{l3}\left(p_{l3}\right),...,d_{\ln}\left(p_{\ln}\right)\right)\\ =\max\left(d_{l1}\left(x_{l1},y_{l1}\right),d_{l2}\left(x_{l2},y_{l2}\right),d_{l3}\left(x_{l3},y_{l3}\right),...,d_{\ln}\left(x_{\ln},y_{\ln}\right)\right)\\ =\max\left.\left(\frac{\left|a_{0}x_{l1}+b_{0}y_{l1}+c_{0}\right|}{\sqrt{a_{0}^{2}+b_{0}^{2}}},\frac{\left|a_{0}x_{l2}+b_{0}y_{l2}+c_{0}\right|}{\sqrt{a_{0}^{2}+b_{0}^{2}}},\frac{\left|a_{0}x_{l3}+b_{0}y_{l3}+c_{0}\right|}{\sqrt{a_{0}^{2}+b_{0}^{2}}},...,\frac{\left|a_{0}x_{\ln}+b_{0}y_{\ln}+c_{0}\right|}{\sqrt{a_{0}^{2}+b_{0}^{2}}}\right)\right. \end{array},$$ (3)

where the vector $\vec{D_{C_{2}-C_{2}^{\prime }}}=\left(d_{l1}\left(p_{l1}\right),d_{l2}\left(p_{l2}\right),d_{l3}\left(p_{l3}\right),...,d_{\ln}\left(p_{\ln}\right)\right)$ presents the Euclidean distances $d_{li}$ of these coordinates $\left(p_{l1},p_{l2},p_{l3},...,p_{\ln}\right)=\left(\left(x_{l1},y_{l1}\right),\left(x_{l2},y_{l2}\right),\left(x_{l3},y_{l3}\right),...,\left(x_{\ln},y_{\ln}\right)\right)$ to the preset line O *-* $O^{\prime }$ , and *i* = 1, 2, …, n. These coordinates $\left(x_{l1},y_{l1}\right),\left(x_{l2},y_{l2}\right),\left(x_{l3},y_{l3}\right),...,\left(x_{\ln},y_{\ln}\right)$ are extracted from all pixels in the left lung edge from the left cardiophrenic angles C_2_ and the intersection point $C_{2}^{\prime }$ on the right side of the preset line O *-* $O^{\prime }$ .

In addition, the preset line O - $O^{\prime }$ is perpendicular to the *x*-axis (*b*_*1*_ = 0), so the formula (Liu et al., 2022) is converted to Equation 4. Based on Equation 4, Equations 2, 3 are further simplified to Equations 5, 6.

$$O-O^{\prime }:a_{o}x+c_{o}=0,$$ (4)

$$\begin{array}{l} E_{1}\left(x,y\right)\leftarrow \max\vec{D_{C_{1}-C_{1}^{\prime }}}=\max\left(d_{r1}\left(p_{r1}\right),d_{r2}\left(p_{r2}\right),d_{r3}\left(p_{r3}\right),...,d_{rn}\left(p_{rn}\right)\right)\\ =\max\left(d_{r1}\left(x_{r1},y_{r1}\right),d_{r2}\left(x_{r2},y_{r2}\right),d_{r3}\left(x_{r3},y_{r3}\right),...,d_{rn}\left(x_{rn},y_{rn}\right)\right)\\ =\max\left(\frac{\left|a_{o}x_{r1}+c_{o}\right|}{\sqrt{a_{o}^{2}}},\frac{\left|a_{o}x_{r2}+c_{o}\right|}{\sqrt{a_{o}^{2}}},\frac{\left|a_{o}x_{r3}+c_{o}\right|}{\sqrt{a_{o}^{2}}},...,\frac{\left|a_{o}x_{rn}+c_{o}\right|}{\sqrt{a_{o}^{2}}}\right)\\ =\max\left(\frac{\left|a_{o}x_{r1}+c_{o}\right|}{\left|a_{o}\right|},\frac{\left|a_{o}x_{r2}+c_{o}\right|}{\left|a_{o}\right|},\frac{\left|a_{o}x_{r3}+c_{o}\right|}{\left|a_{o}\right|},...,\frac{\left|a_{o}x_{rn}+c_{o}\right|}{\left|a_{o}\right|}\right) \end{array},$$ (5)

$$\begin{array}{l} E_{2}\left(x,y\right)\leftarrow \max\vec{D_{C_{2}-C_{2}^{\prime }}}=\max\left(d_{l1}\left(p_{l1}\right),d_{l2}\left(p_{l2}\right),d_{l3}\left(p_{l3}\right),...,d_{\ln}\left(p_{\ln}\right)\right)\\ =\max\left(d_{l1}\left(x_{l1},y_{l1}\right),d_{l2}\left(x_{l2},y_{l2}\right),d_{l3}\left(x_{l3},y_{l3}\right),...,d_{\ln}\left(x_{\ln},y_{\ln}\right)\right)\\ =\max\left(\frac{\left|a_{o}x_{l1}+c_{o}\right|}{\sqrt{a_{o}^{2}}},\frac{\left|a_{o}x_{l2}+c_{o}\right|}{\sqrt{a_{o}^{2}}},\frac{\left|a_{o}x_{l3}+c_{o}\right|}{\sqrt{a_{o}^{2}}},...,\frac{\left|a_{o}x_{\ln}+c_{o}\right|}{\sqrt{a_{o}^{2}}}\right)\\ =\max\left(\frac{\left|a_{o}x_{l1}+c_{o}\right|}{\left|a_{o}\right|},\frac{\left|a_{o}x_{l2}+c_{o}\right|}{\left|a_{o}\right|},\frac{\left|a_{o}x_{l3}+c_{o}\right|}{\left|a_{o}\right|},...,\frac{\left|a_{o}x_{\ln}+c_{o}\right|}{\left|a_{o}\right|}\right) \end{array}.$$ (6)

Finally, the distance between the right and left heart border points is configured as the maximum transverse diameter of the chest $\Delta x_{1}$ . In addition, the distance between the right thoracic inner edge point and the left thoracic inner edge point $D_{3}^{\prime }$ is configured as the maximum transverse diameter of the heart $\Delta x_{2}$ . Subsequently, the ratio of the heart’s maximum transverse diameter and the chest’s maximum transverse diameter $\Delta x_{2}$ was configured as the CTR calculation. Furthermore, the above algorithm’s Equations 7–9 are rovided.

$$\begin{array}{l} \Delta x_{1}=\left|x_{E_{1}}-x_{E_{2}}\right|=\left|x_{\max\vec{D_{C_{1}-C_{1}^{\prime }}}}-x_{\max\vec{D_{C_{2}-C_{2}^{\prime }}}}\right|\\ =\left|x_{\max\left(d_{r1}\left(p_{r1}\right),d_{r2}\left(p_{r2}\right),d_{r3}\left(p_{r3}\right),...,d_{rn}\left(p_{rn}\right)\right)}-x_{\max\left(d_{l1}\left(p_{l1}\right),d_{l2}\left(p_{l2}\right),d_{l3}\left(p_{l3}\right),...,d_{\ln}\left(p_{\ln}\right)\right)}\right|\\ =\left|x_{\max\left(d_{r1}\left(x_{r1},y_{r1}\right),d_{r2}\left(x_{r2},y_{r2}\right),d_{r3}\left(x_{r3},y_{r3}\right),...,d_{rn}\left(x_{rn},y_{rn}\right)\right)}\right.\\ \left.-x_{\max\left(d_{l1}\left(x_{l1},y_{l1}\right),d_{l2}\left(x_{l2},y_{l2}\right),d_{l3}\left(x_{l3},y_{l3}\right),...,d_{\ln}\left(x_{\ln},y_{\ln}\right)\right)}\right|\\ =\left|x_{\max\left(\frac{\left|a_{1}x_{r1}+c_{1}\right|}{\sqrt{a_{1}^{2}}},\frac{\left|a_{1}x_{r2}+c_{1}\right|}{\sqrt{a_{1}^{2}}},\frac{\left|a_{1}x_{r3}+c_{1}\right|}{\sqrt{a_{1}^{2}}},...,\frac{\left|a_{1}x_{rn}+c_{1}\right|}{\sqrt{a_{1}^{2}}}\right)}-x_{\max\left(\frac{\left|a_{1}x_{l1}+c_{1}\right|}{\sqrt{a_{1}^{2}}},\frac{\left|a_{1}x_{l2}+c_{1}\right|}{\sqrt{a_{1}^{2}}},\frac{\left|a_{1}x_{l3}+c_{1}\right|}{\sqrt{a_{1}^{2}}},...,\frac{\left|a_{1}x_{\ln}+c_{1}\right|}{\sqrt{a_{1}^{2}}}\right)}\right|\\ =\left|x_{\max\left(\frac{\left|a_{1}x_{r1}+c_{1}\right|}{\left|a_{1}\right|},\frac{\left|a_{1}x_{r2}+c_{1}\right|}{\left|a_{1}\right|},\frac{\left|a_{1}x_{r3}+c_{1}\right|}{\left|a_{1}\right|},...,\frac{\left|a_{1}x_{rn}+c_{1}\right|}{\left|a_{1}\right|}\right)}-x_{\max\left(\frac{\left|a_{1}x_{l1}+c_{1}\right|}{\left|a_{1}\right|},\frac{\left|a_{1}x_{l2}+c_{1}\right|}{\left|a_{1}\right|},\frac{\left|a_{1}x_{l3}+c_{1}\right|}{\left|a_{1}\right|},...,\frac{\left|a_{1}x_{\ln}+c_{1}\right|}{\left|a_{1}\right|}\right)}\right| \end{array},$$ (7)

$$\Delta x_{2}=\left|x_{D_{2}^{\prime }}-x_{D_{3}^{\prime }}\right|,$$ (8)

$$CTR=\frac{\Delta x_{1}}{\Delta x_{2}},$$ (9)

where $x_{E_{1}}$ and $x_{E_{2}}$ represent the horizontal coordinates *x(E*_*1*_ *)* and *x(E*_*2*_ *)* of the right and left heart border points *E*_*1*_ and *E*_*2*_, respectively. Similarly, $x_{D_{2}^{\prime }}$ and $x_{D_{3}^{\prime }}$ represent the horizontal coordinates *x(* $D_{2}^{\prime }$ *)* and *x(* $D_{3}^{\prime }$ *)* of the right and left thoracic inner edge points $D_{2}^{\prime }$ and $D_{3}^{\prime }$ , respectively.

#### 2.2.3 Experimental environment and evaluation metrics

These four traditional and basic CNNs (SegNet, U-Net, ResU-Net++, and AttU-Net) are trained on PyCharm 2017.3.3 (community edition) in Windows 10 Pro 64-bit with an NVIDIA GeForce GTX 1080 Ti GPU and 16 GB RAM. Then, the pth format of each CNN’s optimal lung field segmentation model is converted to the pth format based on PyCharm 2017.3.3. Finally, each CNN’s optimal lung field segmentation model with the pt format is called by C++ codes based on Visual Studio 2017 for lung field segmentation of 21 static P-A CXR images (Test set T1) and 13 dynamic P-A CXR images (Test set T2). Similarly, the CTR algorithm is automatically performed in Visual Studio 2017.

This study selects the six standard evaluation metrics of each lung field segmentation model, including accuracy, precision, recall, dice, intersection over union (IoU), and the median 95th Hausdorff distance (HD) (Yang et al., 2023; Zeng et al., 2023). Four *x*-axis direction distance errors are calculated to evaluate detected points between the right and left heart border points and thoracic inner edge points and their ground truths. Furthermore, the errors of calculated CTR and its ground truth were also calculated to evaluate the proposed method. Specifically, the evaluation metrics of the *x*-axis direction distance and CTR errors are calculated by Equations 10, 11.

$$x_{error}=x_{\text{DP}}-x_{GT},$$ (10)

$$CTR_{error}=CTR_{C}-CTR_{GT},$$ (11)

where $x_{\text{DP}}$ represents the horizontal coordinates *x(E*_*1*_ *), x(E*_*2*_ *), x(* $D_{2}^{\prime }$ *)*, and *x(* $D_{3}^{\prime }$ *)* of the detection points of *E*_*1*_ *, E*_*2*_ *,* $D_{2}^{\prime }$ , and $D_{3}^{\prime }$ , respectively. In addition, $x_{GT}$ represents the ground truth of the horizontal coordinates *x(E*_*1*_ *), x(E*_*2*_ *), x(* $D_{2}^{\prime }$ *)*, and *x(* $D_{3}^{\prime }$ *)*. $CTR_{C}$ and $CTR_{GT}$ represent the calculated value and ground truth of the CTR.

## 3 Results

**FIGURE 4:**
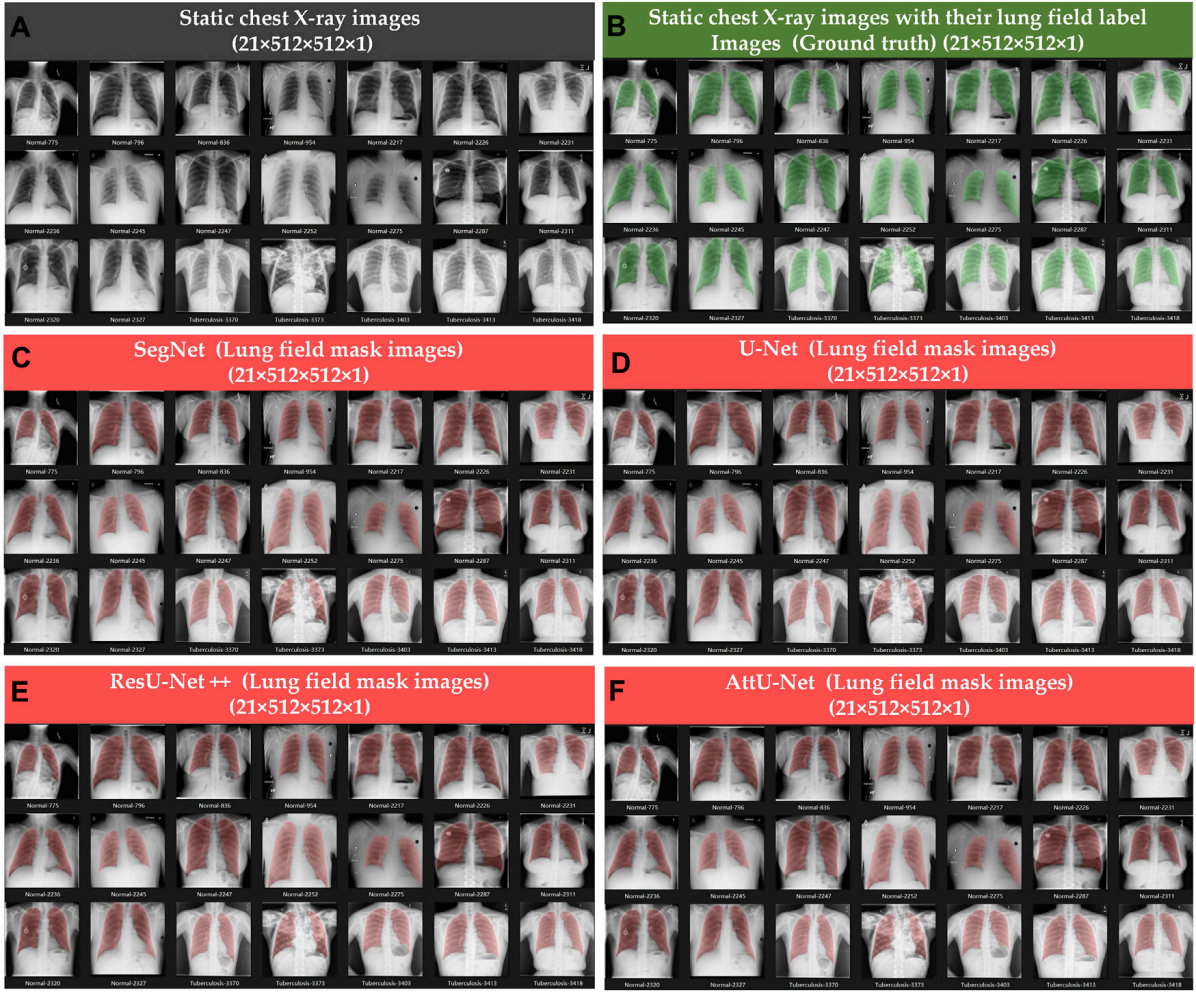
Visualized lung field segmentation results of the test set T1 based on various trained CNN models. **(A)** Twenty-one static P-A CXR images; **(B)** twenty-one static P-A CXR images with their lung field label images (ground truth); **(C)** twenty-one static lung field mask images based on SegNet; **(D)** twenty-one static lung field mask images based on U-Net; **(E)** twenty-one static lung field mask images based on ResU-Net++; and **(F)** twenty-one static lung field mask images based on AttU-Net.

**FIGURE 5:**
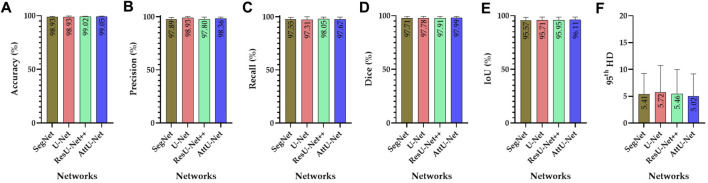
Visualized evaluation metrics of various trained CNN models on the test set T1. **(A)** Accuracy; **(B)** precision; **(C)** recall; **(D)** dice; **(E)** IoU; and **(F)** 95th HD.

**FIGURE 6:**
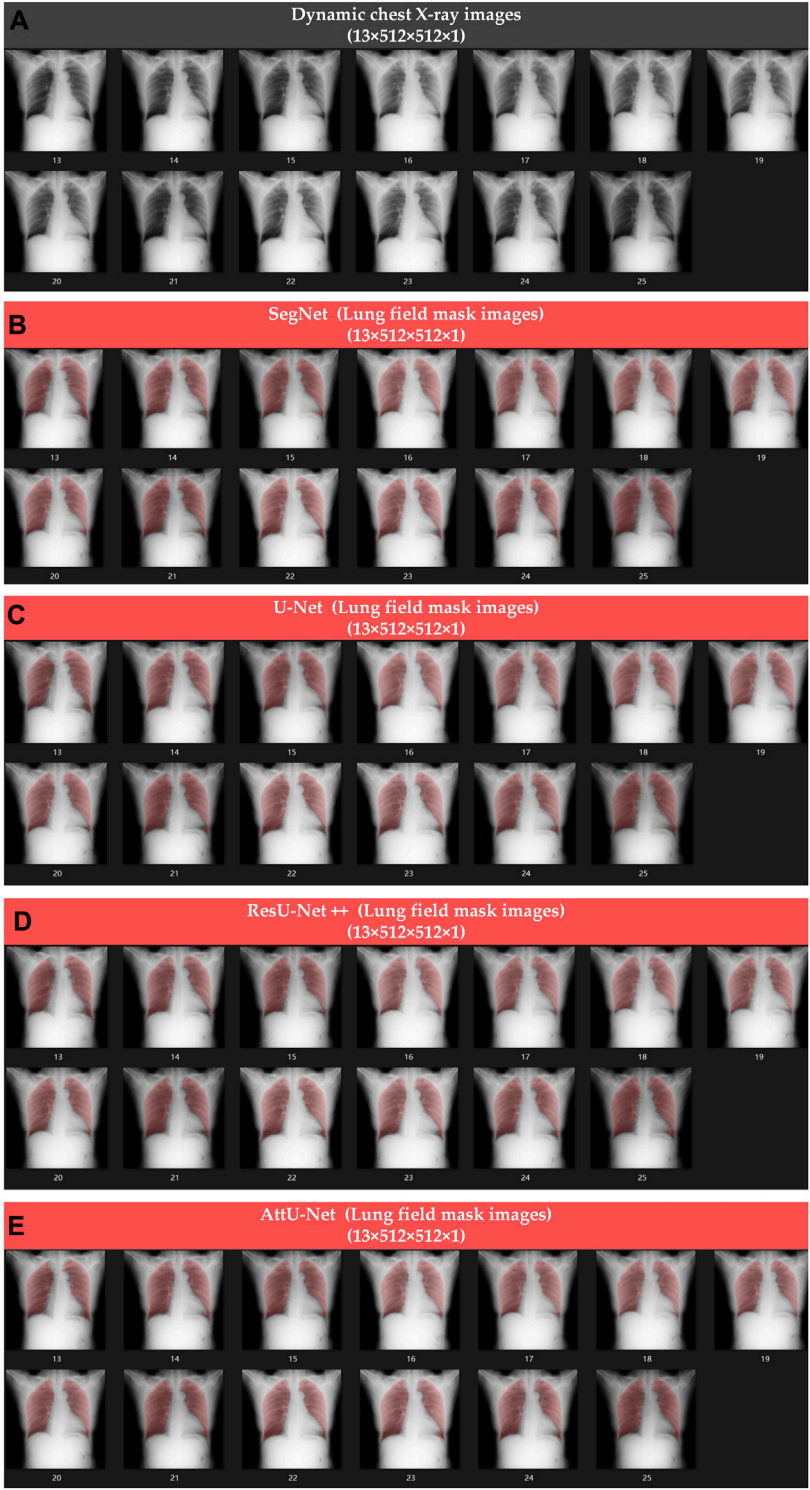
Visualized lung field segmentation results of the test set T2 based on various trained CNN models. **(A)** Thirteen dynamic P-A CXR images; **(B)** thirteen dynamic lung field mask images based on SegNet; **(C)** thirteen dynamic lung field mask images based on U-Net; **(D)** thirteen dynamic lung field mask images based on ResU-Net++; and **(E)** thirteen dynamic lung field mask images based on AttU-Net.

Figures 4, 5 show the visualized lung field segmentation results and evaluation metrics of the test set T1 based on various trained CNN models. In addition, Figure 6 shows that the visualized lung field segmentation results of the test set T2 are based on these trained CNN models. These results indicate that all CNNs can perform well in lung field segmentation for static and dynamic P-A CXR images.

Specifically, the mean accuracies (%) of these trained CNN models are 98.93 ± 0.63, 98.93 ± 0.85, 99.02 ± 0.60, and 99.05 ± 0.69, respectively. In addition, the mean precision (%) of these trained CNN models is 97.89 ± 1.49, 93.30 ± 1.40, 97.80 ± 1.96, and 98.36 ± 1.44, respectively. The mean recall (%) of these trained CNN models is 97.55 ± 1.93, 97.31 ± 2.70, 98.05 ± 1.69, and 97.67 ± 2.14, respectively. The mean dice (%) of these trained CNN models is 97.71 ± 1.56, 97.78 ± 1.63, 97.91 ± 1.46, and 97.99 ± 1.43, respectively. The mean IoU (%) of these trained CNN models is 95.57 ± 2.91, 95.71 ± 3.05, 95.95 ± 2.74, and 96.11 ± 2.69, respectively. Finally, the mean 95th HD of these trained CNN models is 5.41 ± 3.81, 5.72 ± 5.02, 5.46 ± 4.51, and 5.02 ± 4.15, respectively.

**FIGURE 7:**
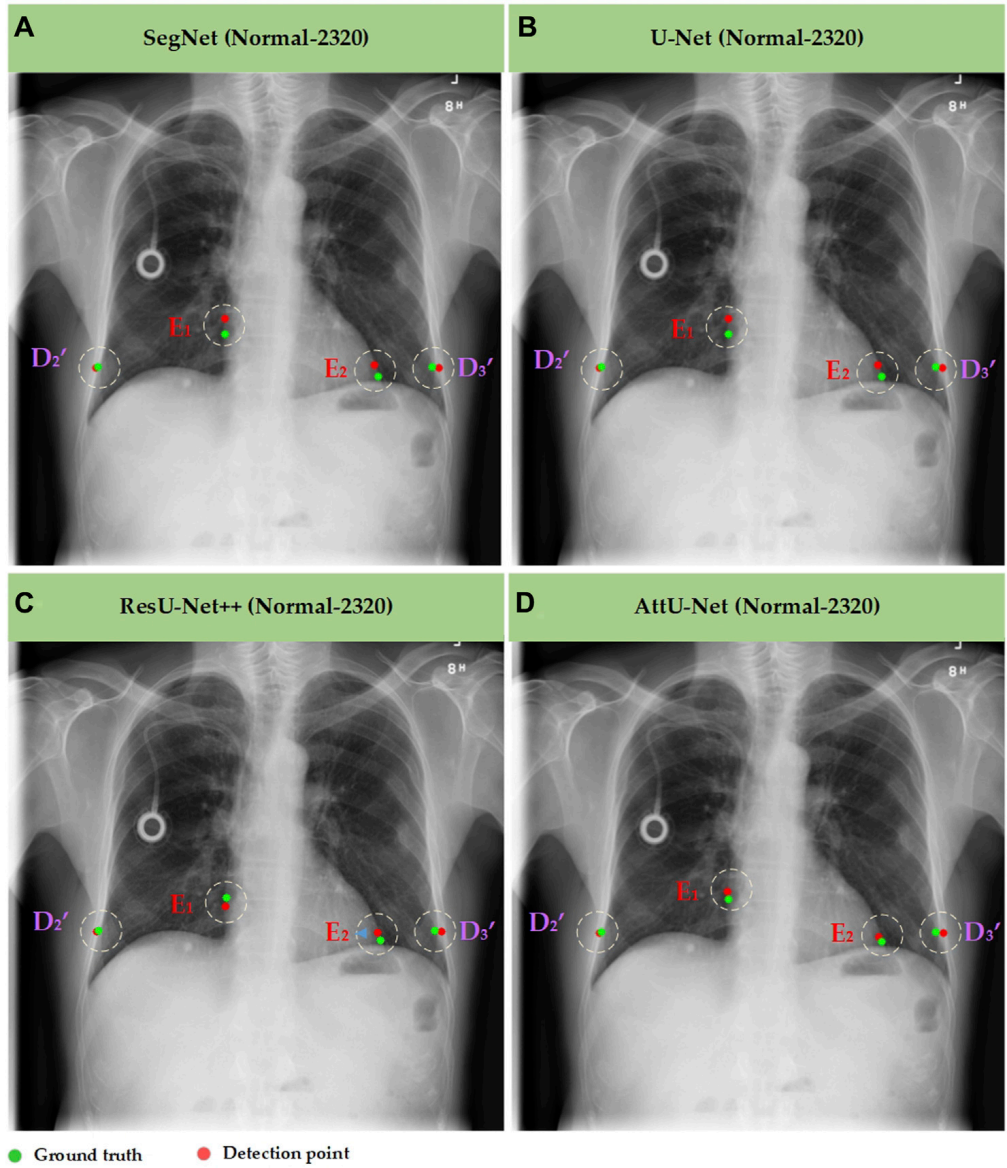
Typical key point detection results of a normal case in the test set T1 based on various trained CNN models. **(A)** SegNet; **(B)** U-Net; **(C)** ResU-Net++; and **(D)** AttU-Net.

**FIGURE 8:**
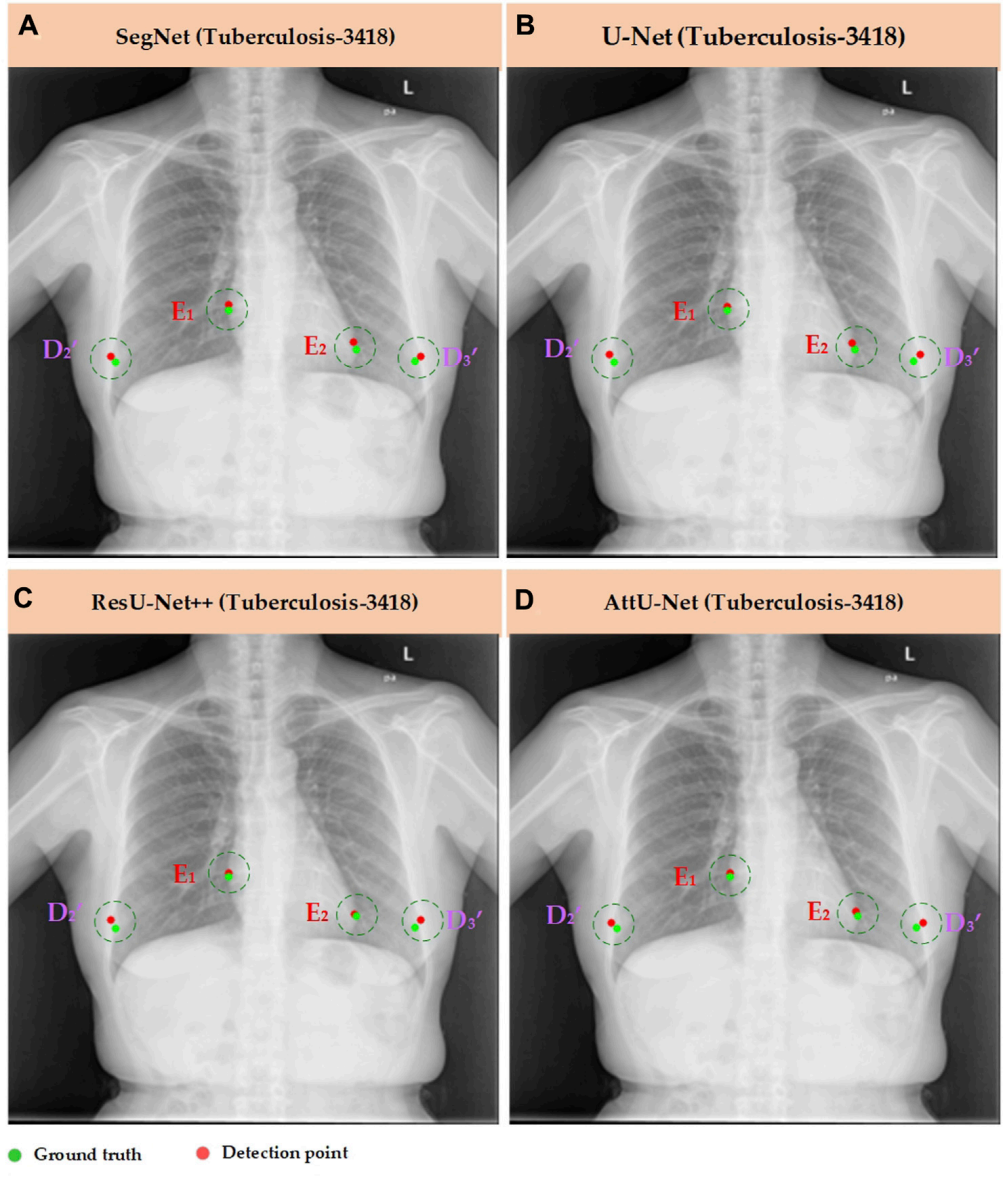
Typical key point detection results of a tuberculosis case in the test set T1 based on various trained CNN models. **(A)** SegNet; **(B)** U-Net; **(C)** ResU-Net++; and **(D)** AttU-Net.

**FIGURE 9:**
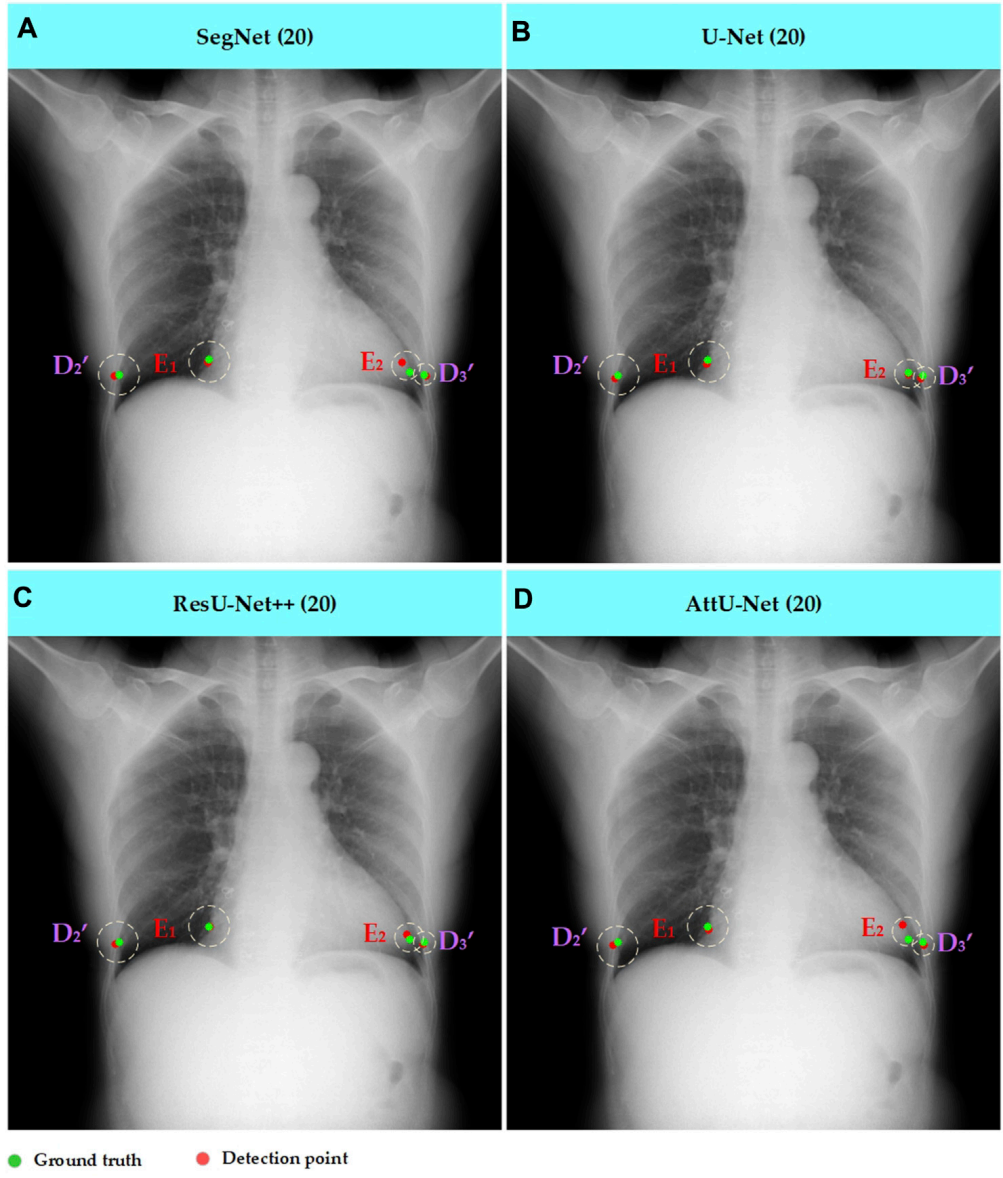
Typical key point detection results of a dynamic case in the test set T1 based on various trained CNN models. **(A)** SegNet; **(B)** U-Net; **(C)** ResU-Net++; and **(D)** AttU-Net.

**FIGURE 10:**
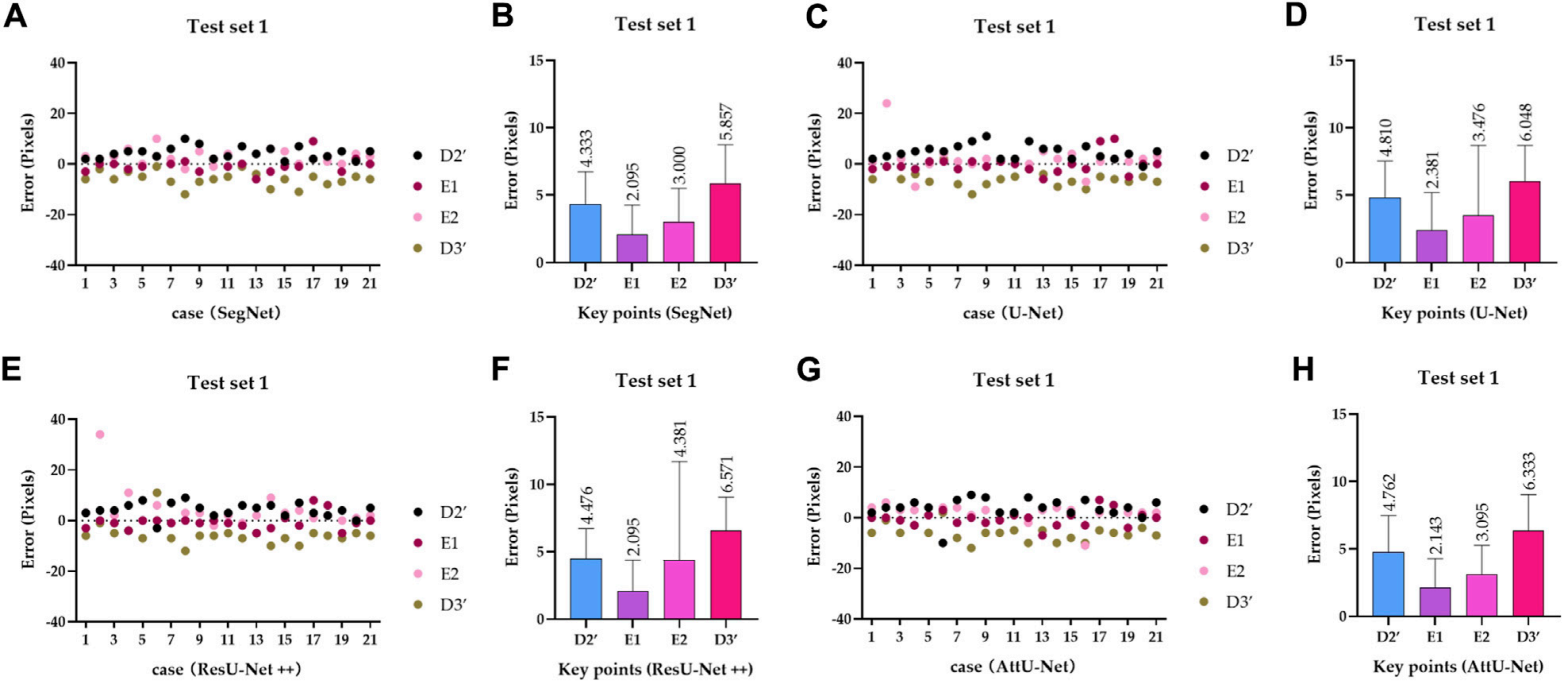
Visualized mean distance errors at the *x*-axis direction of key points on the test set T1. **(A)** Distribution map of key points’ distance errors based on SegNet; **(B)** mean with an SD plot of key points’ distance errors based on SegNet; **(C)** distribution map of key points’ distance errors based on U-Net; **(D)** mean with an SD plot of key points’ distance errors based on U-Net; **(E)** distribution map of key points’ distance errors based on ResU-Net++; **(F)** mean with an SD plot of key points’ distance errors based on ResU-Net++; **(G)** distribution map of key points’ distance errors based on AttU-Net; and **(H)** mean with an SD plot of key points’ distance errors based on AttU-Net.

Meanwhile, Figures 7–10 show the typical key point detection results of a tuberculosis case, a normal case in test set T1, and a dynamic case in test set T2 based on various trained CNN models. Specifically, the typical key points include the right and left thoracic inner edge points ( $D_{2}^{\prime }$ and $D_{3}^{\prime }$ ) and heart border points (E_1_ and E_2_).

**FIGURE 11:**
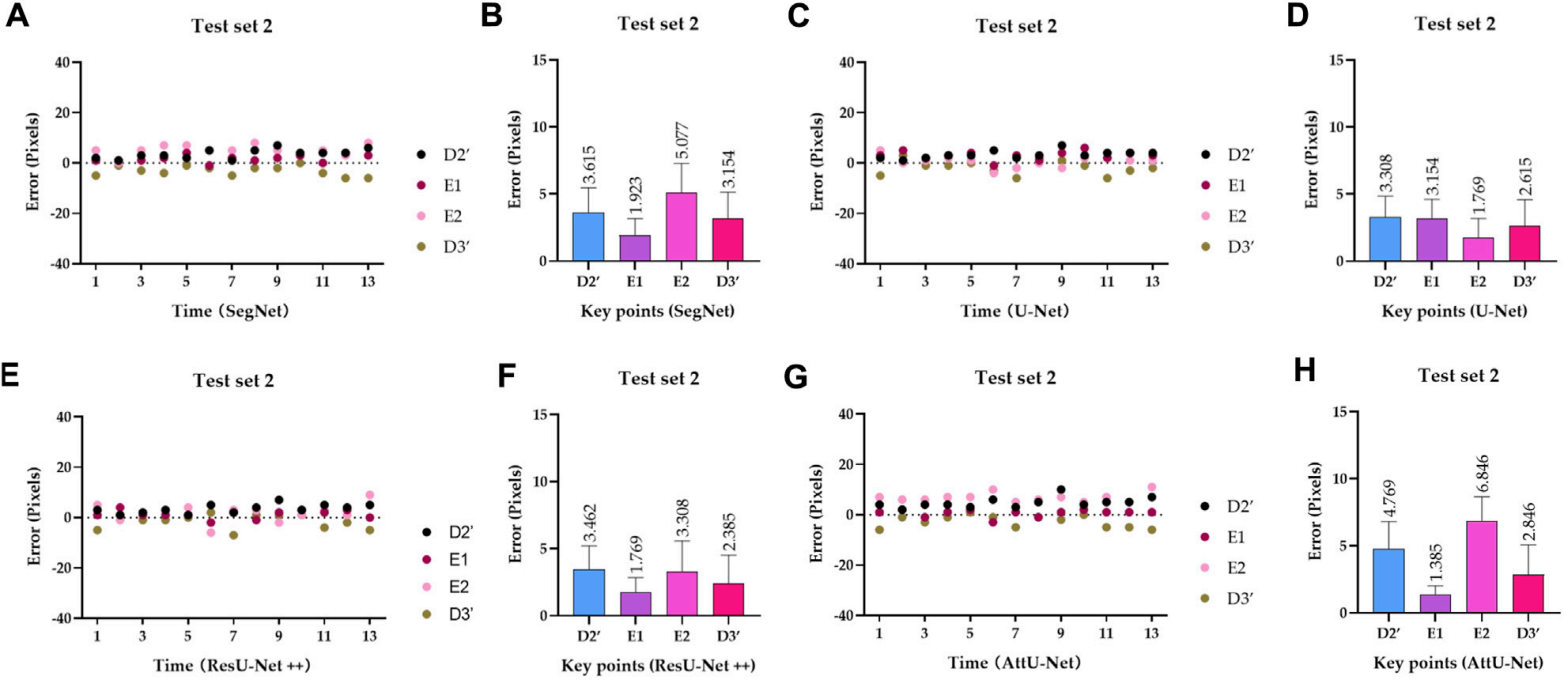
Visualized mean distance errors at the *x*-axis direction of key points on the test set T2. **(A)** Distribution map of key points’ distance errors based on SegNet; **(B)** mean with an SD plot of key points’ distance errors based on SegNet; **(C)** distribution map of key points’ distance errors based on U-Net; **(D)** mean with an SD plot of key points’ distance errors based on U-Net; **(E)** distribution map of key points’ distance errors based on ResU-Net++; **(F)** mean with an SD plot of key points’ distance errors based on ResU-Net++; **(G)** distribution map of key points’ distance errors based on AttU-Net; and **(H)** mean with an SD plot of key points’ distance errors based on AttU-Net.

To quantitatively evaluate the key points detection results of Figures 7–9, Table 1 reports the mean distance errors at the *x*-axis direction of key points in the test sets T1 and T2 based on various trained CNN models. In addition, Figures 10, 11 show the visualized mean distance errors at the *x*-axis direction of key points on the test set T1. The mean with SD plots of Figures 10, 11 is drawn based on the absolute values of these distance errors at the *x*-axis direction of key points.

**TABLE 1:** Mean distance errors at the *x*-axis direction of key points in the sets T1 and T2 based on various trained CNN models.

Network/test set	Mean error of point D_2_’ (pixels)	Mean error of point D_3_’ (pixels)	Mean error of point E_1_ (pixels)	Mean error of point E_2_ (pixels)	Mean (pixels)	
SegNet/T1	4.333	5.857	2.095	3.000	3.8213	4.1161
U-Net/T1	4.810	6.048	2.381	3.476	4.1788	
ResU-Net++/T1	4.476	6.571	2.095	4.381	4.3808	
AttU-Net/T1	4.762	6.333	2.143	3.095	4.0833	
SegNet/T2	3.615	3.154	1.923	5.077	3.4423	3.2116
U-Net/T2	3.308	2.615	3.154	1.769	2.7115	
ResU-Net++/T2	3.462	2.385	1.769	3.308	2.7310	
AttU-Net/T2	4.769	2.846	1.385	6.846	3.9615	

Specifically, mean distance errors at the *x*-axis direction of key points in the test set T1 based on the four trained CNN models are 3.8213 pixels, 4.1788 pixels, 4.3808 pixels, and 4.0833 pixels, respectively. Meanwhile, mean distance errors at the *x*-axis direction of key points in the test set T2 based on the four trained CNN models are 3.4423 pixels, 2.7115 pixels, 2.7310 pixels, and 3.9615 pixels, respectively. Mean distance errors at the *x*-axis direction of key points in the sets T1 and T2 of all trained CNN models are 4.1161 pixels and 3.2116 pixels, respectively. The mean distance error at the *x*-axis direction of key points in the test sets T1 and T2 based on various trained CNN models is approximately 3.6639 [≈(4.1161 + 3.2116)/2] pixels. Therefore, the deviation degree at the *x*-axis direction is about 0.72% (3.6639/512) on all 512 × 512 P-A CXR images.

**FIGURE 12:**
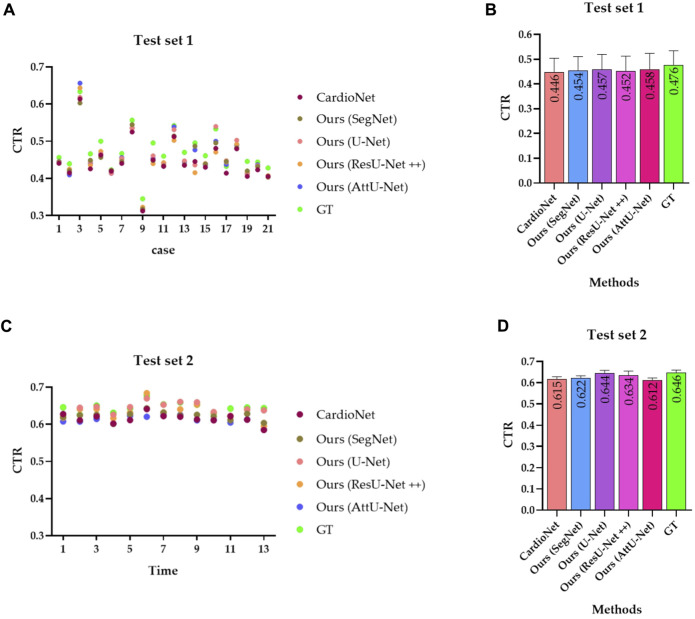
Visualized CTR of the test sets T1 and T2 based on various models. **(A)** CTR distribution map of the test set T1; **(B)** mean with an SD plot of CTR on test set T1; **(C)** CTR distribution map of the test set T2; and **(D)** mean with an SD plot of CTR on test set T2.

Subsequently, Table 2 compares the mean CTR error in the test sets T1 and T2 based on the previous methods (CardioNet) (Jafar et al., 2022) and our proposed models. In addition, Figure 12 shows the visualized CTR of the test sets T1 and T2 based on various models.

**TABLE 2:** Comparison of the mean CTR error in the test sets T1 and T2 based on previous and our proposed models.

Model	Mean CTR/T1	Mean CTR/T2	Mean T1 error	Mean T2 error	Mean error
CardioNet Jafar et al. (2022)	0.446	0.615	0.030	0.031	0.0305
Ours (SegNet)	0.454	0.622	0.022	0.024	0.0230
Ours (U-Net)	0.457	0.644	0.019	0.002	0.0105
Ours (ResU-Net++)	0.452	0.634	0.024	0.012	0.0180
Ours (AttU-Net)	0.458	0.612	0.018	0.034	0.0260

Specifically, the mean CTR values in the test set T1 based on these models are 0.446, 0.454, 0.457, 0.452, and 0.458. In addition, the mean CTR values in the test set T2 based on these models are 0.615, 0.622, 0.644, 0.634, and 0.612. More specifically, the ground truths of CTR in the test sets T1 and T2 are determined by these three experienced radiologists. Mean CTR errors in the test set T1 based on these models are 0.030, 0.022, 0.019, 0.024, and 0.018. In addition, mean CTR errors in the test set T2 based on these models are 0.031, 0.024, 0.002, 0.012, and 0.034. Meanwhile, mean CTR errors in the test sets T1 and T2 based on these models are 0.0305 [(0.030 + 0.031/2)], 0.0230 [(0.022 + 0.024/2)], 0.0105 [(0.019 + 0.002/2)], 0.0180 [(0.024 + 0.012/2)], and 0.0260 [(0.018 + 0.034/2)]. Therefore, the experimental results show that our proposed model achieves the equivalent performance of CTR calculation as the previous CardioNet model.

Table 3 compares the mean segmentation time, mean CTR calculation time, and mean total time of the test sets T1 and T2 based on previous and our proposed models. Specifically, these trained CNN models run on the GPU for segmenting the lung field and/or heart, and then, the CTR calculation algorithm based on the lung field and/or heart mask images runs on the CPU. The previous CardioNet takes more mean segmentation time, mean CTR calculation time, and mean total time of the test sets T1 and T2 than our proposed model. However, when the GPU runs the segmentation task for the first CXR image, it requires an amount of time to configure and load the corresponding model. For example, it takes 4,897/4,885 ms (CardioNet),951/979 ms (SegNet), 2,249/2,350 ms (U-Net), 2,182/2,226 ms (ResU-Net++), and 2,144/2,158 ms (AttU-Net), when the GPU runs the segmentation task for the first CXR image of the test set T1/T2.

**TABLE 3:** Comparison of the mean segmentation time, mean CTR calculation time, and mean total time of the test sets T1 and T2 based on previous and our proposed models.

Model	Mean segmentation time of T1/T2 (ms)	Mean CTR calculation time of T1/T2 (ms)	Mean total time of T1/T2 (ms)
CardioNet Jafar et al. (2022)	347.126/368.638	14.238/13.071	361.364/381.709
Ours (SegNet)	58.333/91.769	7.000/6.923	65.333/98.692
Ours (U-Net)	123.333/196.077	8.048/6.231	131.381/202.308
Ours (ResU-Net++)	184.476/257.000	6.476/6.462	190.952/263.462
Ours (AttU-Net)	118.190/188.385	6.571/6.077	124.761/194.462

## 4 Discussion

This section conducts the following discussion and points out this study’s limitations and the future direction based on the experimental results.

### 4.1 Dynamic lung field segmentation driven by CNNs with static P-A CXR images

The CNN lung field segmentation model trained by inspiratory chest computed tomography (CT) images has been applied to the lung field segmentation of inspiratory and expiratory chest CT images, achieving good performance (Deng et al., 2024; Wang et al., 2024). Similarly, the lung field segmentation model trained on CNNs based on static P-A CXR images also demonstrated good performance in lung field segmentation of dynamic P-A CXR images. Therefore, this provides a necessary foundation for quantitative analysis of dynamic P-A CXR images, such as the CTR calculation. Specifically, CNNs have also played a crucial role in semantic segmentation, where the goal is to assign a class label to each pixel in an image, enabling pixel-level understanding and overcoming the limitations of the traditional approaches (Robert, 2024). In addition, a robust and standard segmentation model of pathological lungs is crucial for quantitative analysis of the lungs based on P-A CXR images. However, generalizing lung field segmentation models based on P-A CXR images has always been a significant engineering problem in clinical applications (Rajaraman et al., 2024). The main reason for this engineering problem is the lack of cross-center P-A CXR images and their diversity. The data augmentation technology enriches the training set of the static P-A CXR images and relieves the engineering problem of generalization in lung field segmentation models (Hasan et al., 2024; Kiruthika and Khilar, 2024). Therefore, the static P-A CXR images form a single center, limiting the generalization of lung field segmentation models. Meanwhile, the diversity of pathological static P-A CXR images in the training set is also essential for improving the generalization of lung field segmentation models, enabling CNNs to learn more prior knowledge. The static P-A CXR images of the cross-center and the diversity and data augmentation techniques may fundamentally solve the generalization problem of lung field segmentation models.

### 4.2 The lung field morphology for automatic and precise CTR calculation

The positional relationship between the lung field and the heart on the P-A CXR images is the basis for calculating the CTR based on the lung field. Specifically, the right and left cardiophrenic angles *C*_*1*_ and *C*_*2*_ are two relatively prominent points on the P-A CXR images after careful observation and analysis of the P-A CXR images. This provides a certain possibility for automatically calculating the CTR based on the P-A CXR images. Significantly, the right cardiophrenic angle *C*_*1*_ is the farthest point from the line connecting the right apex pulmonis *A*_*1*_ and costophrenic angle *B*_*1*_, which helps determine the highest point *D*_*1*_ on the right hemi-diaphragm from the costophrenic angle *B*_*1*_ to the right cardiophrenic angles *C*_*1*_. Meanwhile, the right and left heart border points *E*_*1*_ and *E*_*2*_ are closely adjacent to the left and right lung fields and form inward indentations on the opposite sides of the left and right lung fields on the P-A CXR images. Therefore, this facilitates the location of the right and left heart border points *E*_*1*_ and *E*_*2*_ on lung mask edges from the right and left cardiophrenic angles *C*_*1*_ and *C*_*2*_ to the intersection points $C_{1}^{\prime }$ and $C_{2}^{\prime }$ , respectively. Based on the above, the two-dimensional projection morphology of the lung field enables automatic and precise CTR calculation, overcoming the limitations to heart segmentation and avoiding errors in heart segmentation. In addition, our proposed models take less time to calculate the CTR, which benefits that the proposed models only segment the lung field compared with the previous CardioNet model. Our proposed model achieves the equivalent performance of CTR calculation as the previous CardioNet model (Jafar et al., 2022).

### 4.3 Providing the possibility for the analysis and evaluation of dynamic CTRs

Dynamic CTRs can directly reflect the relationship between the changes in the maximum transverse diameter of the chest during the respiratory process and the maximum transverse diameter of the heart at different cardiac cycles. Actually, the chest can be imaged by autonomously controlling breathing while performing a chest X-ray. However, the heartbeat process cannot be autonomously controlled while chest X-rays are performed. Therefore, the models developed above may provide evidence of the possibility of analyzing and evaluating dynamic CTRs. Specifically, the corresponding P-A CXR images of the different cardiac cycles can be obtained by controlling the breathing state at any moment during the breathing process, such as deep inhalation, deep exhalation, or breath holding. Subsequently, dynamic CRTs for different cardiac cycles can be calculated for clinical analysis and evaluation. Meanwhile, like inspiratory and expiratory chest CT images (Deng et al., 2024; Wang et al., 2024), P-A CXR images of the different cardiac cycles can be obtained by controlling the holding of breath to achieve deep inhalation and exhalation, to analyze the difference in CTR between deep inhalation and exhalation.

### 4.4 Limitations and future research directions

Although we propose an automated CTR calculation technique based on lung field models from an engineering perspective, our research still has certain limitations. First, the pathological lung image types used for training CNNs are insufficient. Second, we only explain the principle of automatically calculating the CTR based on the lung fields based on graphics and achieve the detection of dynamic CTRs. However, we do not have sufficient dynamic P-A chest X-ray images to further analyze the association between dynamic CTRs and specific lung or heart diseases from a clinical perspective, for example, differences in dynamic CTRs under different GOLD classifications in chronic pulmonary heart disease caused by chronic obstructive pulmonary disease. Based on the above, we encourage researchers to collect more dynamic P-A CXR images of different lung diseases to improve the lung field segmentation model further and hope to discover more clinically significant facts based on dynamic CTRs.

## 5 Conclusion

We propose an automatic CTR calculation model based on lung fields abstracted from P-A CXR images without heart segmentation. First, the lung field mask images are abstracted from the P-A CXR images based on the pre-trained CNNs. Second, a novel localization method of the heart’s right and left border points is proposed based on the two-dimensional projection morphology of the lung field mask images using graphics. The results show that the mean distance errors at the *x*-axis direction of the CTR’s four key points in the test sets T1 and T2 based on these pre-trained CNNs are 4.1161 and 3.2116 pixels, respectively. In addition, the mean CTR errors on the test set T1 and T2 based on four proposed models are 0.0208 and 0.0180, respectively. Our proposed model achieves the equivalent performance of CTR calculation as the previous CardioNet model and takes less time. Therefore, our proposed method is practical and feasible and may become an effective tool for initially evaluating cardiac diseases.

## Data Availability

The raw data supporting the conclusions of this article will be made available by the authors, without undue reservation.
