# Supplementary figures and images for: Automatic cardiothoracic ratio calculation based on lung fields abstracted from chest X-ray images without heart segmentation (part 2 of 2)
Source: Front Physiol. 2024 Aug 8;15:1416912. doi: 10.3389/fphys.2024.1416912 (PMC11338915; doi:10.3389/fphys.2024.1416912)

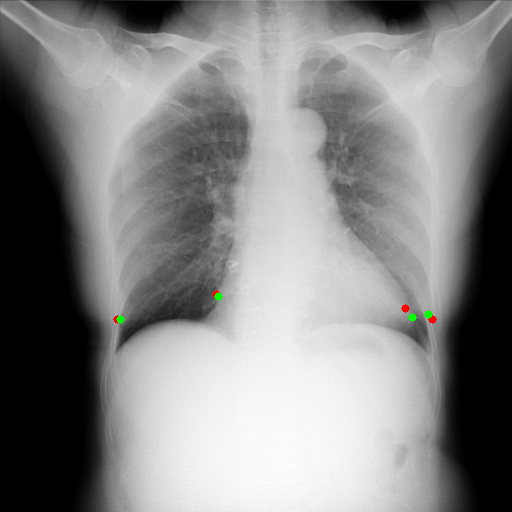

Supplement: Supplementary file 1 [file Presentation1.zip › Supplementary materials/s2/SegNet/16.png]

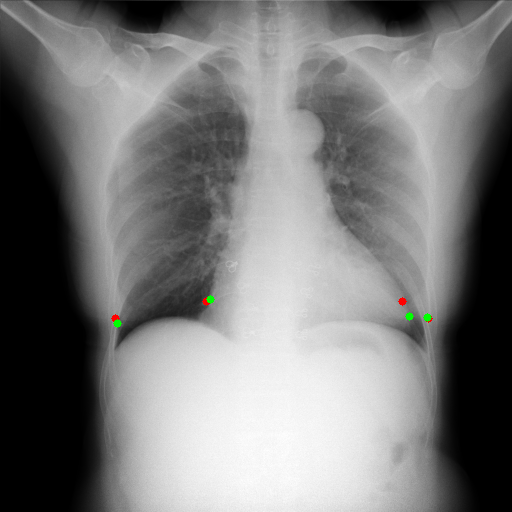

Supplement: Supplementary file 1 [file Presentation1.zip › Supplementary materials/s2/SegNet/17.png]

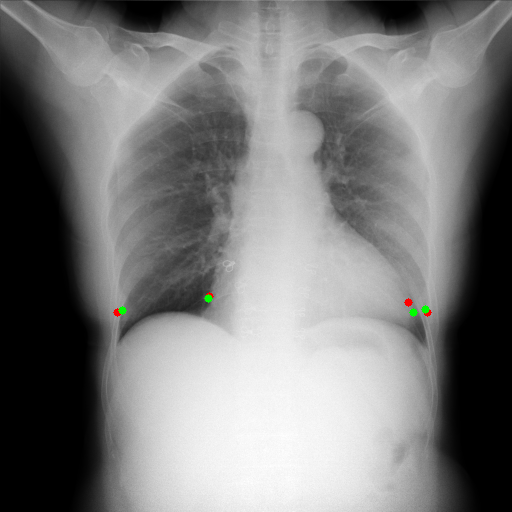

Supplement: Supplementary file 1 [file Presentation1.zip › Supplementary materials/s2/SegNet/18.png]

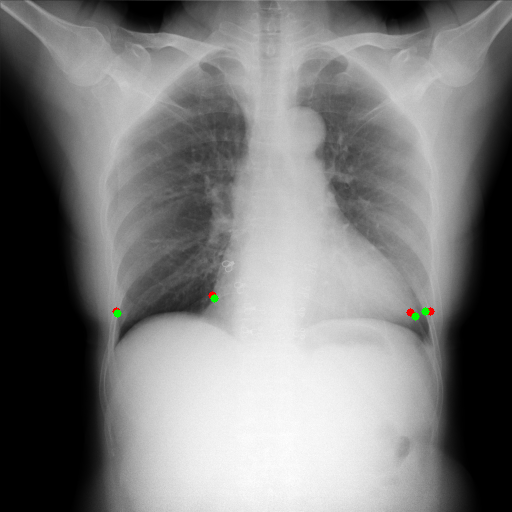

Supplement: Supplementary file 1 [file Presentation1.zip › Supplementary materials/s2/SegNet/19.png]

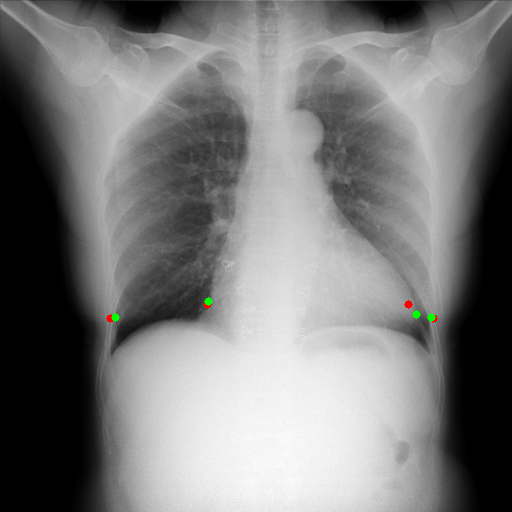

Supplement: Supplementary file 1 [file Presentation1.zip › Supplementary materials/s2/SegNet/20.png]

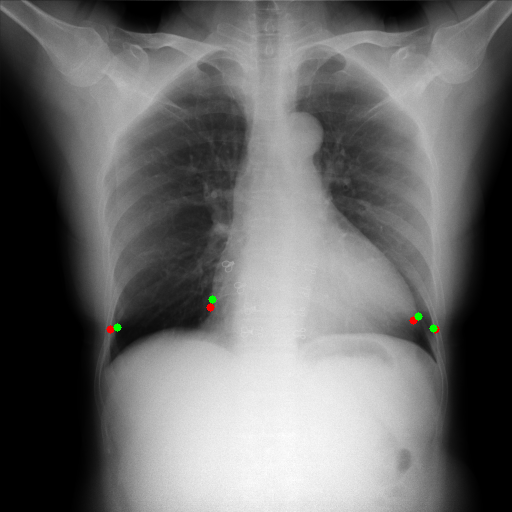

Supplement: Supplementary file 1 [file Presentation1.zip › Supplementary materials/s2/SegNet/21.png]

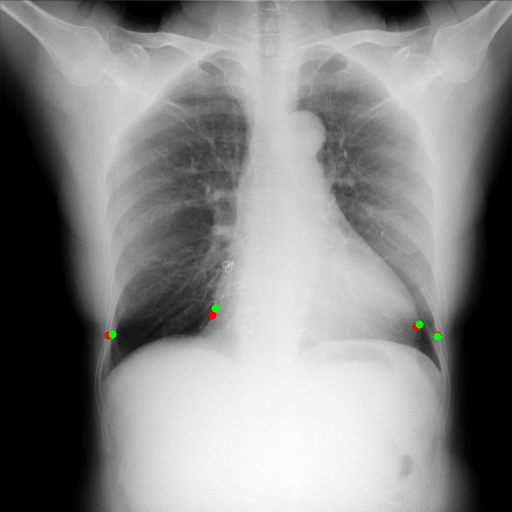

Supplement: Supplementary file 1 [file Presentation1.zip › Supplementary materials/s2/SegNet/22.png]

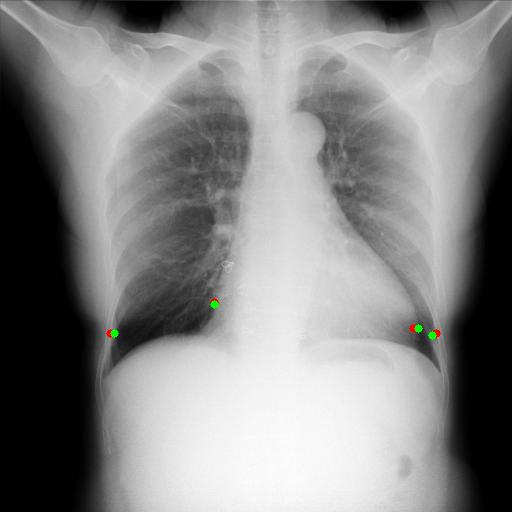

Supplement: Supplementary file 1 [file Presentation1.zip › Supplementary materials/s2/SegNet/23.png]

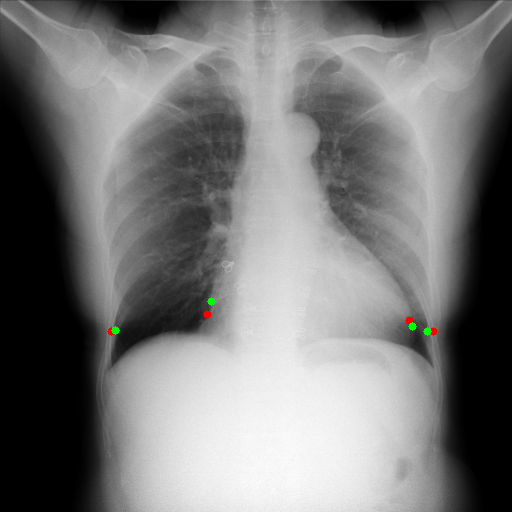

Supplement: Supplementary file 1 [file Presentation1.zip › Supplementary materials/s2/SegNet/24.png]

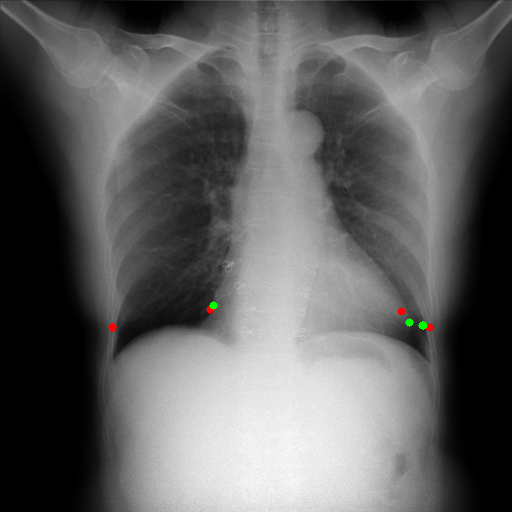

Supplement: Supplementary file 1 [file Presentation1.zip › Supplementary materials/s2/SegNet/25.png]

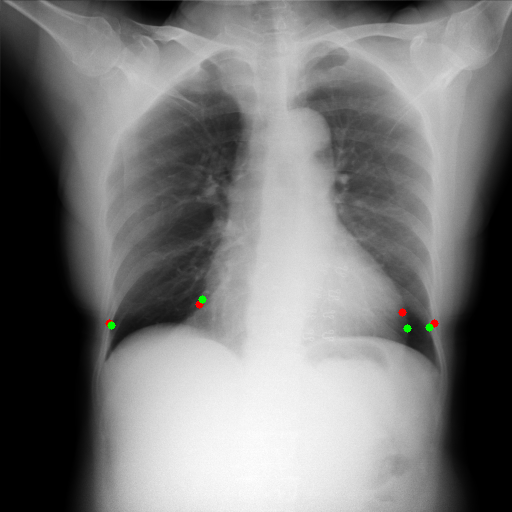

Supplement: Supplementary file 1 [file Presentation1.zip › Supplementary materials/s2/Unet/13.png]

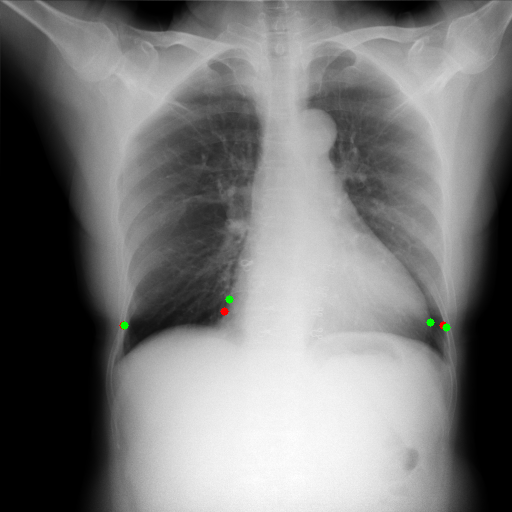

Supplement: Supplementary file 1 [file Presentation1.zip › Supplementary materials/s2/Unet/14.png]

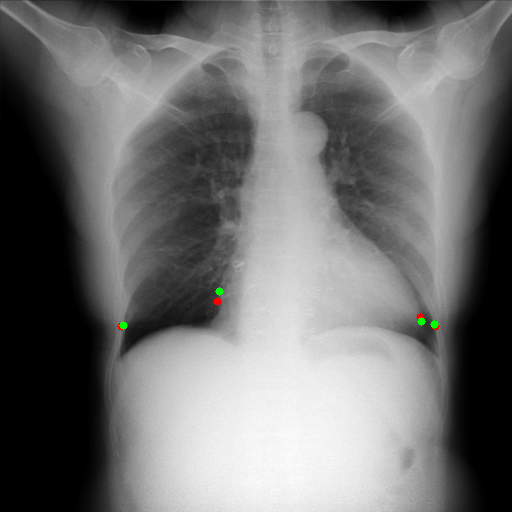

Supplement: Supplementary file 1 [file Presentation1.zip › Supplementary materials/s2/Unet/15.png]

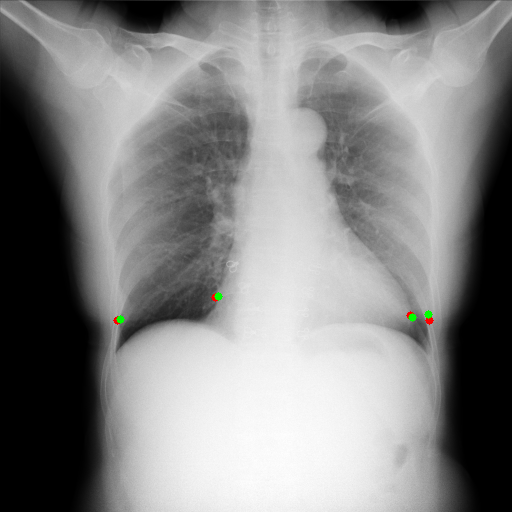

Supplement: Supplementary file 1 [file Presentation1.zip › Supplementary materials/s2/Unet/16.png]

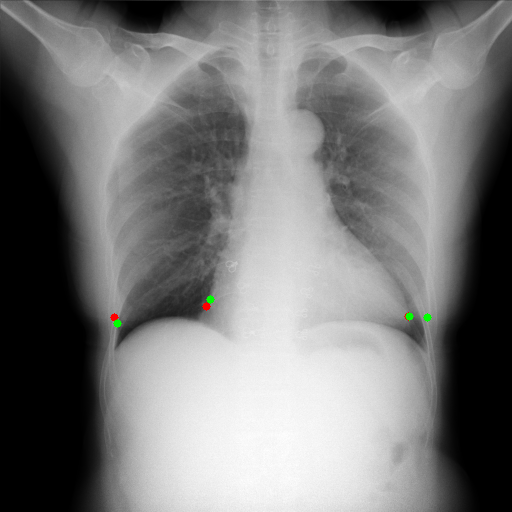

Supplement: Supplementary file 1 [file Presentation1.zip › Supplementary materials/s2/Unet/17.png]

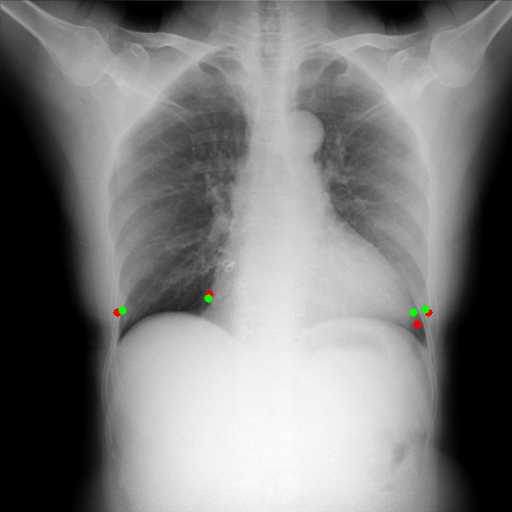

Supplement: Supplementary file 1 [file Presentation1.zip › Supplementary materials/s2/Unet/18.png]

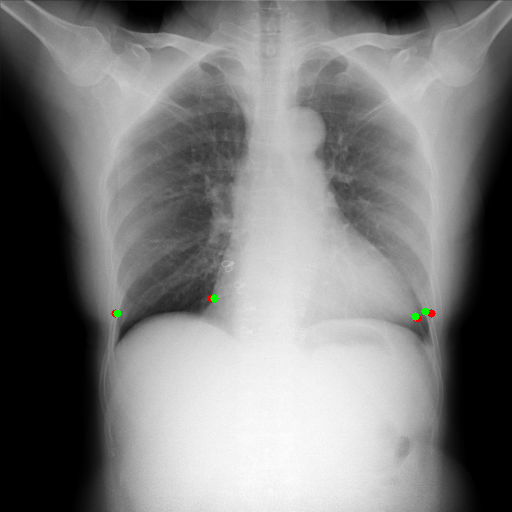

Supplement: Supplementary file 1 [file Presentation1.zip › Supplementary materials/s2/Unet/19.png]

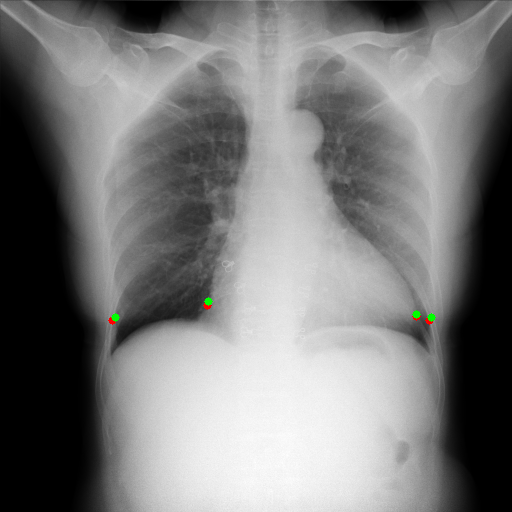

Supplement: Supplementary file 1 [file Presentation1.zip › Supplementary materials/s2/Unet/20.png]

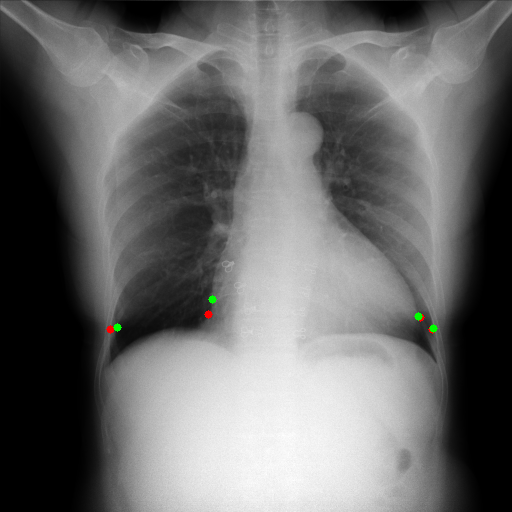

Supplement: Supplementary file 1 [file Presentation1.zip › Supplementary materials/s2/Unet/21.png]

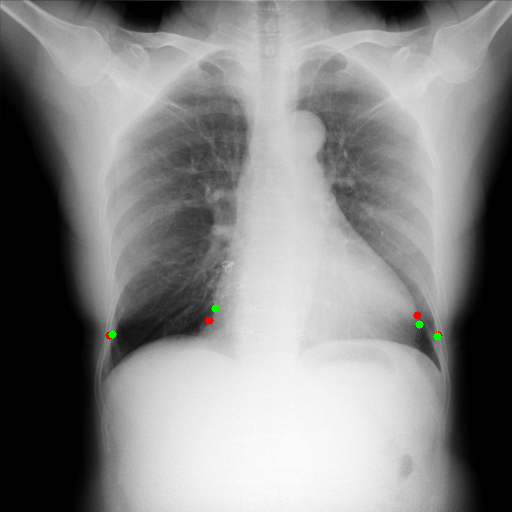

Supplement: Supplementary file 1 [file Presentation1.zip › Supplementary materials/s2/Unet/22.png]

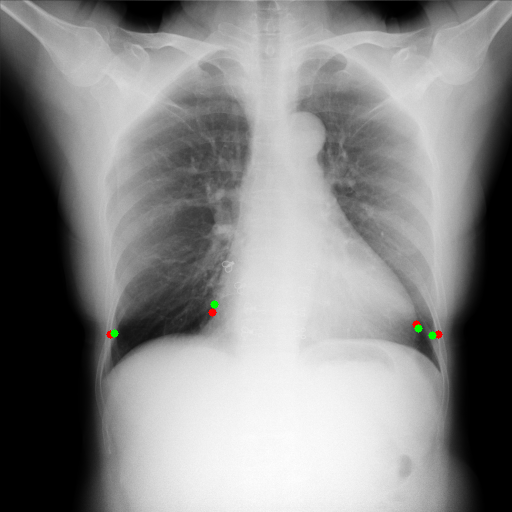

Supplement: Supplementary file 1 [file Presentation1.zip › Supplementary materials/s2/Unet/23.png]

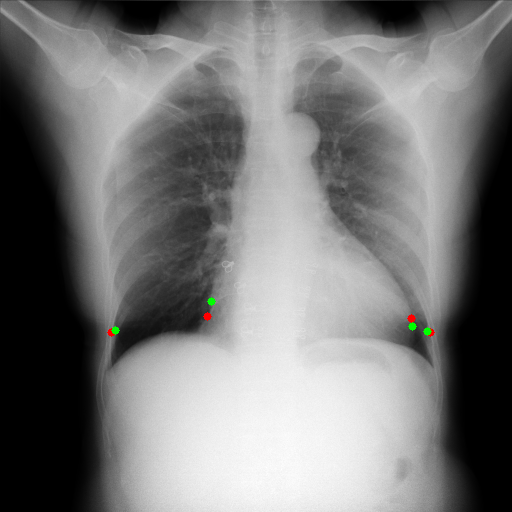

Supplement: Supplementary file 1 [file Presentation1.zip › Supplementary materials/s2/Unet/24.png]

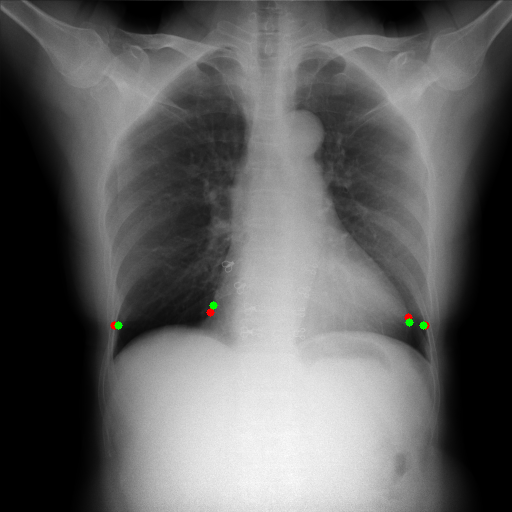

Supplement: Supplementary file 1 [file Presentation1.zip › Supplementary materials/s2/Unet/25.png]

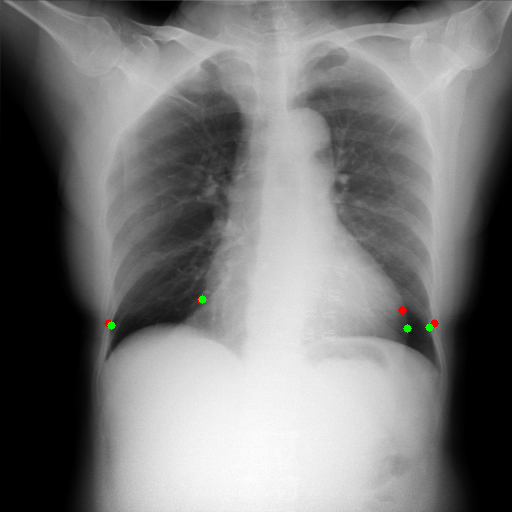

Supplement: Supplementary file 1 [file Presentation1.zip › Supplementary materials/s2/resUnet++/13.png]

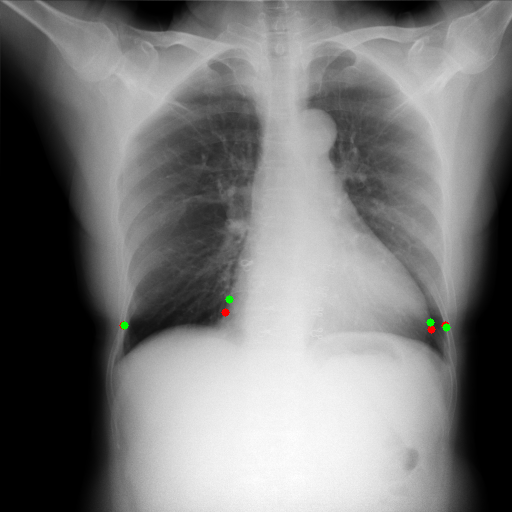

Supplement: Supplementary file 1 [file Presentation1.zip › Supplementary materials/s2/resUnet++/14.png]

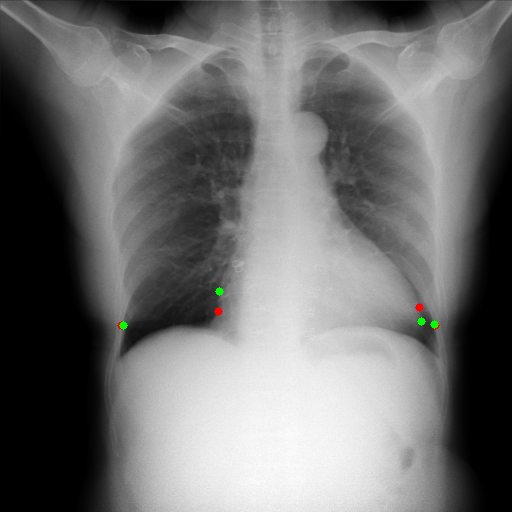

Supplement: Supplementary file 1 [file Presentation1.zip › Supplementary materials/s2/resUnet++/15.png]

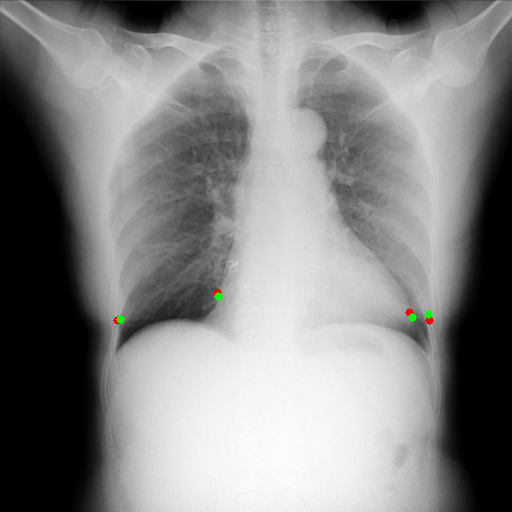

Supplement: Supplementary file 1 [file Presentation1.zip › Supplementary materials/s2/resUnet++/16.png]

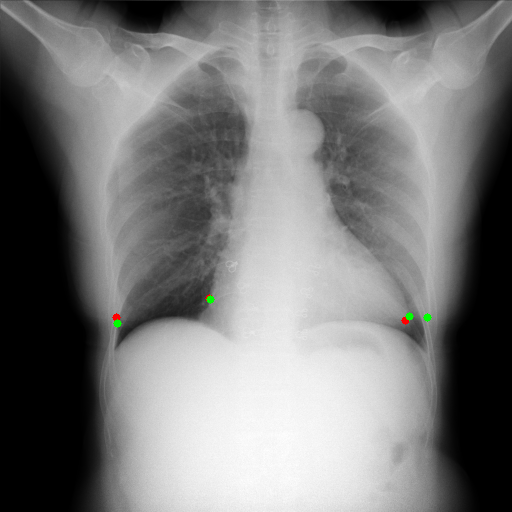

Supplement: Supplementary file 1 [file Presentation1.zip › Supplementary materials/s2/resUnet++/17.png]

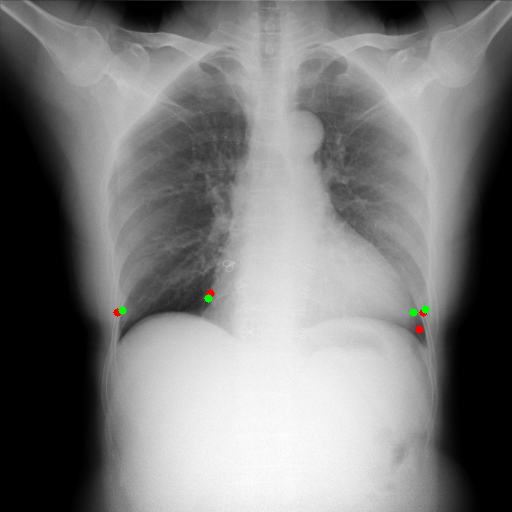

Supplement: Supplementary file 1 [file Presentation1.zip › Supplementary materials/s2/resUnet++/18.png]

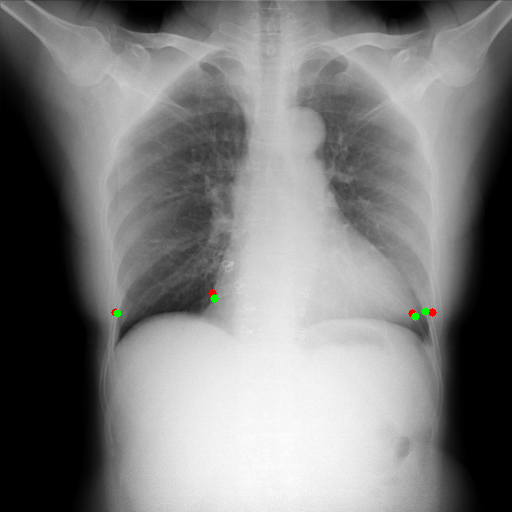

Supplement: Supplementary file 1 [file Presentation1.zip › Supplementary materials/s2/resUnet++/19.png]

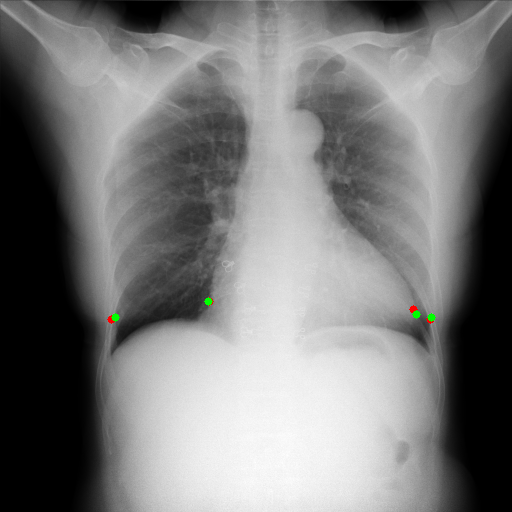

Supplement: Supplementary file 1 [file Presentation1.zip › Supplementary materials/s2/resUnet++/20.png]

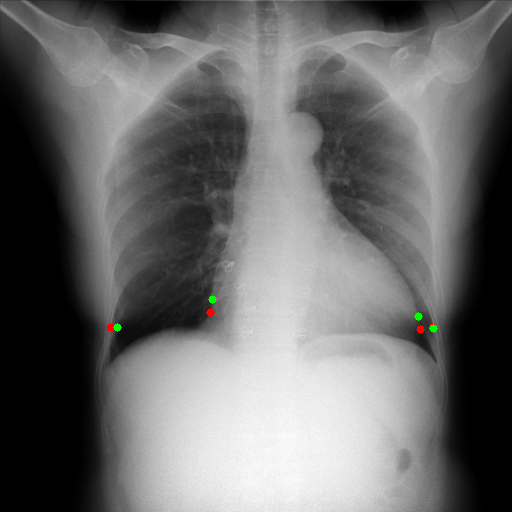

Supplement: Supplementary file 1 [file Presentation1.zip › Supplementary materials/s2/resUnet++/21.png]

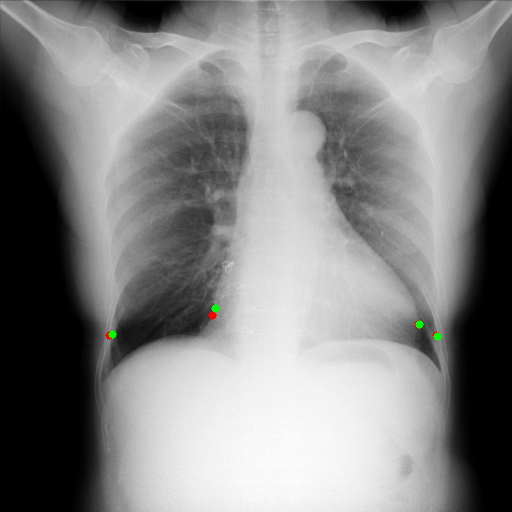

Supplement: Supplementary file 1 [file Presentation1.zip › Supplementary materials/s2/resUnet++/22.png]

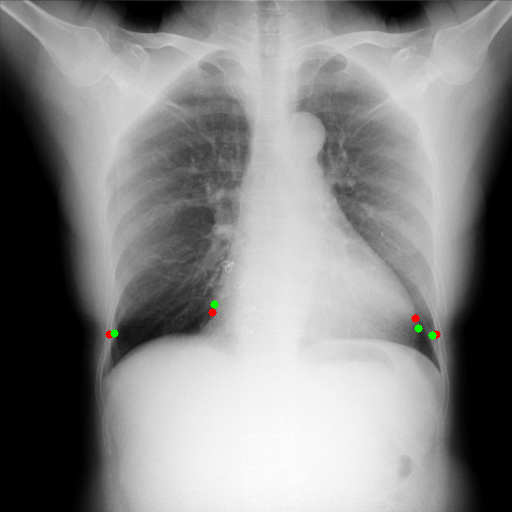

Supplement: Supplementary file 1 [file Presentation1.zip › Supplementary materials/s2/resUnet++/23.png]

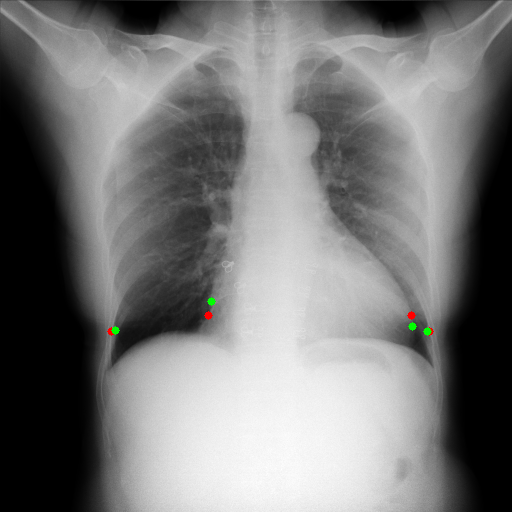

Supplement: Supplementary file 1 [file Presentation1.zip › Supplementary materials/s2/resUnet++/24.png]

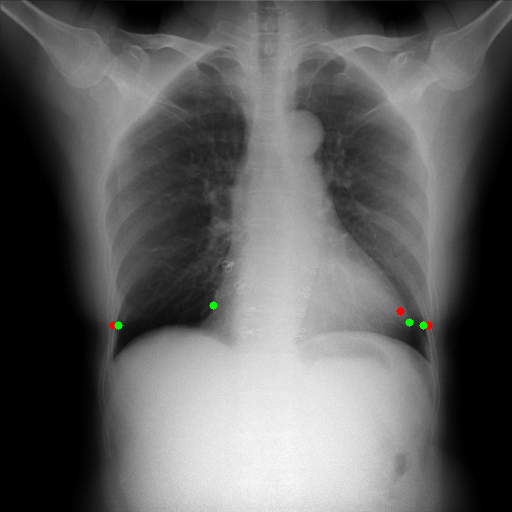

Supplement: Supplementary file 1 [file Presentation1.zip › Supplementary materials/s2/resUnet++/25.png]
