# Supplementary figures and images for: Automatic cardiothoracic ratio calculation based on lung fields abstracted from chest X-ray images without heart segmentation (part 1 of 2)
Source: Front Physiol. 2024 Aug 8;15:1416912. doi: 10.3389/fphys.2024.1416912 (PMC11338915; doi:10.3389/fphys.2024.1416912)

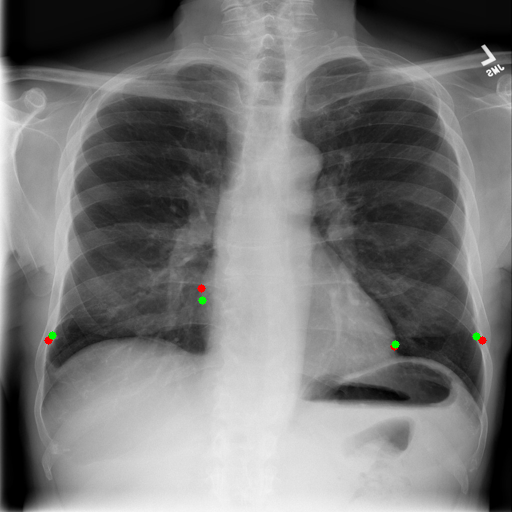

Supplement: Supplementary file 1 [file Presentation1.zip › Supplementary materials/s1/AttUnet/Normal-2217.png]

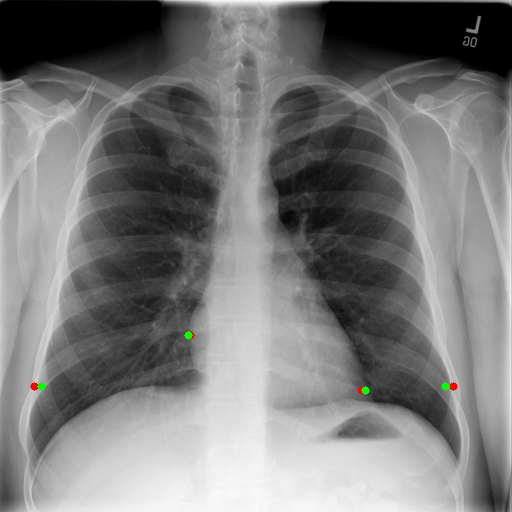

Supplement: Supplementary file 1 [file Presentation1.zip › Supplementary materials/s1/AttUnet/Normal-2226.png]

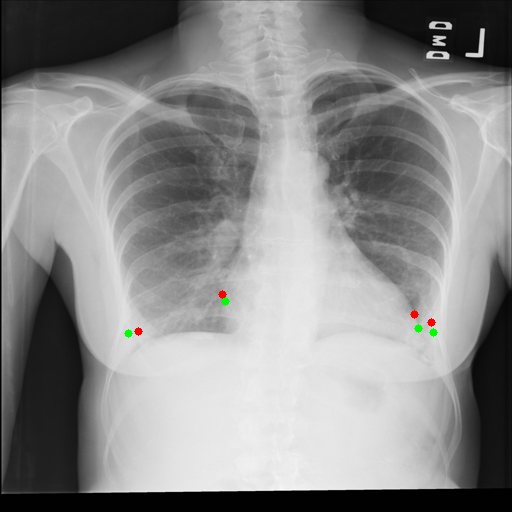

Supplement: Supplementary file 1 [file Presentation1.zip › Supplementary materials/s1/AttUnet/Normal-2231.png]

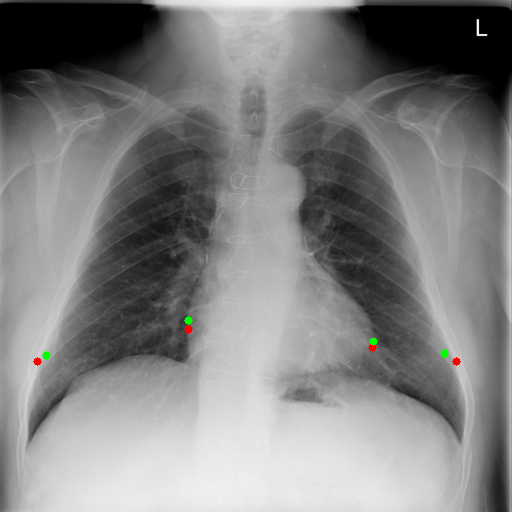

Supplement: Supplementary file 1 [file Presentation1.zip › Supplementary materials/s1/AttUnet/Normal-2236.png]

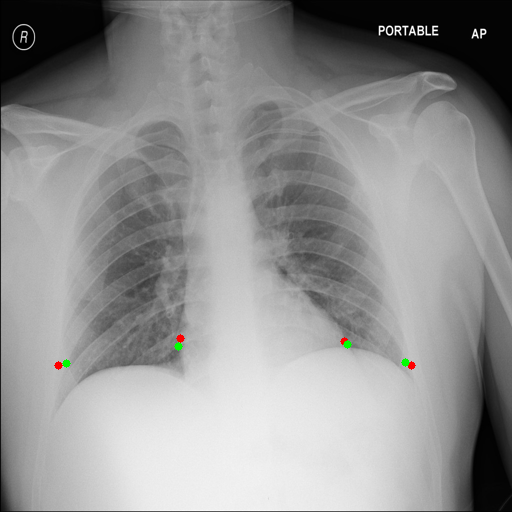

Supplement: Supplementary file 1 [file Presentation1.zip › Supplementary materials/s1/AttUnet/Normal-2245.png]

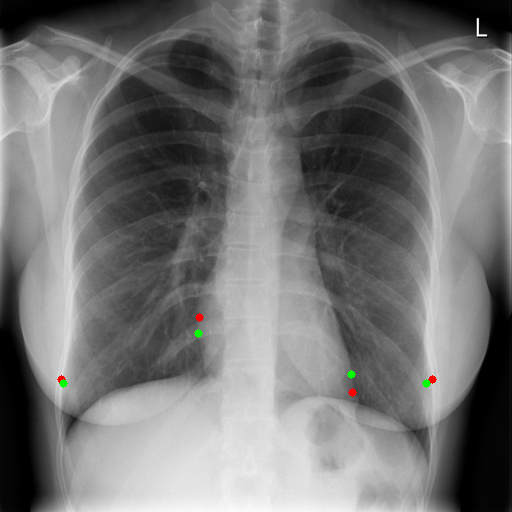

Supplement: Supplementary file 1 [file Presentation1.zip › Supplementary materials/s1/AttUnet/Normal-2247.png]

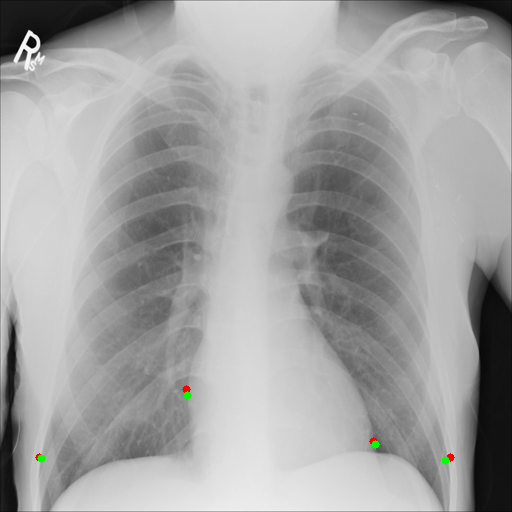

Supplement: Supplementary file 1 [file Presentation1.zip › Supplementary materials/s1/AttUnet/Normal-2252.png]

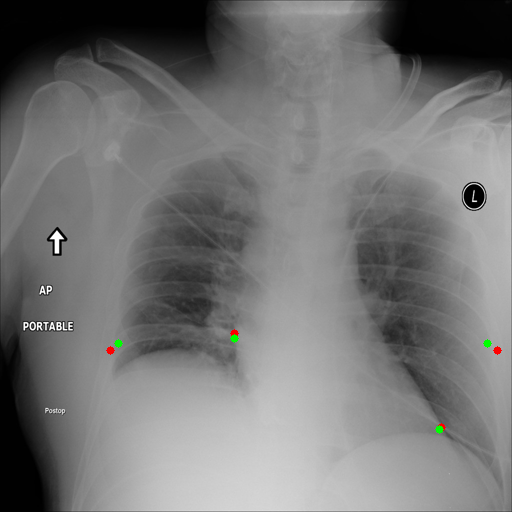

Supplement: Supplementary file 1 [file Presentation1.zip › Supplementary materials/s1/AttUnet/Normal-2275.png]

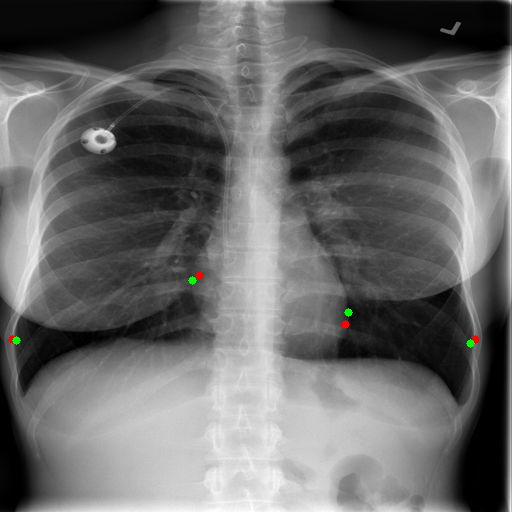

Supplement: Supplementary file 1 [file Presentation1.zip › Supplementary materials/s1/AttUnet/Normal-2287.png]

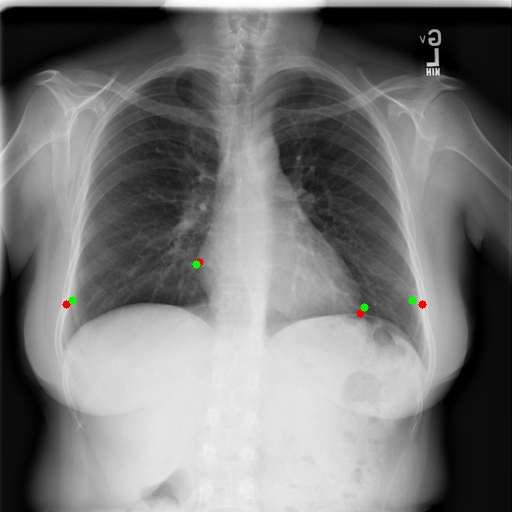

Supplement: Supplementary file 1 [file Presentation1.zip › Supplementary materials/s1/AttUnet/Normal-2311.png]

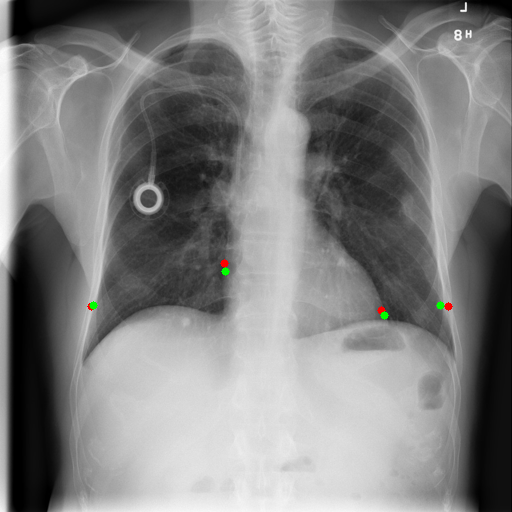

Supplement: Supplementary file 1 [file Presentation1.zip › Supplementary materials/s1/AttUnet/Normal-2320.png]

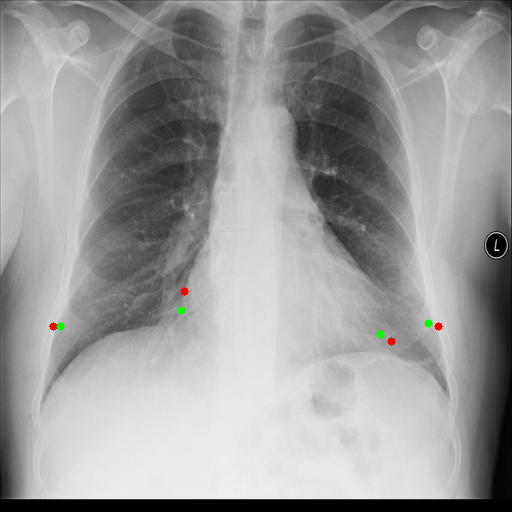

Supplement: Supplementary file 1 [file Presentation1.zip › Supplementary materials/s1/AttUnet/Normal-2327.png]

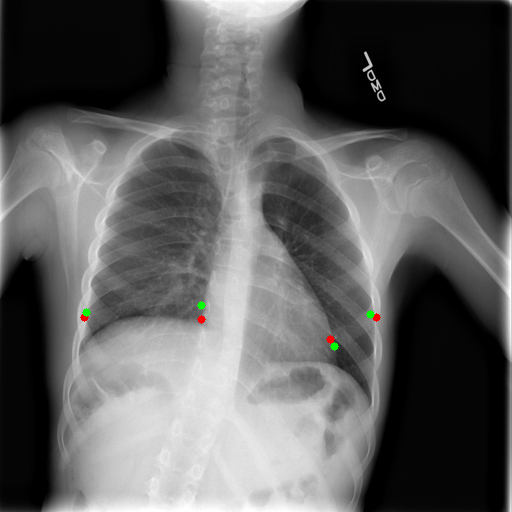

Supplement: Supplementary file 1 [file Presentation1.zip › Supplementary materials/s1/AttUnet/Normal-775.png]

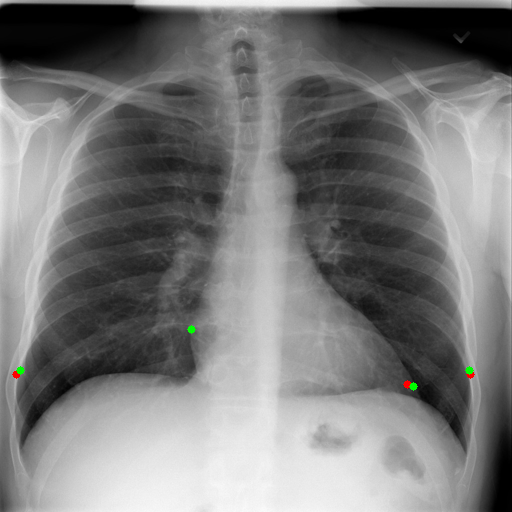

Supplement: Supplementary file 1 [file Presentation1.zip › Supplementary materials/s1/AttUnet/Normal-796.png]

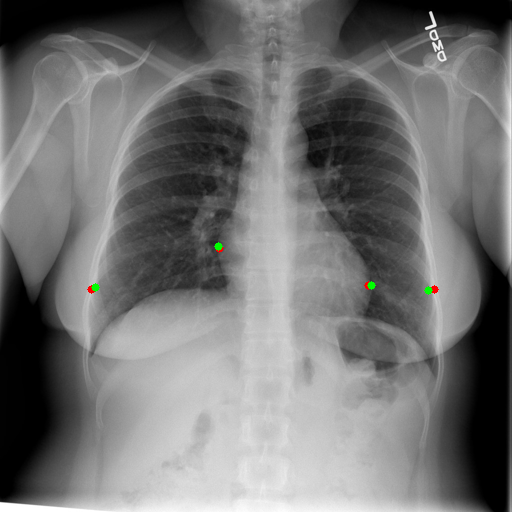

Supplement: Supplementary file 1 [file Presentation1.zip › Supplementary materials/s1/AttUnet/Normal-836.png]

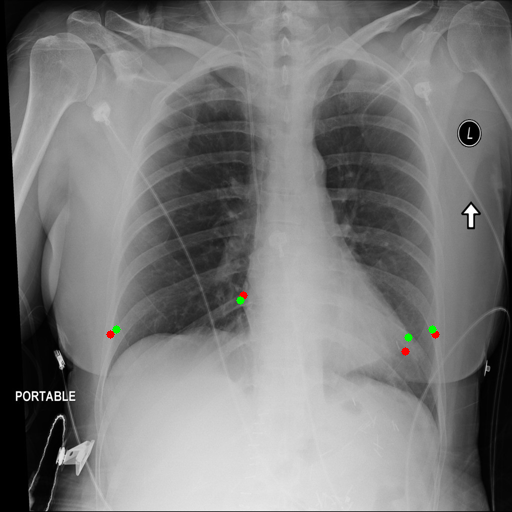

Supplement: Supplementary file 1 [file Presentation1.zip › Supplementary materials/s1/AttUnet/Normal-954.png]

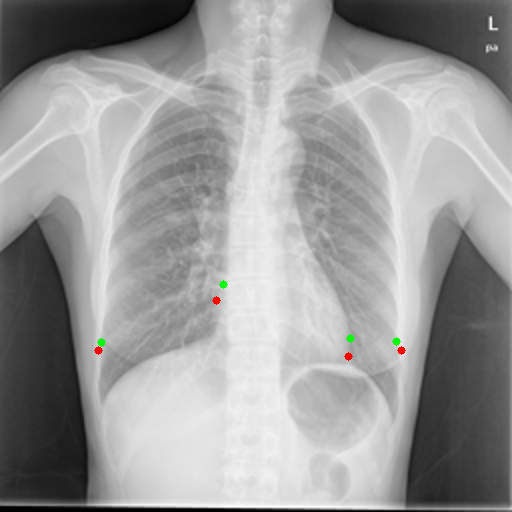

Supplement: Supplementary file 1 [file Presentation1.zip › Supplementary materials/s1/AttUnet/Tuberculosis-3370.png]

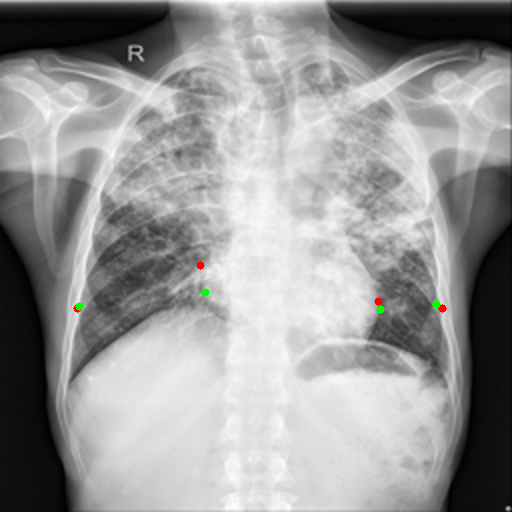

Supplement: Supplementary file 1 [file Presentation1.zip › Supplementary materials/s1/AttUnet/Tuberculosis-3373.png]

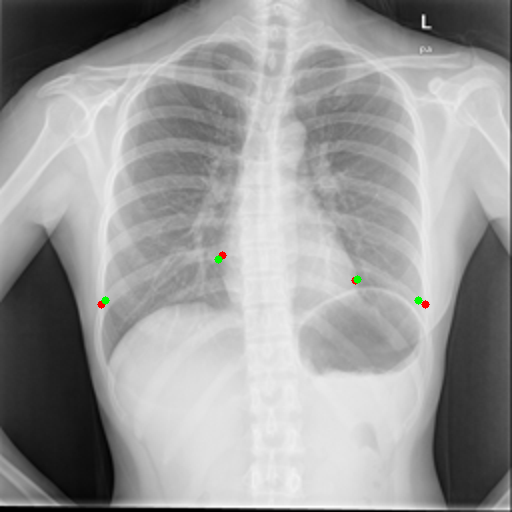

Supplement: Supplementary file 1 [file Presentation1.zip › Supplementary materials/s1/AttUnet/Tuberculosis-3403.png]

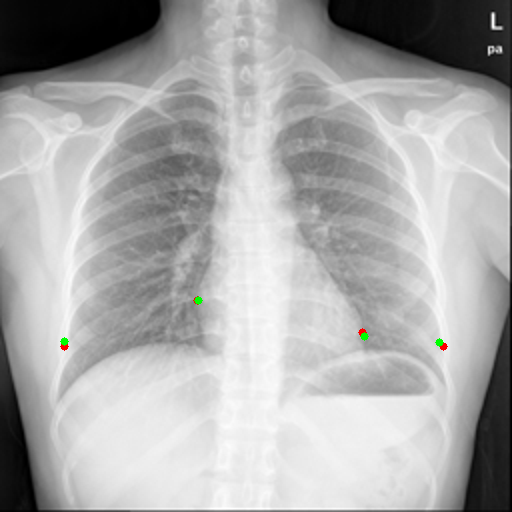

Supplement: Supplementary file 1 [file Presentation1.zip › Supplementary materials/s1/AttUnet/Tuberculosis-3413.png]

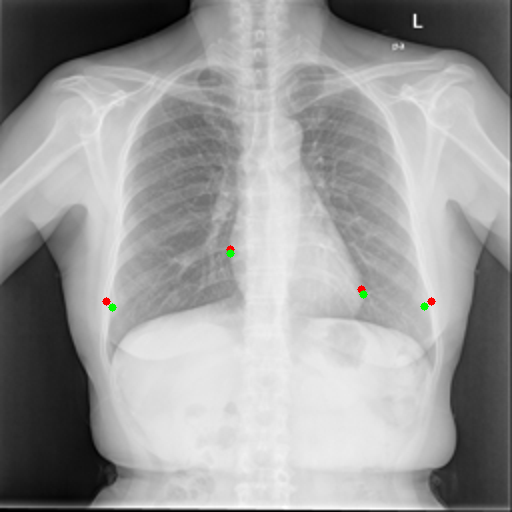

Supplement: Supplementary file 1 [file Presentation1.zip › Supplementary materials/s1/AttUnet/Tuberculosis-3418.png]

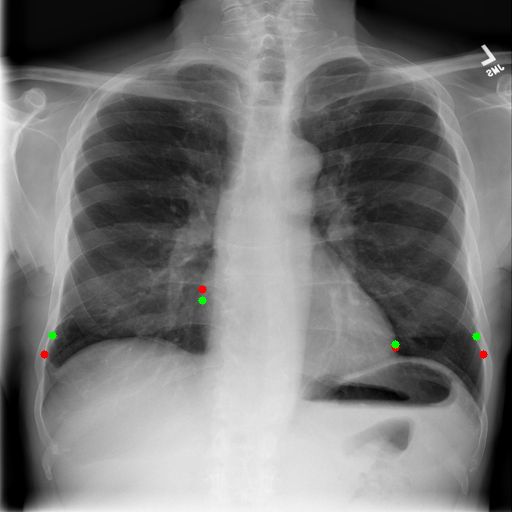

Supplement: Supplementary file 1 [file Presentation1.zip › Supplementary materials/s1/ResUnetPP++/Normal-2217.png]

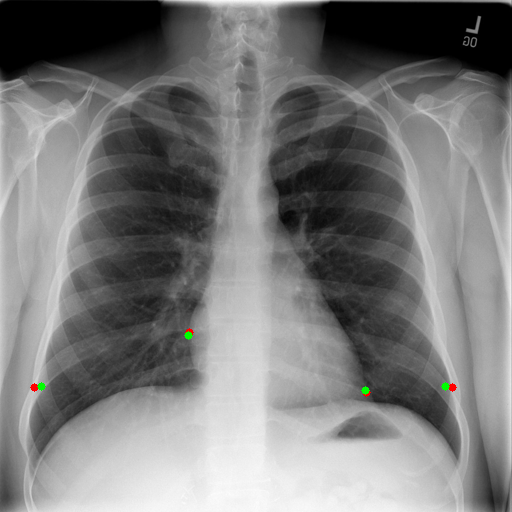

Supplement: Supplementary file 1 [file Presentation1.zip › Supplementary materials/s1/ResUnetPP++/Normal-2226.png]

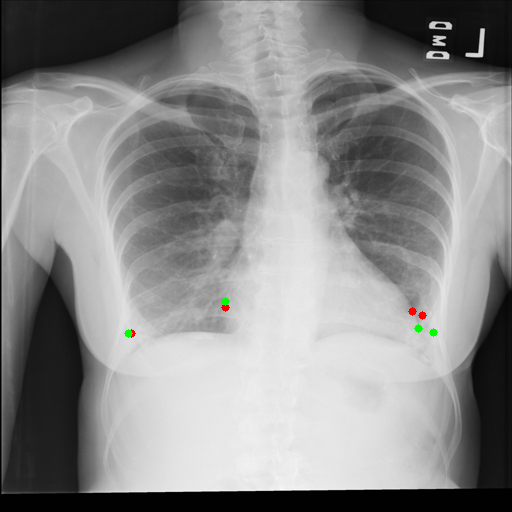

Supplement: Supplementary file 1 [file Presentation1.zip › Supplementary materials/s1/ResUnetPP++/Normal-2231.png]

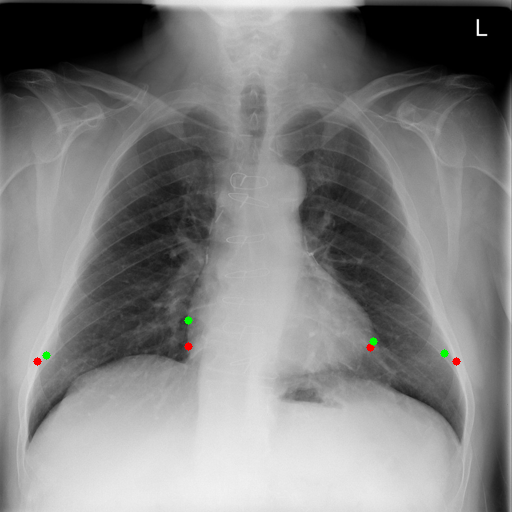

Supplement: Supplementary file 1 [file Presentation1.zip › Supplementary materials/s1/ResUnetPP++/Normal-2236.png]

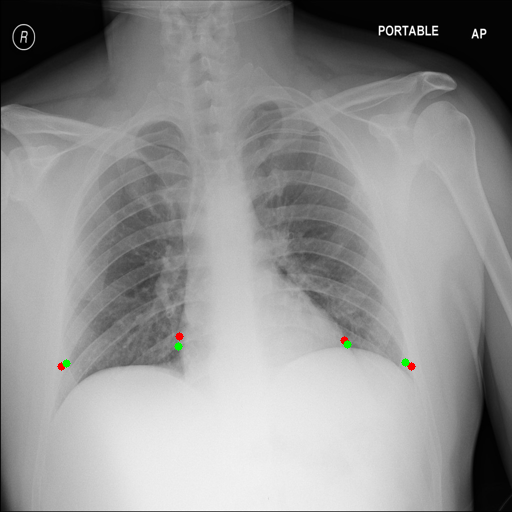

Supplement: Supplementary file 1 [file Presentation1.zip › Supplementary materials/s1/ResUnetPP++/Normal-2245.png]

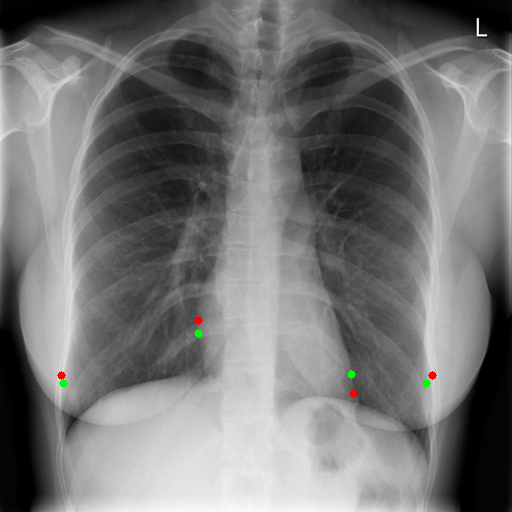

Supplement: Supplementary file 1 [file Presentation1.zip › Supplementary materials/s1/ResUnetPP++/Normal-2247.png]

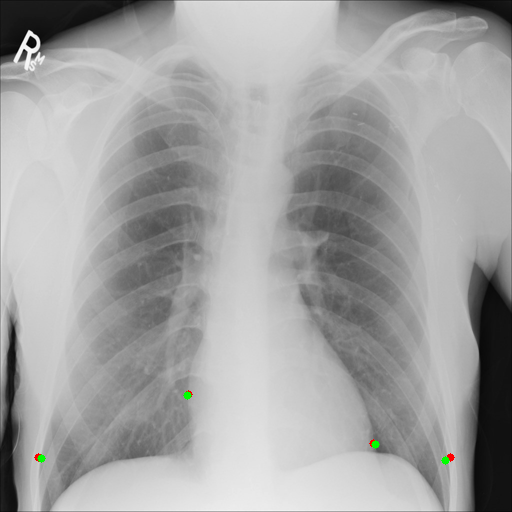

Supplement: Supplementary file 1 [file Presentation1.zip › Supplementary materials/s1/ResUnetPP++/Normal-2252.png]

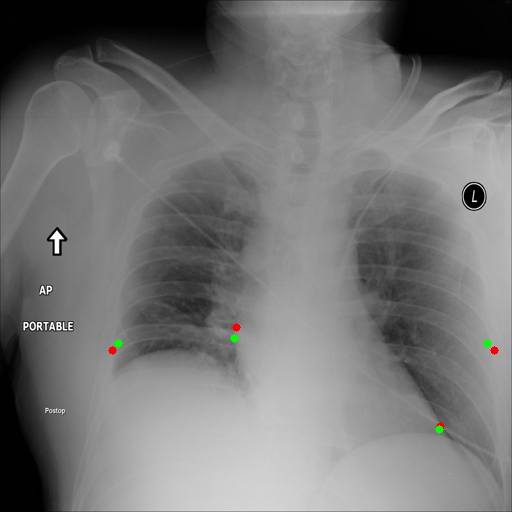

Supplement: Supplementary file 1 [file Presentation1.zip › Supplementary materials/s1/ResUnetPP++/Normal-2275.png]

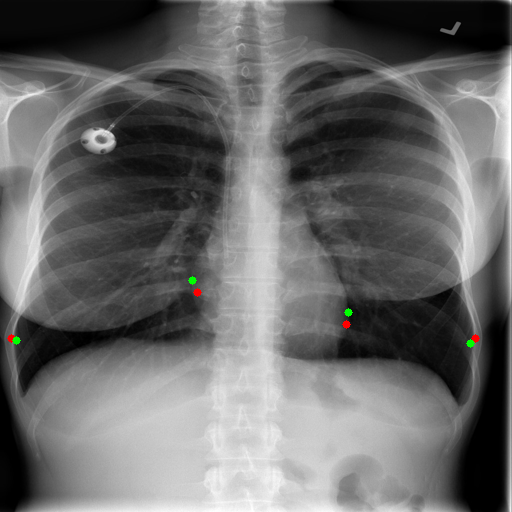

Supplement: Supplementary file 1 [file Presentation1.zip › Supplementary materials/s1/ResUnetPP++/Normal-2287.png]

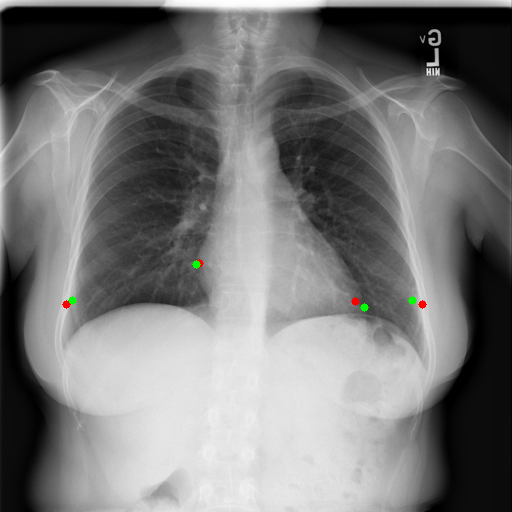

Supplement: Supplementary file 1 [file Presentation1.zip › Supplementary materials/s1/ResUnetPP++/Normal-2311.png]

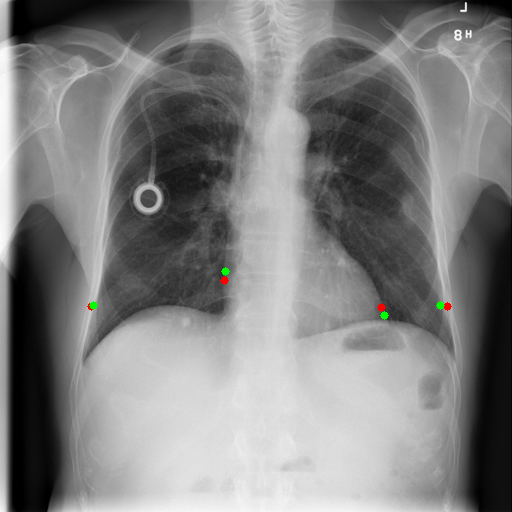

Supplement: Supplementary file 1 [file Presentation1.zip › Supplementary materials/s1/ResUnetPP++/Normal-2320.png]

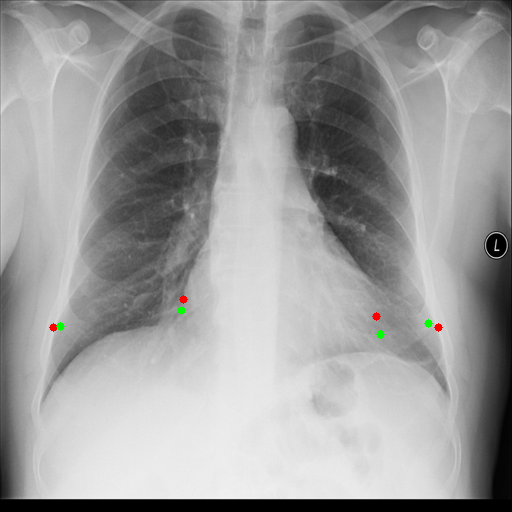

Supplement: Supplementary file 1 [file Presentation1.zip › Supplementary materials/s1/ResUnetPP++/Normal-2327.png]

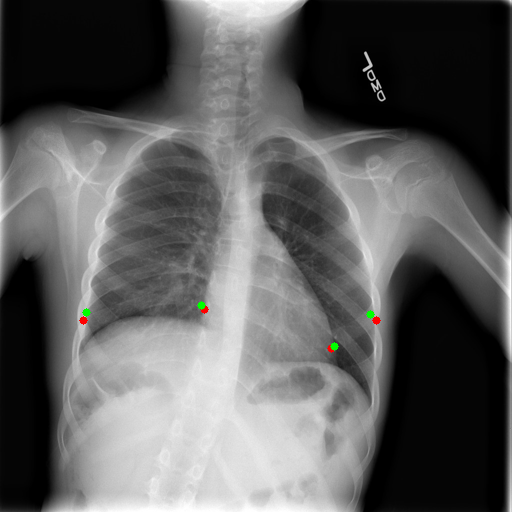

Supplement: Supplementary file 1 [file Presentation1.zip › Supplementary materials/s1/ResUnetPP++/Normal-775.png]

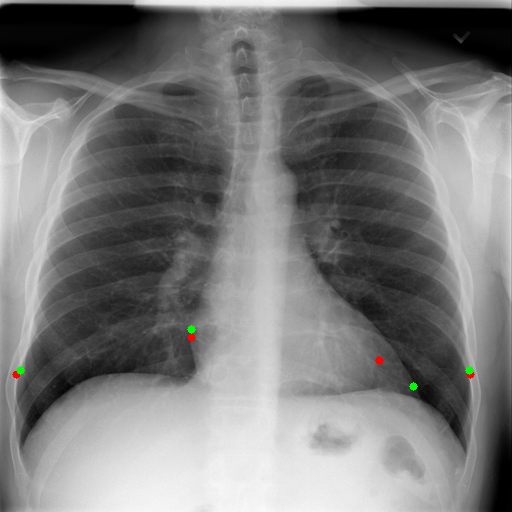

Supplement: Supplementary file 1 [file Presentation1.zip › Supplementary materials/s1/ResUnetPP++/Normal-796.png]

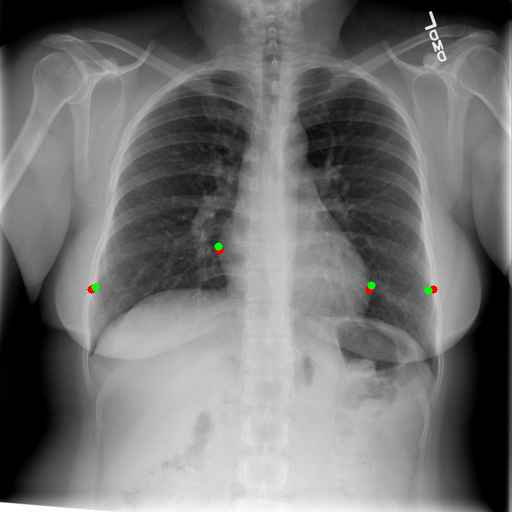

Supplement: Supplementary file 1 [file Presentation1.zip › Supplementary materials/s1/ResUnetPP++/Normal-836.png]

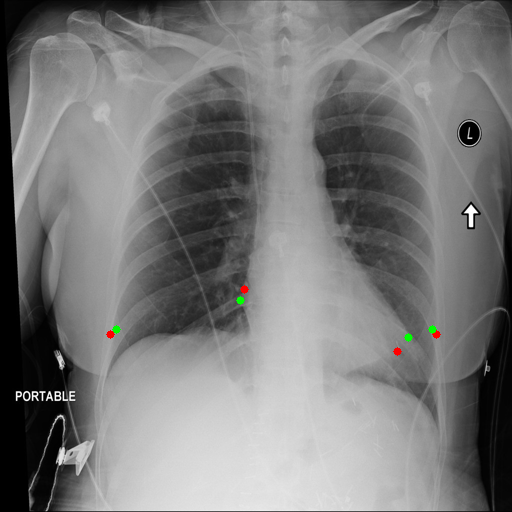

Supplement: Supplementary file 1 [file Presentation1.zip › Supplementary materials/s1/ResUnetPP++/Normal-954.png]

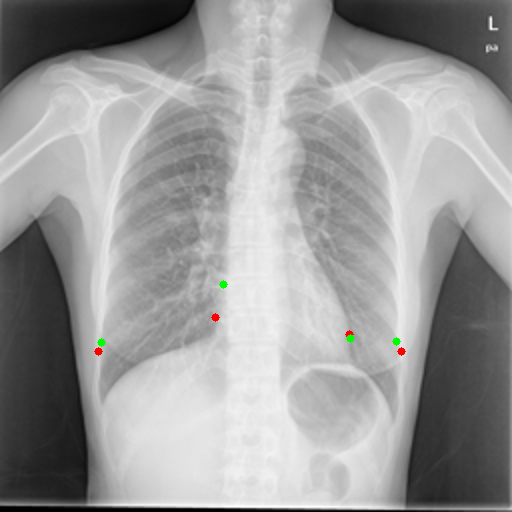

Supplement: Supplementary file 1 [file Presentation1.zip › Supplementary materials/s1/ResUnetPP++/Tuberculosis-3370.png]

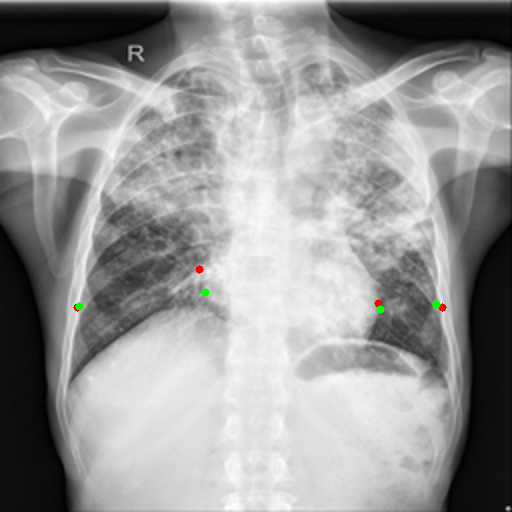

Supplement: Supplementary file 1 [file Presentation1.zip › Supplementary materials/s1/ResUnetPP++/Tuberculosis-3373.png]

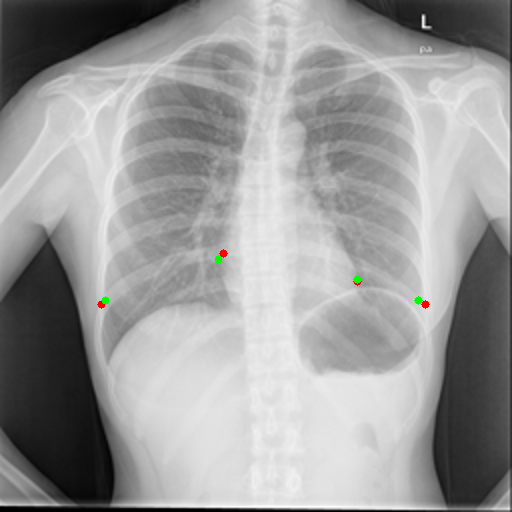

Supplement: Supplementary file 1 [file Presentation1.zip › Supplementary materials/s1/ResUnetPP++/Tuberculosis-3403.png]

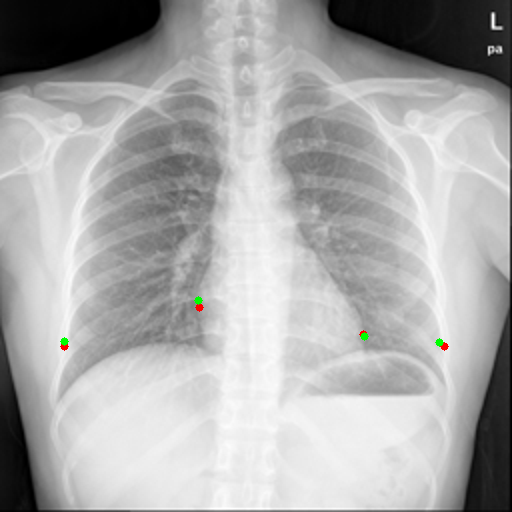

Supplement: Supplementary file 1 [file Presentation1.zip › Supplementary materials/s1/ResUnetPP++/Tuberculosis-3413.png]

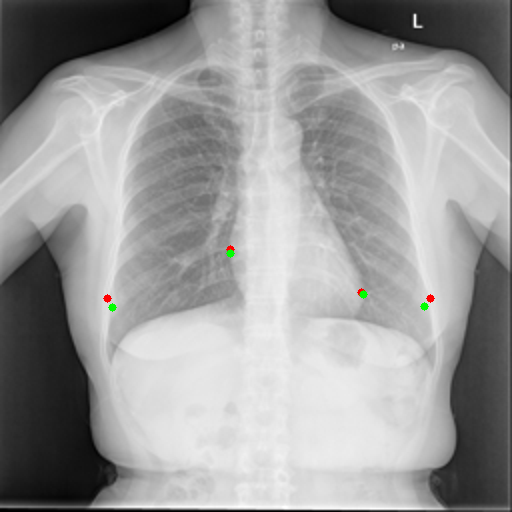

Supplement: Supplementary file 1 [file Presentation1.zip › Supplementary materials/s1/ResUnetPP++/Tuberculosis-3418.png]

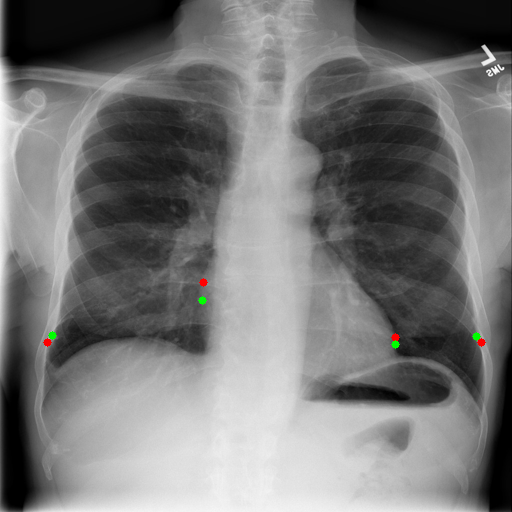

Supplement: Supplementary file 1 [file Presentation1.zip › Supplementary materials/s1/SegNet/Normal-2217.png]

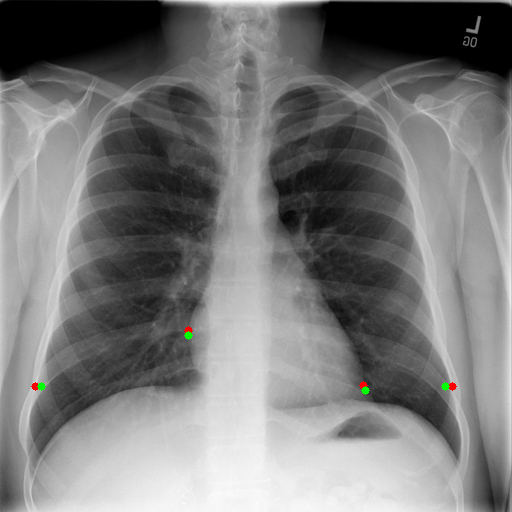

Supplement: Supplementary file 1 [file Presentation1.zip › Supplementary materials/s1/SegNet/Normal-2226.png]

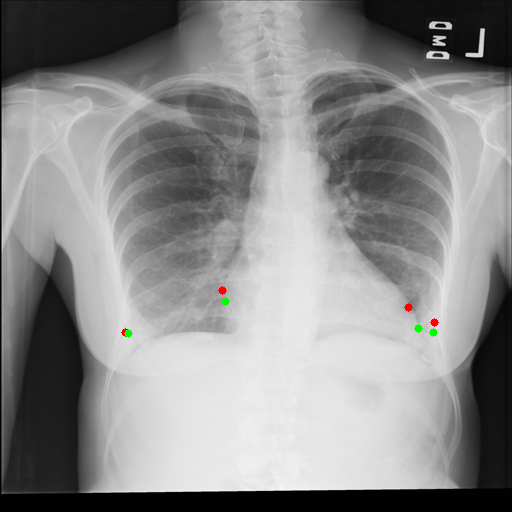

Supplement: Supplementary file 1 [file Presentation1.zip › Supplementary materials/s1/SegNet/Normal-2231.png]

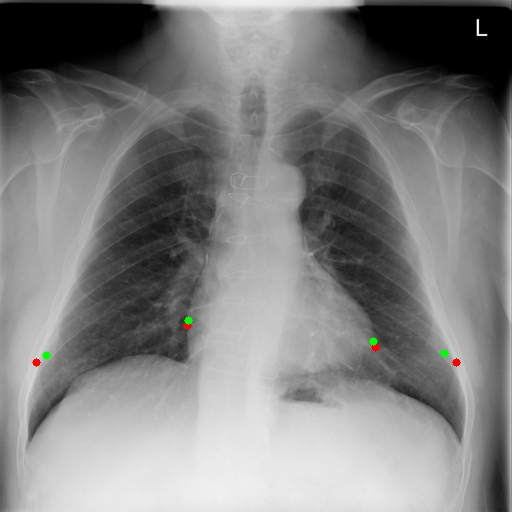

Supplement: Supplementary file 1 [file Presentation1.zip › Supplementary materials/s1/SegNet/Normal-2236.png]

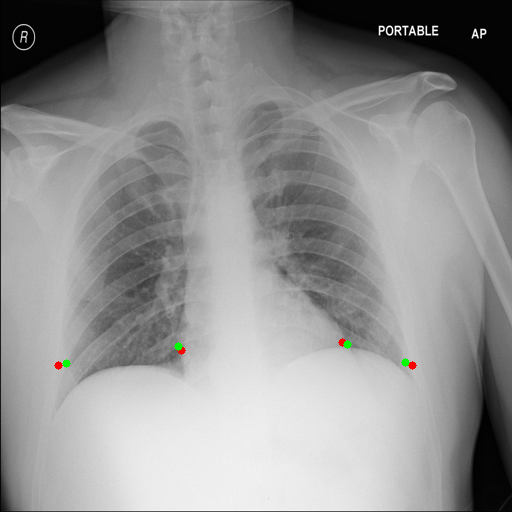

Supplement: Supplementary file 1 [file Presentation1.zip › Supplementary materials/s1/SegNet/Normal-2245.png]

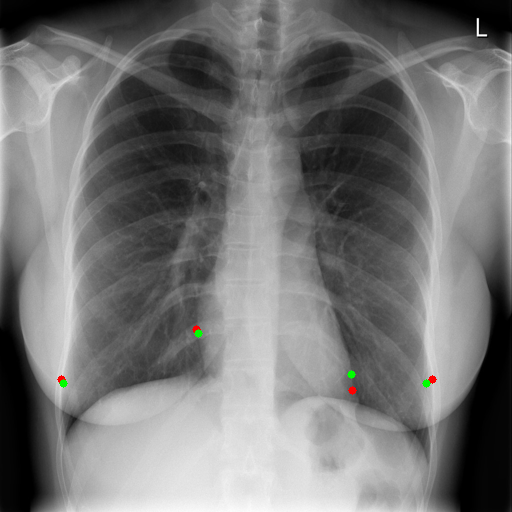

Supplement: Supplementary file 1 [file Presentation1.zip › Supplementary materials/s1/SegNet/Normal-2247.png]

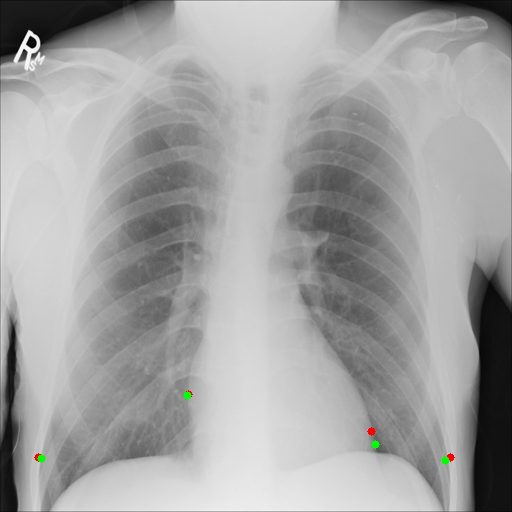

Supplement: Supplementary file 1 [file Presentation1.zip › Supplementary materials/s1/SegNet/Normal-2252.png]

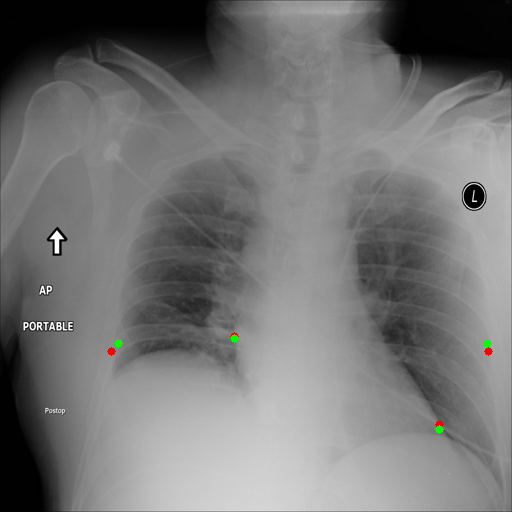

Supplement: Supplementary file 1 [file Presentation1.zip › Supplementary materials/s1/SegNet/Normal-2275.png]

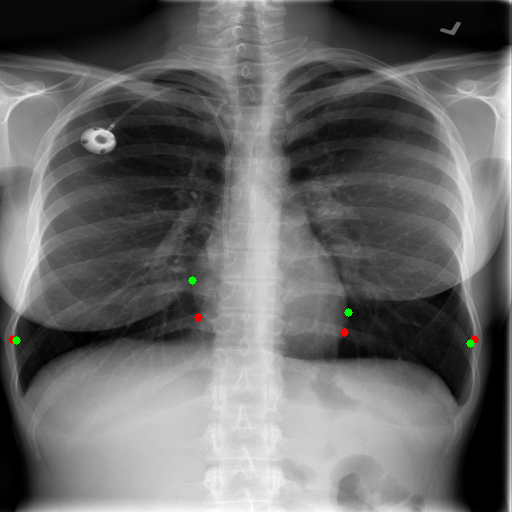

Supplement: Supplementary file 1 [file Presentation1.zip › Supplementary materials/s1/SegNet/Normal-2287.png]

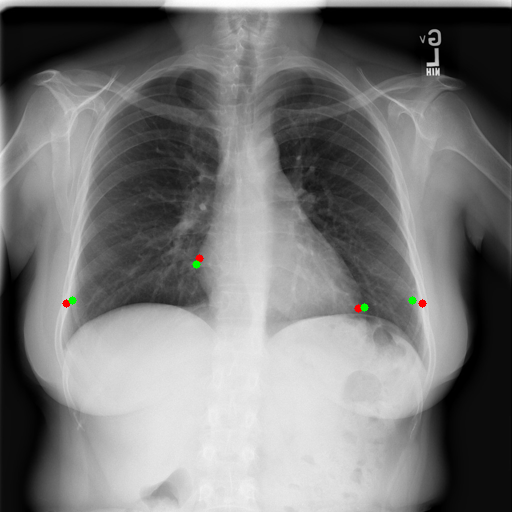

Supplement: Supplementary file 1 [file Presentation1.zip › Supplementary materials/s1/SegNet/Normal-2311.png]

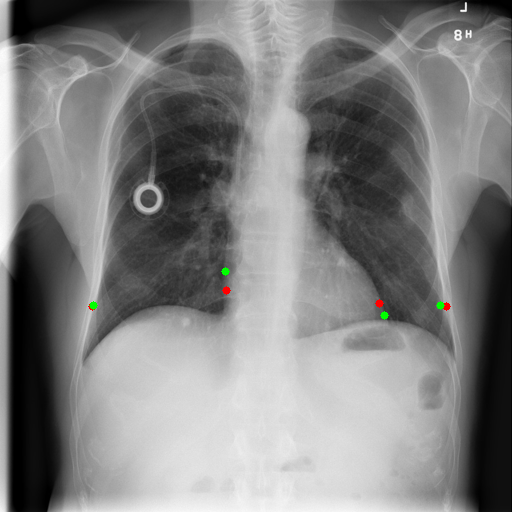

Supplement: Supplementary file 1 [file Presentation1.zip › Supplementary materials/s1/SegNet/Normal-2320.png]

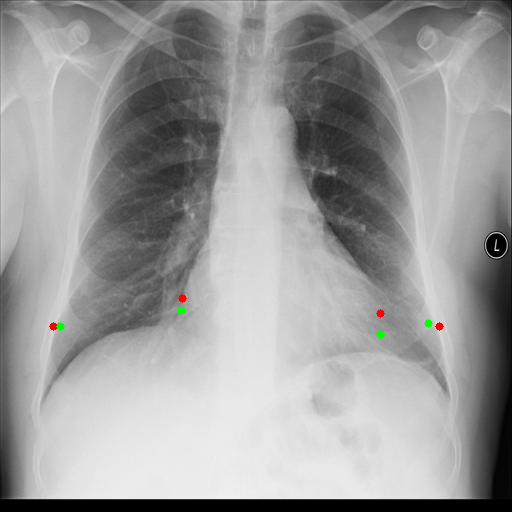

Supplement: Supplementary file 1 [file Presentation1.zip › Supplementary materials/s1/SegNet/Normal-2327.png]

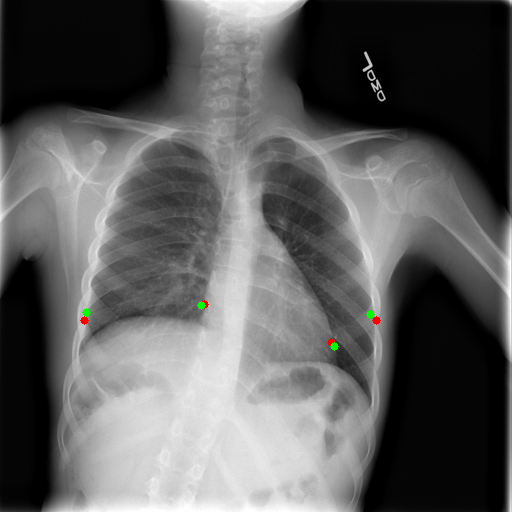

Supplement: Supplementary file 1 [file Presentation1.zip › Supplementary materials/s1/SegNet/Normal-775.png]

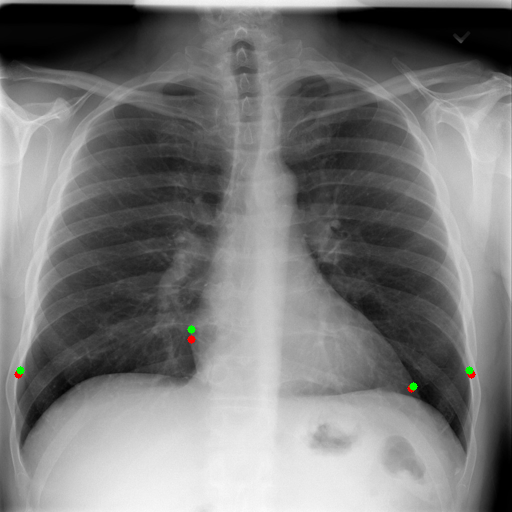

Supplement: Supplementary file 1 [file Presentation1.zip › Supplementary materials/s1/SegNet/Normal-796.png]

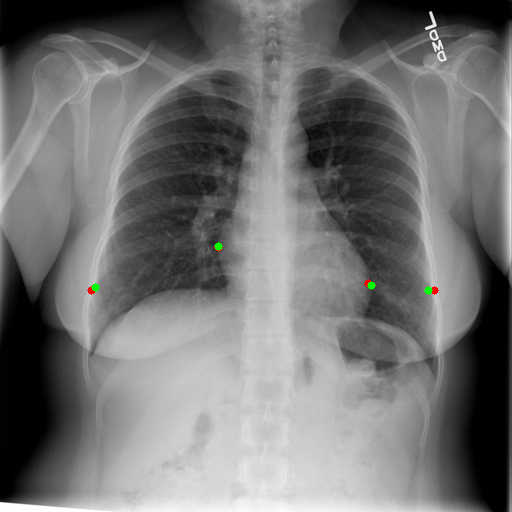

Supplement: Supplementary file 1 [file Presentation1.zip › Supplementary materials/s1/SegNet/Normal-836.png]

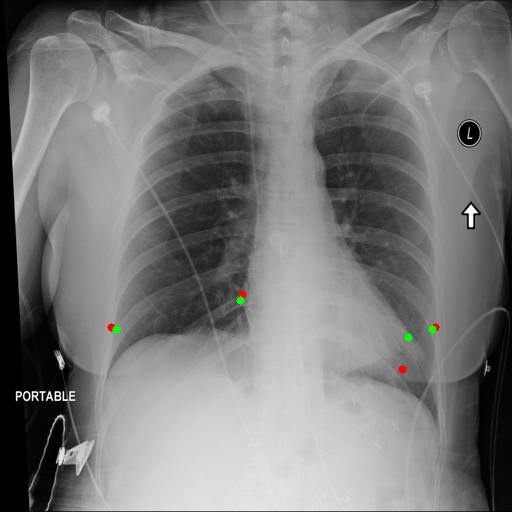

Supplement: Supplementary file 1 [file Presentation1.zip › Supplementary materials/s1/SegNet/Normal-954.png]

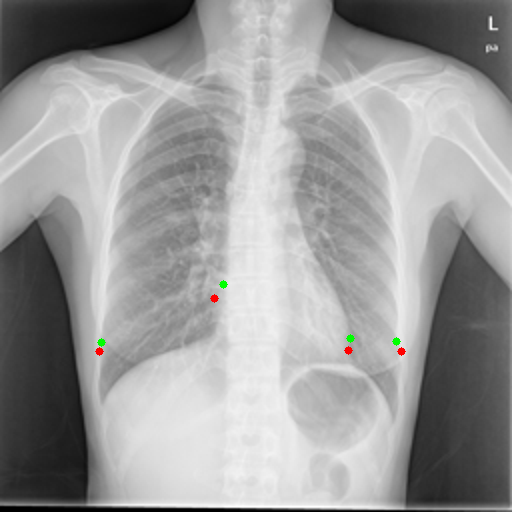

Supplement: Supplementary file 1 [file Presentation1.zip › Supplementary materials/s1/SegNet/Tuberculosis-3370.png]

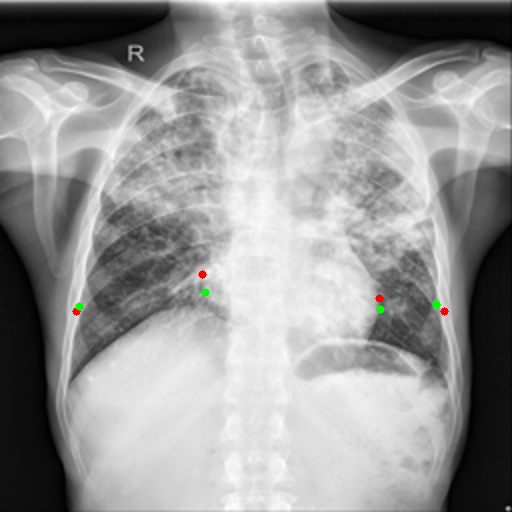

Supplement: Supplementary file 1 [file Presentation1.zip › Supplementary materials/s1/SegNet/Tuberculosis-3373.png]

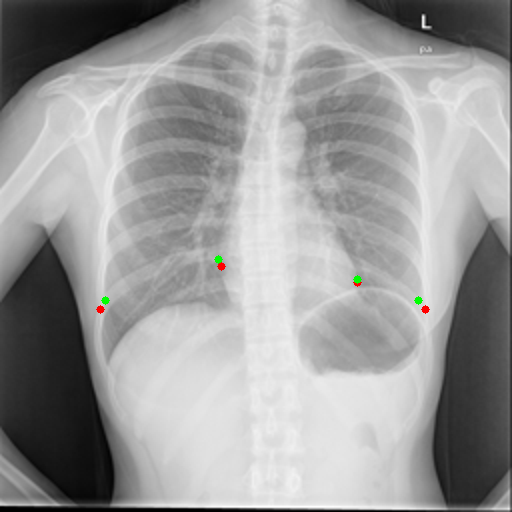

Supplement: Supplementary file 1 [file Presentation1.zip › Supplementary materials/s1/SegNet/Tuberculosis-3403.png]

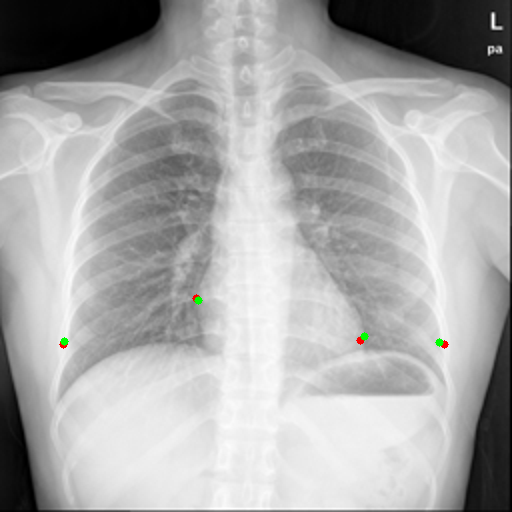

Supplement: Supplementary file 1 [file Presentation1.zip › Supplementary materials/s1/SegNet/Tuberculosis-3413.png]

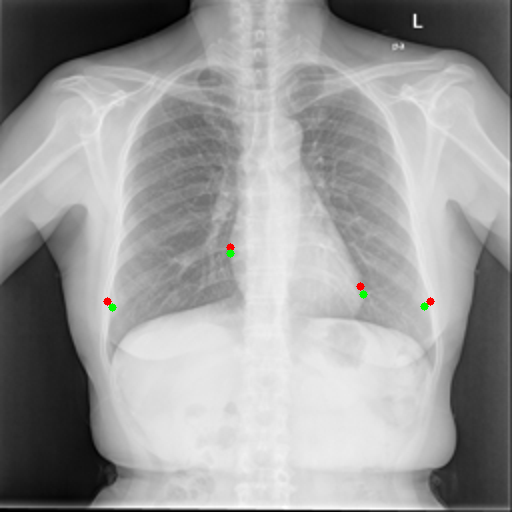

Supplement: Supplementary file 1 [file Presentation1.zip › Supplementary materials/s1/SegNet/Tuberculosis-3418.png]

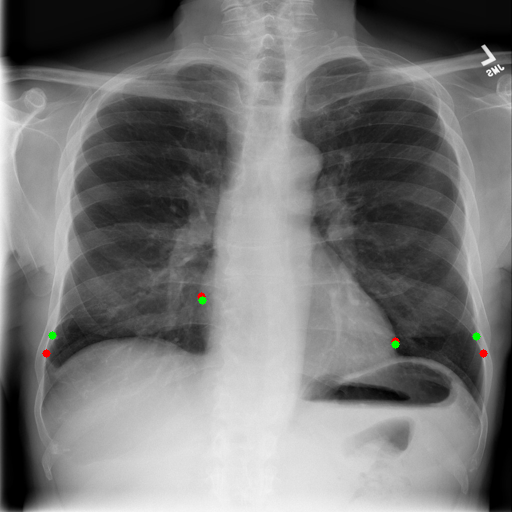

Supplement: Supplementary file 1 [file Presentation1.zip › Supplementary materials/s1/Unet/Normal-2217.png]

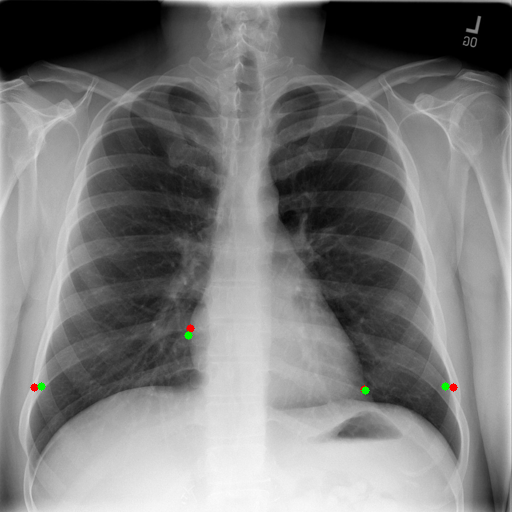

Supplement: Supplementary file 1 [file Presentation1.zip › Supplementary materials/s1/Unet/Normal-2226.png]

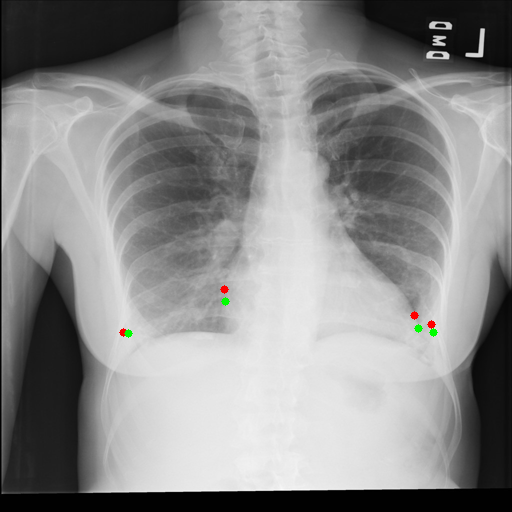

Supplement: Supplementary file 1 [file Presentation1.zip › Supplementary materials/s1/Unet/Normal-2231.png]

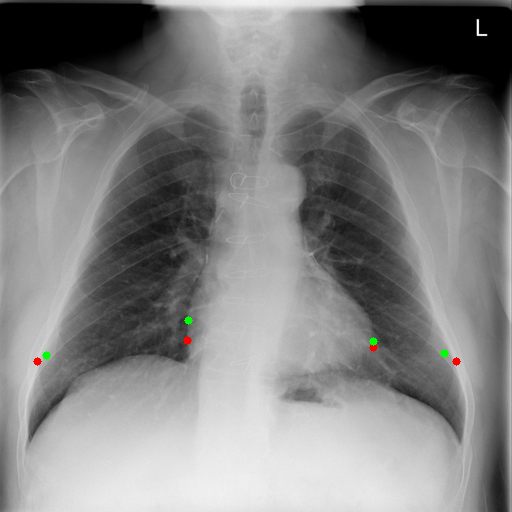

Supplement: Supplementary file 1 [file Presentation1.zip › Supplementary materials/s1/Unet/Normal-2236.png]

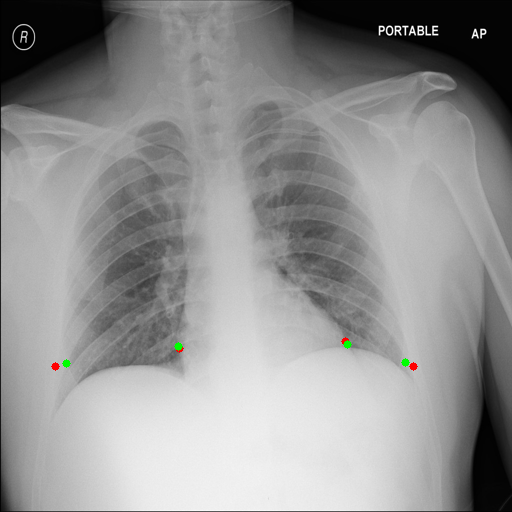

Supplement: Supplementary file 1 [file Presentation1.zip › Supplementary materials/s1/Unet/Normal-2245.png]

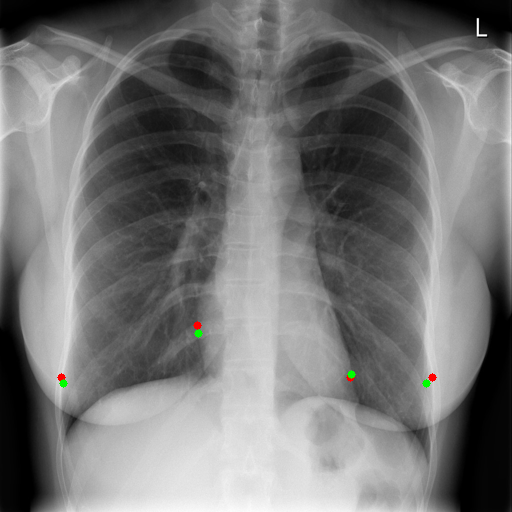

Supplement: Supplementary file 1 [file Presentation1.zip › Supplementary materials/s1/Unet/Normal-2247.png]

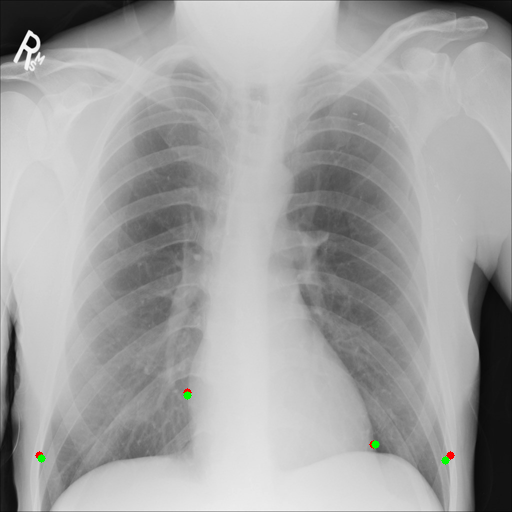

Supplement: Supplementary file 1 [file Presentation1.zip › Supplementary materials/s1/Unet/Normal-2252.png]

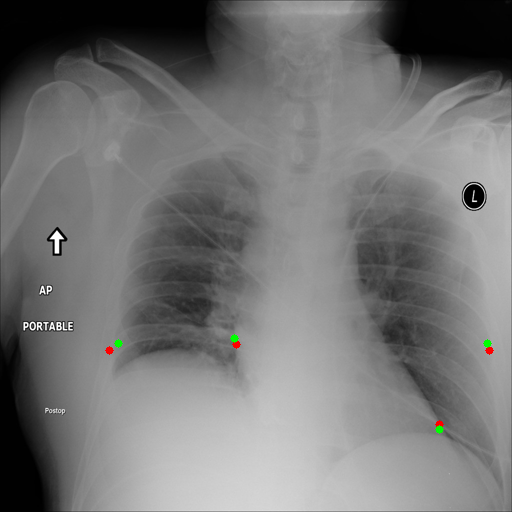

Supplement: Supplementary file 1 [file Presentation1.zip › Supplementary materials/s1/Unet/Normal-2275.png]

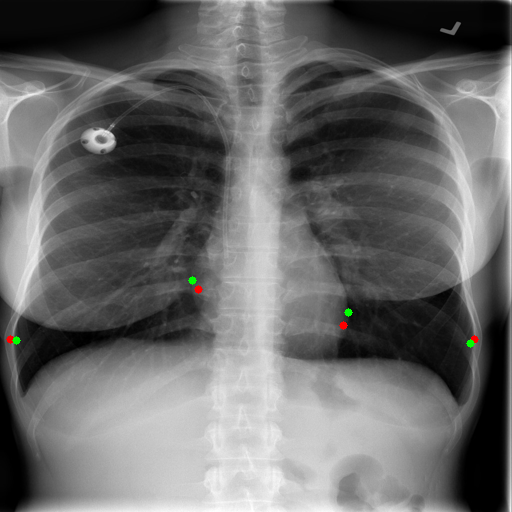

Supplement: Supplementary file 1 [file Presentation1.zip › Supplementary materials/s1/Unet/Normal-2287.png]

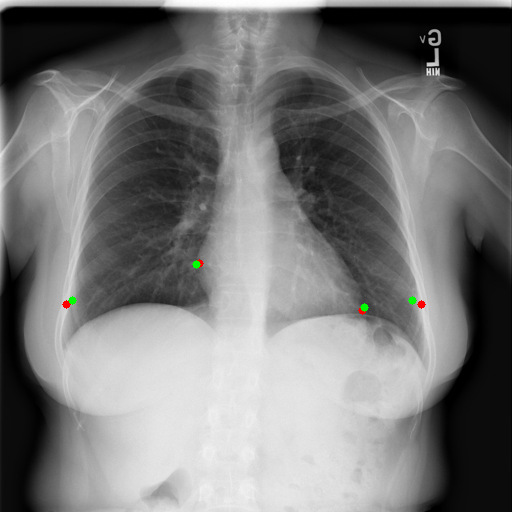

Supplement: Supplementary file 1 [file Presentation1.zip › Supplementary materials/s1/Unet/Normal-2311.png]

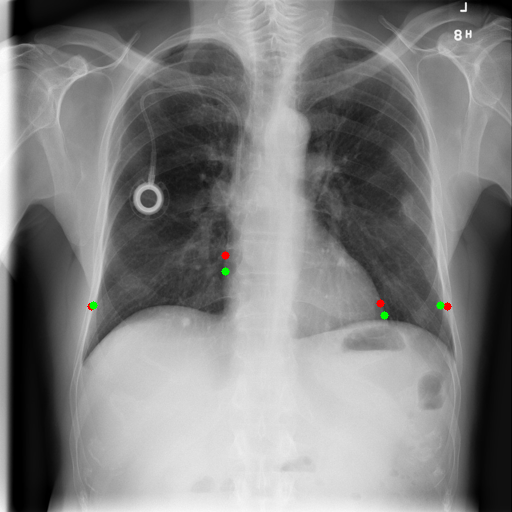

Supplement: Supplementary file 1 [file Presentation1.zip › Supplementary materials/s1/Unet/Normal-2320.png]

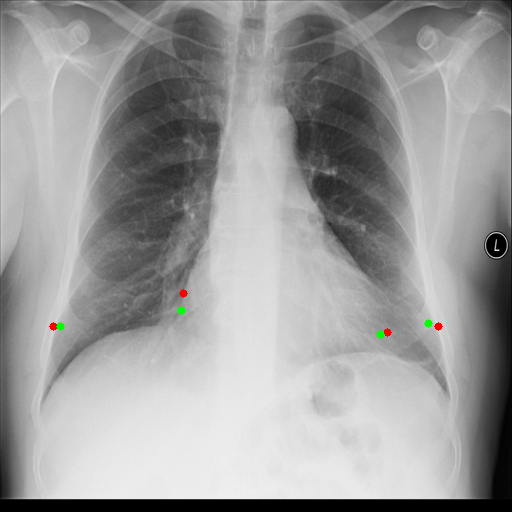

Supplement: Supplementary file 1 [file Presentation1.zip › Supplementary materials/s1/Unet/Normal-2327.png]

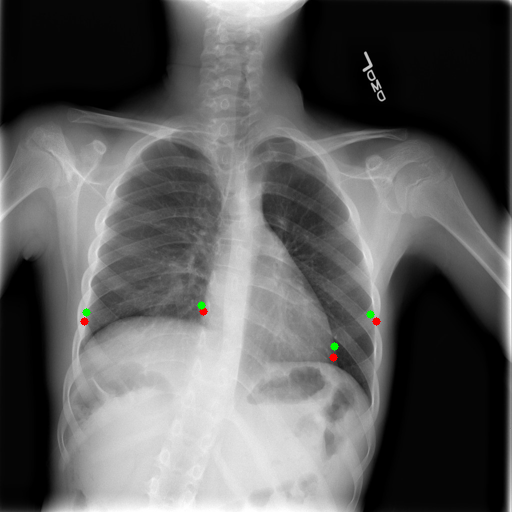

Supplement: Supplementary file 1 [file Presentation1.zip › Supplementary materials/s1/Unet/Normal-775.png]

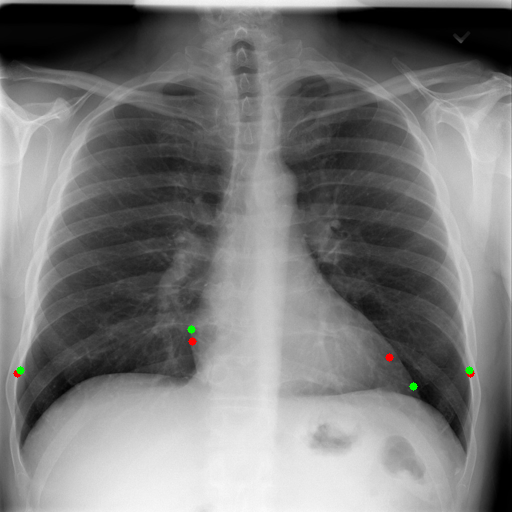

Supplement: Supplementary file 1 [file Presentation1.zip › Supplementary materials/s1/Unet/Normal-796.png]

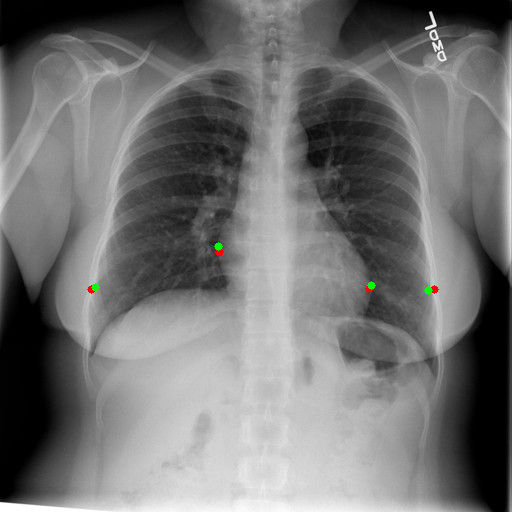

Supplement: Supplementary file 1 [file Presentation1.zip › Supplementary materials/s1/Unet/Normal-836.png]

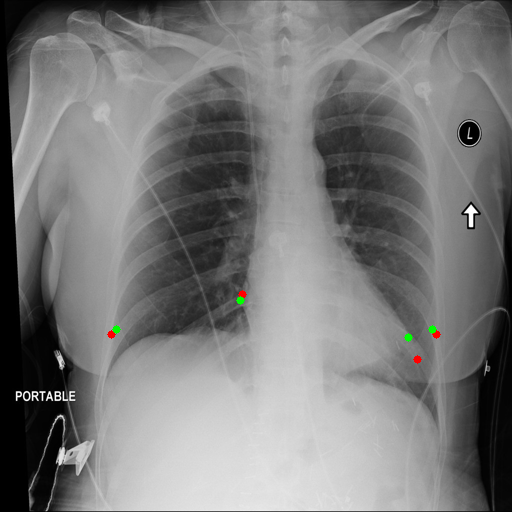

Supplement: Supplementary file 1 [file Presentation1.zip › Supplementary materials/s1/Unet/Normal-954.png]

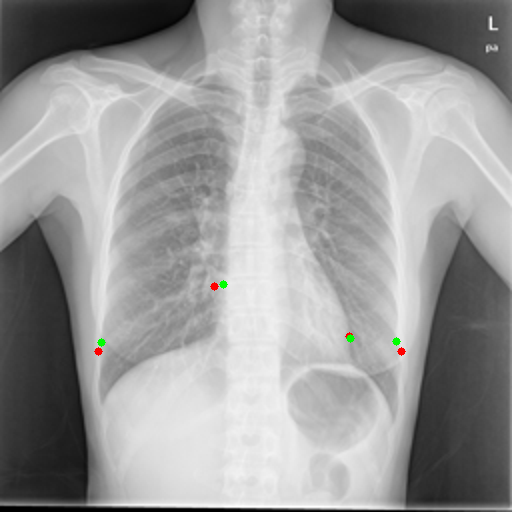

Supplement: Supplementary file 1 [file Presentation1.zip › Supplementary materials/s1/Unet/Tuberculosis-3370.png]

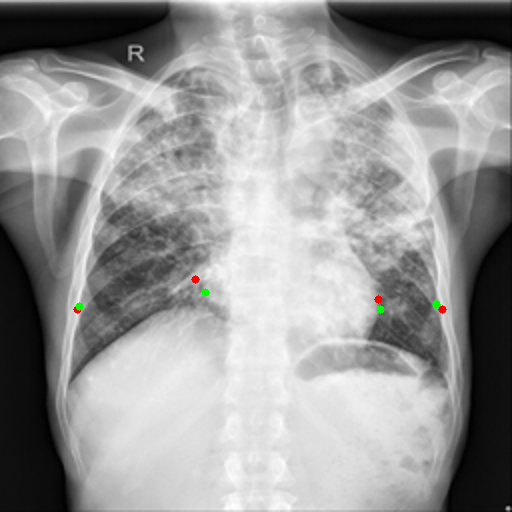

Supplement: Supplementary file 1 [file Presentation1.zip › Supplementary materials/s1/Unet/Tuberculosis-3373.png]

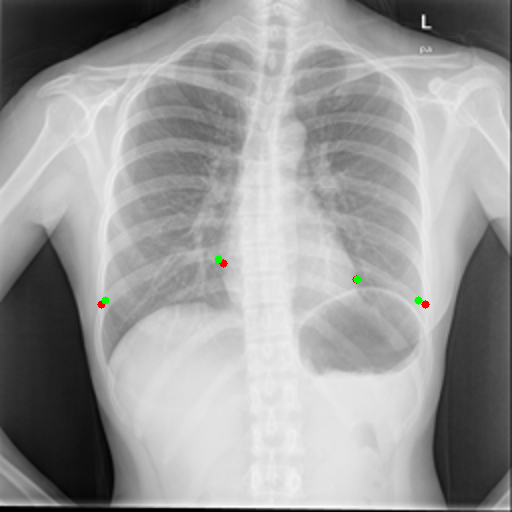

Supplement: Supplementary file 1 [file Presentation1.zip › Supplementary materials/s1/Unet/Tuberculosis-3403.png]

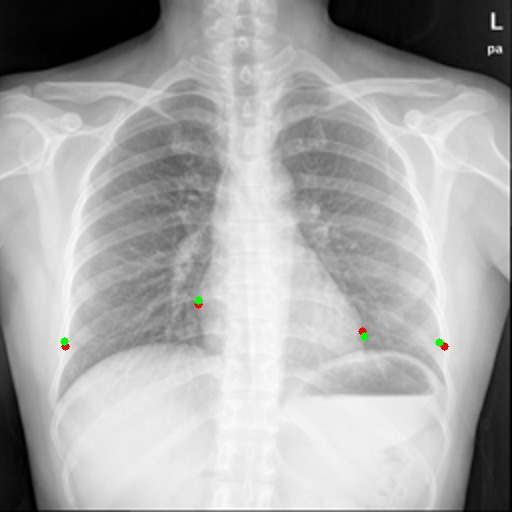

Supplement: Supplementary file 1 [file Presentation1.zip › Supplementary materials/s1/Unet/Tuberculosis-3413.png]

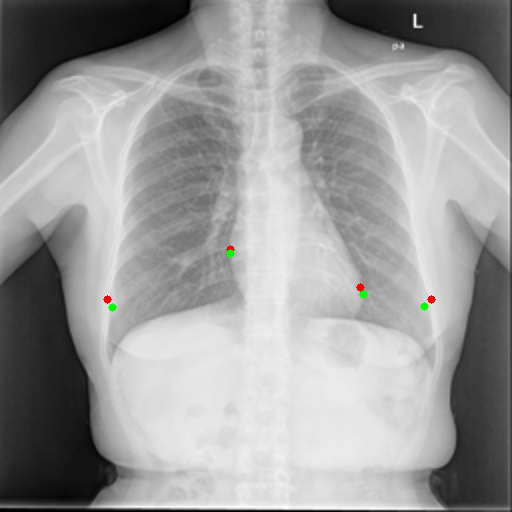

Supplement: Supplementary file 1 [file Presentation1.zip › Supplementary materials/s1/Unet/Tuberculosis-3418.png]

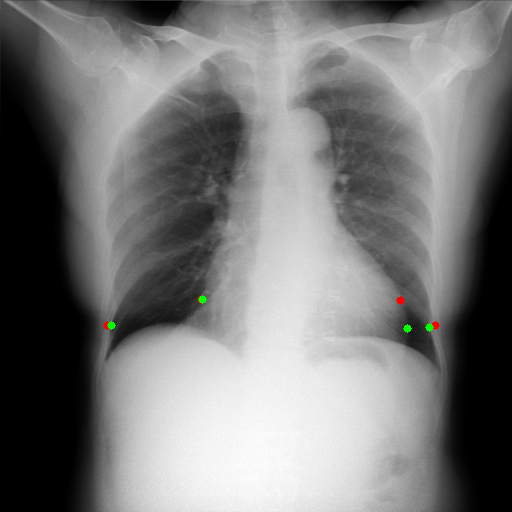

Supplement: Supplementary file 1 [file Presentation1.zip › Supplementary materials/s2/AttUnet/13.png]

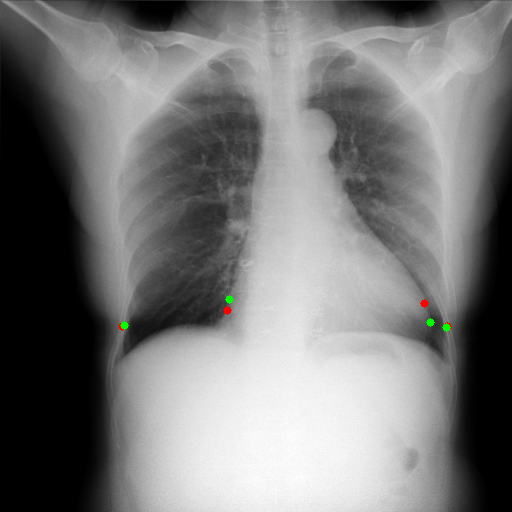

Supplement: Supplementary file 1 [file Presentation1.zip › Supplementary materials/s2/AttUnet/14.png]

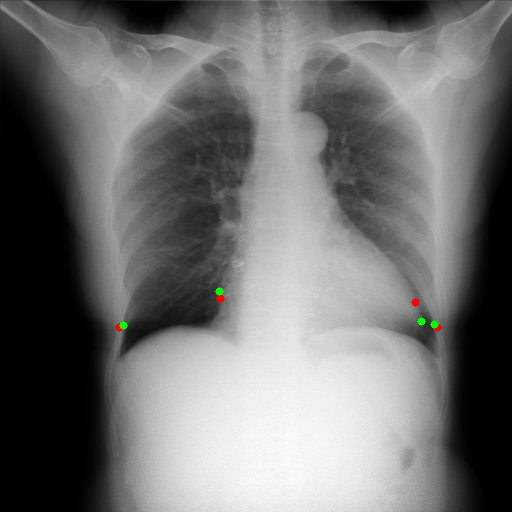

Supplement: Supplementary file 1 [file Presentation1.zip › Supplementary materials/s2/AttUnet/15.png]

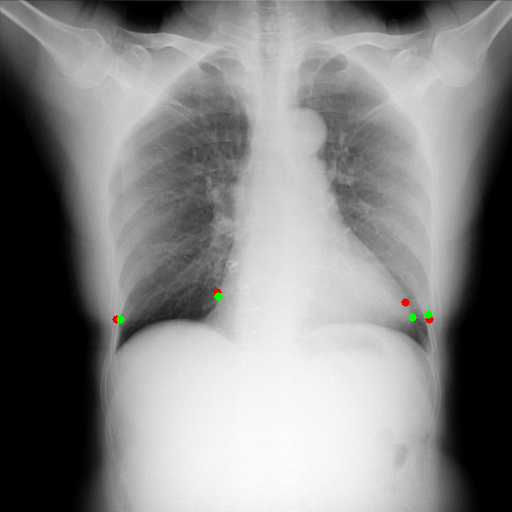

Supplement: Supplementary file 1 [file Presentation1.zip › Supplementary materials/s2/AttUnet/16.png]

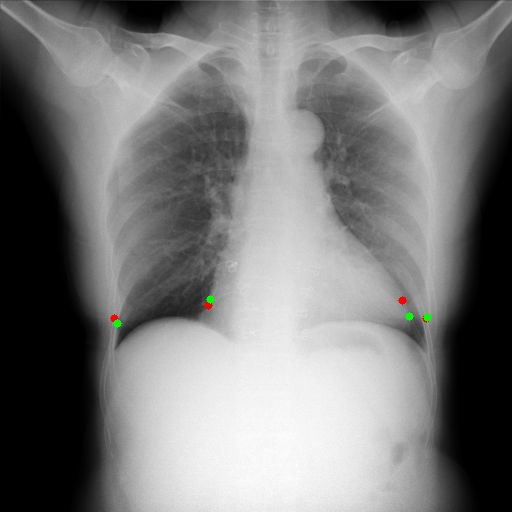

Supplement: Supplementary file 1 [file Presentation1.zip › Supplementary materials/s2/AttUnet/17.png]

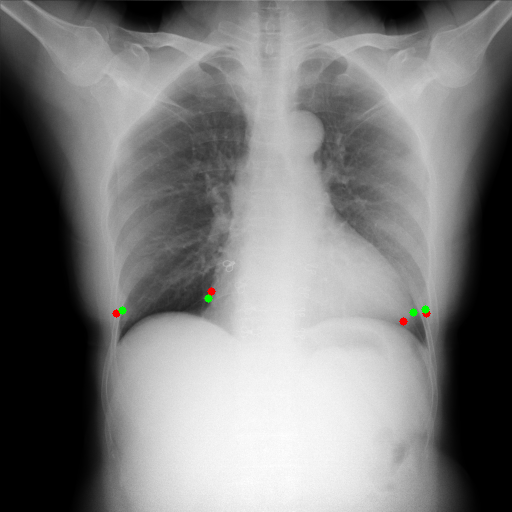

Supplement: Supplementary file 1 [file Presentation1.zip › Supplementary materials/s2/AttUnet/18.png]

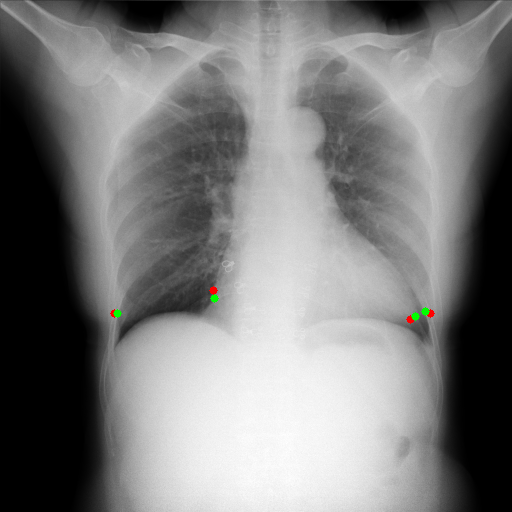

Supplement: Supplementary file 1 [file Presentation1.zip › Supplementary materials/s2/AttUnet/19.png]

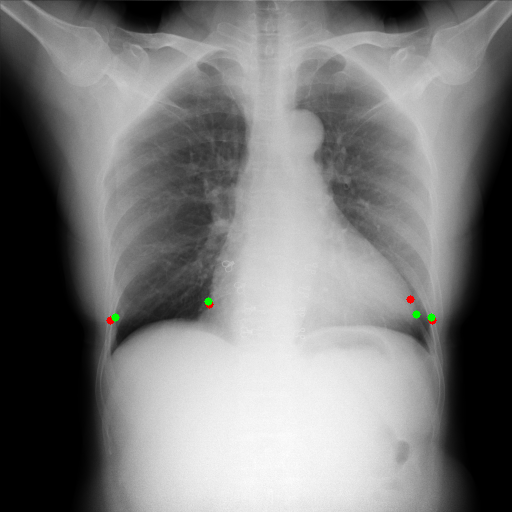

Supplement: Supplementary file 1 [file Presentation1.zip › Supplementary materials/s2/AttUnet/20.png]

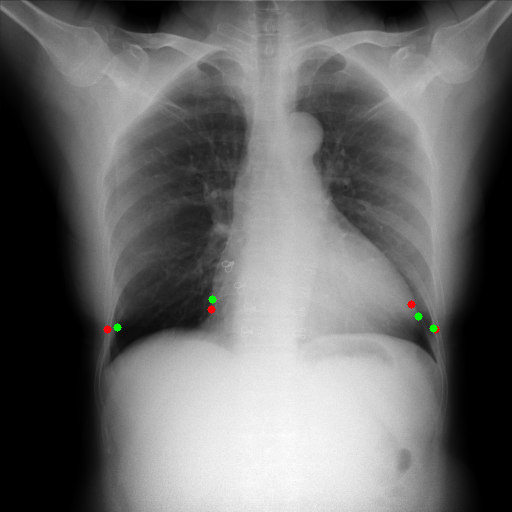

Supplement: Supplementary file 1 [file Presentation1.zip › Supplementary materials/s2/AttUnet/21.png]

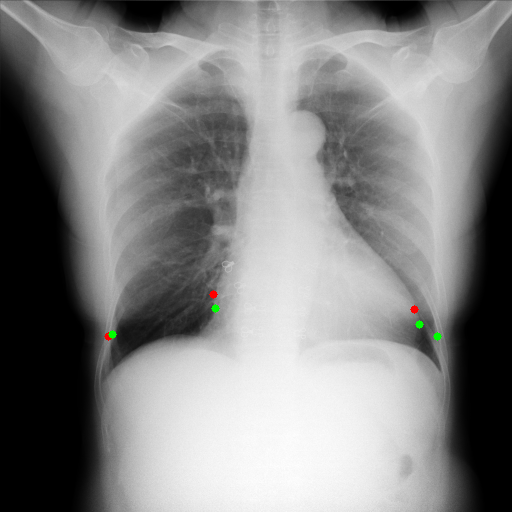

Supplement: Supplementary file 1 [file Presentation1.zip › Supplementary materials/s2/AttUnet/22.png]

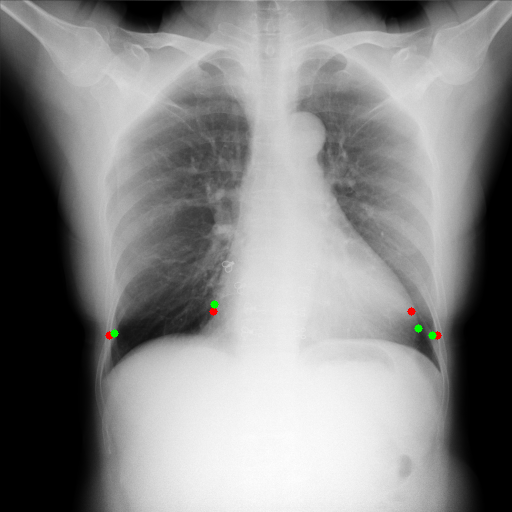

Supplement: Supplementary file 1 [file Presentation1.zip › Supplementary materials/s2/AttUnet/23.png]

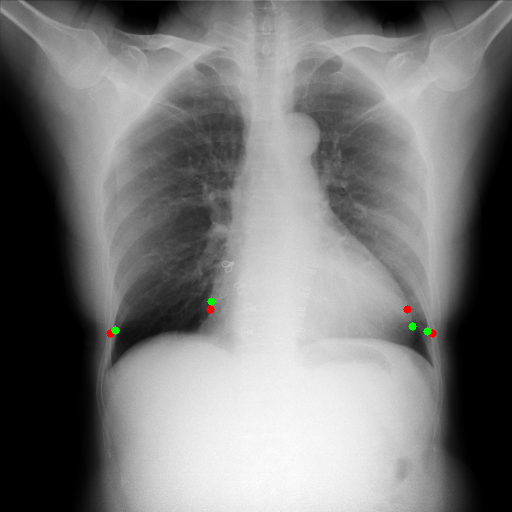

Supplement: Supplementary file 1 [file Presentation1.zip › Supplementary materials/s2/AttUnet/24.png]

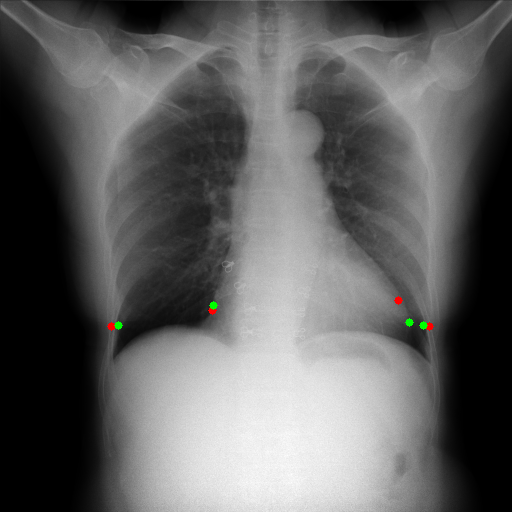

Supplement: Supplementary file 1 [file Presentation1.zip › Supplementary materials/s2/AttUnet/25.png]

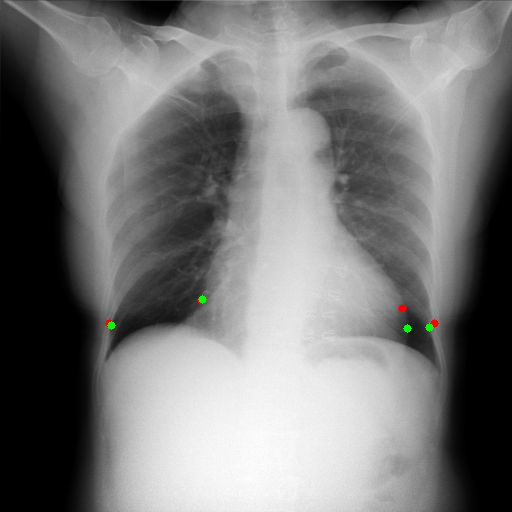

Supplement: Supplementary file 1 [file Presentation1.zip › Supplementary materials/s2/SegNet/13.png]

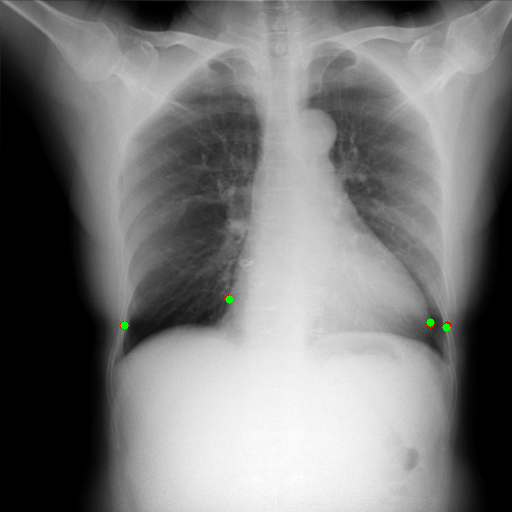

Supplement: Supplementary file 1 [file Presentation1.zip › Supplementary materials/s2/SegNet/14.png]

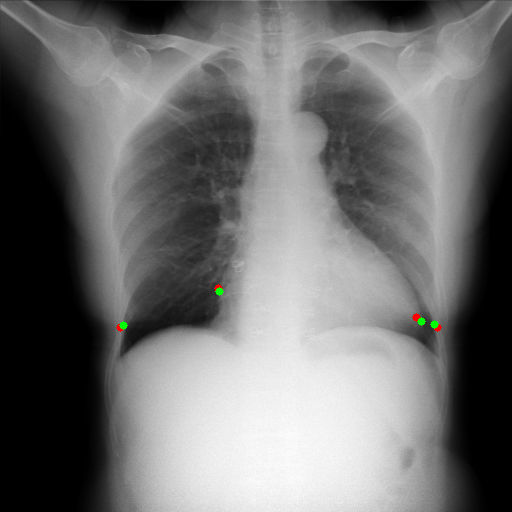

Supplement: Supplementary file 1 [file Presentation1.zip › Supplementary materials/s2/SegNet/15.png]
